# Psychosocial Interventions, Recovery, and Mediating Mechanisms in Schizophrenia-Spectrum Disorders: A Systematic Review and Meta-Analysis of Longitudinal Studies

**DOI:** 10.3390/brainsci16090993

**Published:** 2026-09-19

**Authors:** Evgenia Gkintoni, Ignatia Farmakopoulou, Maria Theodoratou, Maria Panagioti

**Affiliations:** 1 Laboratory of Public Health, Epidemiology & Quality of Life, Department of Medicine, University of Patras, 26504 Patras, Greece; 2 Department of Educational Sciences and Social Work, University of Patras, 26504 Patras, Greece; ifarmakop@upatras.gr; 3 Hellenic Open University, 26335 Patras, Greece; mttheoria3@gmail.com; 4 Division of Population Health, Health Services, Research and Primary Care, University of Manchester, Manchester M13 9PG, UK; maria.panagioti@manchester.ac.uk

**Keywords:** schizophrenia, functional recovery, psychosocial interventions, meta-analysis, longitudinal studies, social cognition, early intervention, personal recovery, mediators, internalized stigma, cognitive remediation

## Abstract

**Highlights:**

**What are the main findings?**

- A hybrid synthesis of 368 longitudinal studies pooled long-term functional recovery at 35.4% (95% CI 22.6–49.4; k = 11) and symptomatic remission at 39.6% (95% CI 29.3–50.5; k = 7); both estimates were robust to leave-one-out and review-inclusive sensitivity analyses but carried very high heterogeneity and low certainty.
- Psychological mediators showed only a weak, imprecise pooled association with functional outcome (r = 0.23; 95% CI −0.09–0.51; very low certainty), because facilitators (positive mental health, social support, self-efficacy, hope) and barriers (internalized stigma, substance use, persistent negative symptoms) act in opposite directions; narratively, structured and combined psychosocial interventions and integrated rehabilitation were associated with small-to-medium functional gains, and social cognition and motivation emerged as salient predictors of functioning.

**What are the implications of the main findings?**

- The proposed Multi-Pathway Dynamic Recovery Model—offered as a testable, hypothesis-generating framework—identifies the first 2–5 years as a critical intervention window and specifies a social-cognitive mediation chain (neurocognition → social cognition/motivation → functional outcome) that may explain the commonly observed cognition–function gap and point to higher-yield treatment targets.
- Services should frontload multicomponent psychosocial intervention from first contact, prioritize social cognition and internalized stigma as treatment targets, and adopt dual-track planning that addresses both clinical and personal recovery alongside structural determinants such as housing and employment.

**Abstract:**

**Background/Objectives**: Recovery from schizophrenia-spectrum disorders is increasingly recognized as achievable, yet no synthesis has simultaneously examined long-term outcome rates, intervention effectiveness, cognitive predictors, social determinants, personal-recovery trajectories, and mediating mechanisms within a unified framework. This systematic review aimed to address these six domains and to propose an integrative theoretical model of functional recovery, applying meta-analysis where the evidence permitted and structured narrative synthesis elsewhere. **Methods**: Seven databases (PubMed/MEDLINE, PsycINFO, Embase, Cochrane CENTRAL, Web of Science, Scopus, CINAHL) were searched through December 2025 following PRISMA 2020 and MOOSE guidance. We extracted eligible records (longitudinal designs, ≥6 months, adults with schizophrenia-spectrum disorders reporting functional outcomes) into two structured databases, then consolidated and de-duplicated them, yielding 467 unique papers. After topical screening and a duplicate audit, we retained 368 studies (1980–2025) and grouped them into six thematic clusters. A pre-specified rule pooled only clusters with at least five independent primary studies. Proportions were pooled with the Freeman–Tukey transformation and associations with Fisher’s z under DerSimonian–Laird random-effects models; certainty was appraised with GRADE. **Results**: Three pools met the threshold. Long-term functional recovery was 35.4% (95% CI 22.6–49.4; k = 11) and symptomatic remission 39.6% (95% CI 29.3–50.5; k = 7), both with very high heterogeneity (I^2^ = 92–98%) and low certainty. The pooled association between psychological mediators and functional outcome was weak and imprecise (r = 0.23; 95% CI −0.09–0.51; k = 7; very low certainty), reflecting facilitators (positive mental health, social support, self-efficacy, hope) and barriers (internalized stigma, substance-use comorbidity, longer duration of untreated psychosis, persistent negative symptoms) acting in opposite directions. The questions concerning specific interventions, cognitive prediction, rehabilitation, and personal recovery were synthesized narratively: structured psychosocial and combined interventions and integrated rehabilitation were associated with small-to-medium functional gains; cognition, particularly social cognition and motivation, predicted later functioning; and personal recovery followed a course partly independent of clinical status. **Conclusions**: Recovery and remission are attainable for a substantial minority but remain heterogeneous and modestly certain. The proposed Multi-Pathway Dynamic Recovery Model organizes the evidence into five testable principles—an ordered recovery hierarchy, social-cognitive mediation of the cognition–function link, an early-phase intervention window, social-ecological embedding, and self-reinforcing feedback loops, offered as a hypothesis-generating framework rather than a validated structure. Services should prioritize comprehensive early intervention, target social cognition and stigma, and address structural determinants to optimize long-term recovery.

## 1. Introduction

### 1.1. The Burden of Schizophrenia-Spectrum Disorders and the Challenge of Long-Term Recovery

Schizophrenia-spectrum disorders (SSDs) remain among the most severe and disabling psychiatric conditions worldwide, affecting approximately 1% of the global population across all cultures and socioeconomic strata. These disorders involve a complex constellation of positive symptoms, negative symptoms, cognitive deficits, and pervasive impairments in social and occupational functioning that often persist throughout the lifespan. Despite more than a century of clinical research and ongoing advances in pharmacological and psychosocial treatment, a substantial minority of individuals diagnosed with SSDs continue to experience chronic disability, dependency on residential care facilities, and significant reductions in life expectancy compared to the general population [1,2,3,4,5,6,7].

Historically, schizophrenia was conceptualized as an inevitably deteriorating disease, epitomized by Kraepelin’s original characterization of dementia praecox. However, longitudinal evidence spanning several decades has progressively challenged this pessimistic perspective. Long-term follow-up studies, some extending to 20–31 years, have demonstrated remarkable heterogeneity in the course and outcomes of SSDs, with a substantial proportion of individuals experiencing periods of significant recovery or sustained improvement. Moreover, researchers have also established that schizophrenia does not conform to a uniformly progressive trajectory and that meaningful clinical, functional, and personal recovery is achievable for a sizeable proportion of patients [8,9].

Nevertheless, the gap between symptomatic remission and functional recovery remains a central clinical challenge. Meta-analytic evidence indicates that while remission from positive symptoms is attained by a considerable proportion of first-episode psychosis (FEP) patients, full functional recovery, encompassing independent living, sustained employment, satisfactory social relationships, and subjective quality of life, is achieved by a smaller percentage. Longitudinal cohort studies consistently report that only 13–52% of FEP patients achieve functional recovery, depending on the criteria used and the follow-up duration. This discrepancy underscores the need for interventions that target not only symptom reduction but also the broader determinants of real-world functioning and personal well-being [10,11,12,13,14,15,16,17].

### 1.2. Psychosocial Interventions: From Evidence-Based Practice to Long-Term Functional Outcomes

Over the past three decades, psychosocial interventions have become indispensable components of comprehensive SSD treatment, complementing pharmacological management and addressing the multifaceted needs of individuals across the illness trajectory. These interventions span a wide continuum, from early-intervention services (EIS) for first-episode psychosis to cognitive-behavioral therapy (CBT), cognitive remediation therapy (CRT), family-based interventions, supported employment programs, social skills training, metacognitive therapies, peer-led health promotion initiatives, and digitally enhanced monitoring and treatment delivery systems [18,19,20].

Early-intervention services, which typically integrate pharmacological management with psychosocial support within a specialized multidisciplinary team, have demonstrated robust short-term benefits in reducing symptom severity, improving social functioning, and decreasing hospitalization rates during the critical period following the first episode. However, evidence on the sustainability of these gains beyond the initial two-to-five-year treatment window remains inconsistent, with some studies reporting attenuation of benefits after discharge from specialized services, while others document enduring positive effects on functional trajectories over 10 or more years of follow-up.

Psychological therapies specifically targeting the cognitive, metacognitive, and affective dimensions of psychotic experience have likewise expanded in both scope and empirical support. Metacognitive narrative psychotherapy, avatar-based therapy for auditory hallucinations, ecological momentary interventions, and social cognition training aim to enhance self-reflective capacities, reduce distressing symptoms, and promote functional recovery by engaging with real-world psychological mechanisms. Furthermore, lifestyle and wellness interventions addressing the well-documented physical health disparities in SSD populations have gained increasing attention, recognizing that metabolic and cardiovascular comorbidities contribute substantially to the reduced life expectancy observed in this group [21,22,23,24,25,26,27].

Despite this proliferation of psychosocial treatment approaches, the evidence base linking specific interventions to long-term functional recovery outcomes in SSDs remains fragmented. Individual studies vary considerably in follow-up duration, outcome measurement, population characteristics, and methodological rigor, making it difficult for clinicians and policymakers to draw definitive conclusions about which interventions most effectively promote sustained functional improvement across the illness course [28,29,30,31,32].

### 1.3. Cognitive Functioning as a Predictor and Mediator of Functional Recovery

Cognitive impairment is one of the most robust predictors of functional outcome in SSDs, with deficits in domains such as verbal memory, executive functioning, processing speed, attention, and social cognition consistently associated with poorer real-world functioning across the illness trajectory. Longitudinal studies have demonstrated that neurocognitive performance at illness onset predicts social and occupational functioning years to decades later, with some evidence suggesting that cognitive deficits may mediate the relationship between clinical symptoms and community-level functional outcomes [33,34,35,36,37,38,39,40,41,42].

The relationship between cognitive functioning and functional recovery has generated particular interest as a potential target for psychosocial intervention. Cognitive remediation programs, social cognition training, and integrated approaches that combine cognitive and vocational rehabilitation have shown promise in improving both cognitive performance and downstream functional outcomes. However, the magnitude and durability of these effects, and the specific cognitive domains most critical for predicting longitudinal functional trajectories, remain subjects of active investigation. Moreover, motivational deficits—increasingly recognized as a central component of negative symptoms—have been shown to moderate the relationship between neurocognitive capacity and real-world performance, suggesting that the pathway from cognitive ability to functional recovery is more complex than initially conceptualized [43,44,45,46,47,48,49].

### 1.4. Psychosocial Rehabilitation, Social Functioning, and Community Integration

Functional recovery in SSDs extends beyond clinical remission and cognitive performance to encompass a broad range of real-world outcomes, including competitive employment, stable housing, social participation, and community integration. Psychosocial rehabilitation approaches—particularly supported employment models such as Individual Placement and Support (IPS)—have accumulated substantial evidence for improving vocational outcomes in individuals with severe mental illness. Longitudinal studies have documented significant increases in employment rates among individuals receiving rehabilitation services, with predictors of vocational success including prior work experience, educational attainment, cognitive capacity, and baseline social functioning [50,51,52,53,54,55,56].

Yet vocational and social outcomes remain profoundly influenced by broader social determinants of health, including socioeconomic status, neighborhood-level factors, substance-use comorbidity, exposure to violence and trauma, family environment, and access to integrated community-based services. Scoping reviews of social determinants in SSD populations have highlighted the complex interplay between these factors and clinical outcomes, emphasizing that intersecting social disadvantages—including poverty, discrimination, and limited access to healthcare—exert enduring effects on long-term vocational and social functioning. The expressed-emotion literature has further underscored the role of family dynamics, with high levels of criticism and emotional over-involvement associated with increased relapse risk and poorer functional outcomes [57,58,59,60,61,62,63].

Community-based models of care, including assertive community treatment, integrated mental health and vocational programs, and consumer/survivor initiatives, have been developed to address these multifaceted barriers to recovery. However, evidence on the long-term effectiveness of these models in promoting sustained community integration and functional independence remains mixed, with service intensity, continuity of care, and the specific composition of rehabilitation programs all influencing outcomes [64,65,66,67,68,69].

### 1.5. Personal Recovery, Quality of Life, and Well-Being Trajectories

The recovery paradigm in mental health has undergone a fundamental transformation over the past two decades, shifting from a predominantly biomedical model focused on symptom remission toward a person-centered framework that emphasizes personal recovery—defined as the process of developing new meaning and purpose in life beyond the limitations imposed by mental illness. This reconceptualization has been driven by both the consumer/survivor movement and a growing body of longitudinal research demonstrating that clinical and personal recovery represent related but distinct outcome domains, with different trajectories, predictors, and implications for service delivery [70,71,72,73,74,75]. To avoid conflation, we distinguish three constructs used throughout: symptomatic remission (sustained attainment of a symptom-severity threshold over a specified minimum duration), functional recovery (sustained role, social, and independent-living functioning, with or without concurrent symptom criteria), and personal recovery (the subjective, process-oriented experience just described), which our findings indicate can follow a course partly independent of clinical status.

Longitudinal studies examining personal recovery and subjective quality of life (QoL) in SSD populations have revealed complex patterns of change over time. While symptom improvement and functional gains may follow relatively linear trajectories, personal recovery—as captured by instruments such as the Questionnaire about the Process of Recovery (QPR), the Recovery Assessment Scale (RAS), and the Stages of Recovery Instrument (STORI)—often exhibits a more dynamic and nonlinear course, influenced by factors such as hope, self-efficacy, social support, and the subjective meaning attributed to the illness experience. Studies have reported that flourishing and high levels of well-being are attainable even with ongoing symptoms, suggesting that clinical recovery and personal recovery need not be sequential or mutually exclusive [76,77,78,79,80,81].

The relationship between symptomatic remission and personal recovery has been a growing focus of inquiry. Evidence indicates that while symptom reduction contributes to improved QoL and personal recovery, it is neither sufficient nor necessary for individuals to report meaningful improvements in their subjective well-being and sense of agency. Factors such as self-esteem, resilience, hope, empowerment, and the perception of being able to manage one’s own condition have emerged as key determinants of personal-recovery trajectories, independent of clinical status [82,83,84,85,86].

### 1.6. Psychological Mediators, Resilience, and Health Promotion Mechanisms

Understanding how psychosocial interventions affect long-term functional recovery is critical for optimizing treatment design and delivery. A growing body of evidence has identified several psychological factors that mediate or moderate the relationship between intervention engagement and recovery outcomes. The most extensively studied include internalized stigma, clinical insight, self-esteem, motivation, attachment style, locus of control, and psychological resilience [87,88,89,90,91,92,93,94,95].

Stigma—both experienced and internalized—has been consistently associated with poorer quality of life, reduced help-seeking behavior, and diminished engagement with treatment in individuals with SSDs. Network meta-analytic evidence suggests that psychological interventions targeting personal stigma, including psychoeducation, CBT-based approaches, and peer-contact strategies, can meaningfully reduce self-stigma and improve self-esteem and QoL, although the sustainability of these effects over extended follow-up periods remains uncertain [96,97,98,99].

Clinical insight presents a more complex picture, as increased awareness of illness has been associated with both improved medication adherence and therapeutic engagement and with higher levels of depressive symptomatology and self-stigma—a phenomenon described as the “insight paradox.” Longitudinal studies have begun to disentangle this relationship, examining how changes in insight interact with self-stigma processes to influence life satisfaction and functional outcomes over time [100,101,102,103,104,105,106,107,108].

Resilience—conceptualized as the dynamic process of positive adaptation in the face of significant adversity—has emerged as an important framework for understanding individual differences in recovery trajectories. Longitudinal research has identified resilience-related traits, including optimism, emotional regulation, and social connectedness, as predictive of better health outcomes in individuals living with schizophrenia across the lifespan. Positive psychiatry and health-promotion frameworks have further expanded this perspective, advocating for interventions that cultivate psychological assets and protective factors alongside addressing symptoms and functional deficits. Post-traumatic growth research has also documented that individuals with psychosis can experience positive psychological change as a consequence of their illness experience, provided that appropriate therapeutic support is available [109,110,111,112,113,114,115].

### 1.7. Gaps in the Literature and Rationale for the Present Review

Despite the substantial and growing body of research on psychosocial interventions and long-term outcomes in SSDs, several critical gaps remain unaddressed through systematic synthesis and quantitative integration. First, prior syntheses have tended to focus on specific intervention modalities, illness phases, or outcome domains in isolation, limiting cross-domain comparison and the identification of shared mechanisms of therapeutic change. Second, researchers have quantified the long-term course of SSDs—particularly the distinction between symptomatic remission and functional recovery—inconsistently across heterogeneous cohorts and recovery definitions. Third, researchers have investigated the psychological mediators and moderators of recovery, including stigma, insight, self-esteem, resilience, and motivation, piecemeal rather than within a common analytic framework [116,117,118,119,120,121,122,123,124,125,126,127].

A further methodological gap concerns the differing evidentiary maturity of these questions. The present review is explicit about this heterogeneity: the long-term-course outcomes (recovery and remission proportions) and the mediator–outcome associations are supported by sufficient independent quantitative data to permit meta-analytic pooling, whereas the questions concerning specific intervention effects, cognitive prediction, rehabilitation, and personal-recovery trajectories are at present better served by structured narrative synthesis, because the available longitudinal evidence reports too few independent, comparably measured effect sizes to support defensible pooling. Rather than forcing uniform meta-analysis onto evidence of unequal density—an approach that risks spuriously precise estimates—this review adopts a hybrid design: meta-analysis where the data warrant it, and transparent narrative synthesis elsewhere. To maximize comprehensiveness and auditability, we assembled the evidence base by consolidating and de-duplicating two independently compiled extraction databases into a single corpus [128,129,130,131,132,133,134,135,136,137,138,139].

### 1.8. Aims and Research Questions

The present systematic review and meta-analysis aims to address these gaps by providing a comprehensive and methodologically transparent synthesis of the longitudinal evidence on psychosocial interventions and long-term functional recovery in schizophrenia-spectrum disorders. Drawing on a consolidated, de-duplicated corpus of 467 records (377 meeting eligibility criteria) spanning more than three decades of research, the review integrates evidence across six thematic domains, each corresponding to a core dimension of the psychosocial intervention–recovery relationship. Consistent with the rationale above, we address two questions meta-analytically (RQ1, reported as two distinct outcomes—functional recovery and symptomatic remission—and RQ6) and four narratively; this division was specified a priori and is reflected throughout the analysis and reporting (Table 1). The guiding research questions are:

RQ1 (Long-Term Course and Outcome—meta-analytic): What is the long-term course and outcome of schizophrenia-spectrum disorders in terms of (a) functional recovery and (b) symptomatic remission, how do these two rates compare, and how robust are the pooled estimates to differences in definition, study design, and follow-up duration?

RQ2 (Psychological and Combined Treatment Approaches—narrative): What are the comparative long-term effects of psychosocial intervention modalities—including cognitive-behavioral therapy, metacognitive therapy, family-based interventions, lifestyle programs, digital health interventions, and combined psychosocial–pharmacological approaches—on functional recovery, symptom reduction, and quality of life?

RQ3 (Cognitive Predictors of Functional Recovery—narrative): To what extent does baseline neurocognitive and social-cognitive functioning—across domains including verbal memory, executive function, processing speed, and social cognition—predict longitudinal functional outcomes, and do interventions targeting cognition moderate this relationship?

RQ4 (Psychosocial Rehabilitation and Community Integration—narrative): What is the effectiveness of psychosocial rehabilitation programs—including supported employment, integrated community treatment, and consumer-led initiatives—for long-term vocational functioning, social participation, and community integration, and how do social determinants, substance-use comorbidity, and family environment moderate these outcomes?

RQ5 (Personal Recovery and Quality of Life—narrative): What are the longitudinal trajectories of personal recovery and subjective quality of life, and which psychosocial, clinical, and individual-level factors—including hope, self-efficacy, and the remission–personal-recovery inter-relationship—predict sustained patient-centered recovery?

RQ6 (Psychological Mediators and Resilience—meta-analytic): What is the strength and direction of the association between psychological mediators—internalized stigma, clinical insight, self-esteem, motivation, locus of control, and resilience—and long-term functional outcomes across longitudinal studies?

By pairing meta-analytic pooling, where the evidence is sufficient, with transparent narrative synthesis, where it is not, the present review aims to provide a comprehensive, clinically actionable, and methodologically honest account of long-term recovery in SSDs. The findings are intended to inform evidence-based clinical practice, service design, and future research priorities in the recovery-oriented management of schizophrenia-spectrum disorders. The remainder of this manuscript is organized as follows. Section 2 describes the methodology, including the consolidated search and screening provenance, eligibility criteria, data-extraction procedures, risk-of-bias assessment, and the meta-analytic approaches applied to RQ1 (functional recovery and symptomatic remission) and RQ6.

## 2. Materials and Methods

### 2.1. Protocol Registration and Reporting Standards

This systematic review and meta-analysis were conducted and reported in accordance with the Preferred Reporting Items for Systematic Reviews and Meta-Analyses (PRISMA) 2020 statement [140] and the Meta-analysis of Observational Studies in Epidemiology (MOOSE) reporting standards [141]. A central design decision, specified a priori, was to adopt a hybrid synthesis model: meta-analytic pooling was applied only to questions supported by sufficient independent quantitative evidence, while the remaining questions were addressed through structured narrative synthesis.

### 2.2. Eligibility Criteria

Studies were eligible if they: (a) included adults diagnosed with schizophrenia-spectrum disorders (SSDs), schizophrenia, schizoaffective disorder, schizophreniform disorder, delusional disorder, first-episode psychosis (FEP), or psychosis not otherwise specified, according to standardized diagnostic criteria (DSM-III through DSM-5; ICD-9 or ICD-10); (b) examined at least one psychosocial intervention, exposure, or determinant in relation to functional outcomes; (c) reported at least one quantitative measure of long-term functional recovery, social or vocational functioning, personal recovery, quality of life, or clinical remission alongside functional data; and (d) employed a longitudinal design with a minimum follow-up of six months.

Eligible interventions included early-intervention services (EIS), cognitive-behavioral therapy for psychosis (CBTp), family-based interventions, cognitive remediation therapy (CRT), social cognition training, supported employment and Individual Placement and Support (IPS), social skills training, peer-led programs, assertive community treatment (ACT), combined psychosocial–pharmacological approaches, and recovery-oriented service models. Eligible designs comprised randomized controlled trials (RCTs), quasi-experimental studies, longitudinal cohort studies (prospective and retrospective), naturalistic follow-up studies, and systematic reviews/meta-analyses. Reviews and meta-analyses were retained for narrative description and sensitivity analysis, but to preserve independence, were excluded from the primary quantitative pools (Section 2.8). Peer-reviewed English-language publications up to December 2025 were eligible; conference abstracts, protocols, and editorials were excluded.

Studies were excluded if they focused exclusively on non-psychotic populations, reported only cross-sectional data, lacked quantifiable functional or recovery outcomes, or could not be obtained in full text despite reasonable efforts.

### 2.3. Information Sources and Search Strategy

We searched seven electronic databases from inception through December 2025: PubMed/MEDLINE, PsycINFO (EBSCO), Embase (Ovid), Cochrane CENTRAL, Web of Science, Scopus, and CINAHL. The search strategy, developed in consultation with an information specialist, combined four conceptual blocks with Boolean operators: (1) population terms (schizophrenia-spectrum and psychosis keywords), (2) intervention/exposure terms (psychosocial, cognitive, vocational, and rehabilitation keywords), (3) outcome terms (functional recovery, quality of life, employment, and related domains), and (4) design terms (longitudinal, follow-up, RCT, meta-analysis). We provide complete search strategies in Supplementary Materials. Supple-mentary methods included backward and forward citation tracking, hand-searching of ten specialty journals (including Schizophrenia Bulletin, Schizophrenia Research, Psy-chological Medicine, and Psychiatric Services), and contact with subject-matter experts.

### 2.4. Study Selection, Corpus Consolidation, and De-Duplication

Two independent reviewers screened retrieved records in two stages using Rayyan and Covidence [142,143]: title/abstract screening followed by full-text assessment, with disagreements resolved by discussion or a third reviewer. Topical screening denotes independent assessment of each record for relevance to longitudinal outcomes of schizophrenia-spectrum disorders. We quantified inter-rater agreement for screening using Cohen’s κ [144], interpreted by Landis and Koch thresholds [145], and found it high (κ = 0.84).

We extracted eligible studies into two structured databases compiled in separate passes (an 87-field and an 83-field instrument). Because these databases were largely distinct rather than nested, we concatenated them, de-duplicated by Digital Object Identifier, and normalized titles. Of the 550 combined records, 83 were shared, yielding a consolidated corpus of 467 unique papers (267 from the first database only, 117 from the second only, and 83 in both). For data extraction, we independently double-extracted a random 15% of studies (≥90% agreement), and we additionally verified every pooled-study value against the source text in the extraction-integrity audit (Table S8).

**Figure 1:**
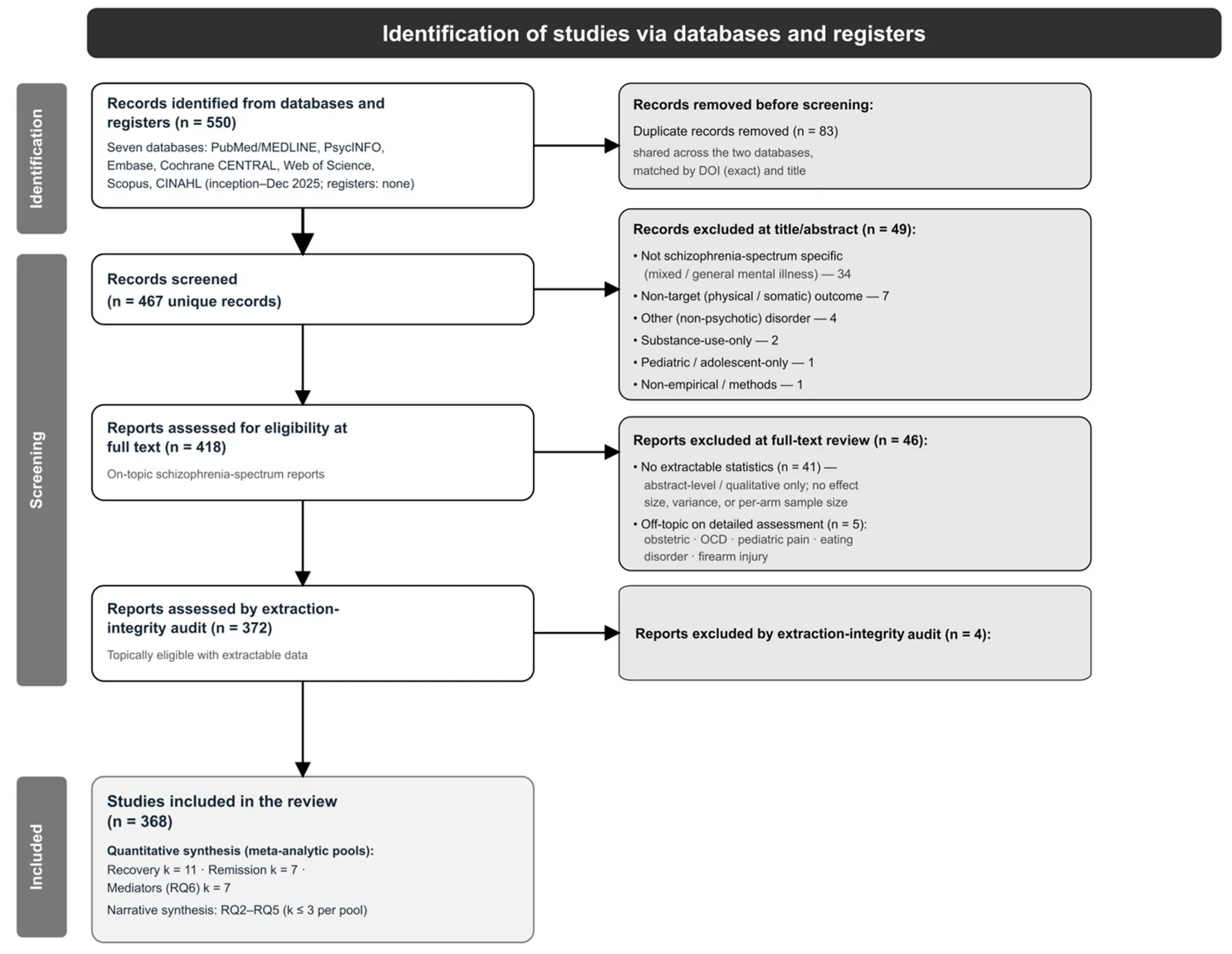
Corpus consolidation and study-selection provenance. Two extraction databases were consolidated and de-duplicated to 467 unique papers; successive screening for topical fit and extractable statistics, and a detailed off-topic screen, yielded 368 included studies (references [171,172,173,174,175,176,177,178,179,180,181,182,183,184,185,186,187,188,189,190,191,192,193,194,195,196,197,198,199,200,201,202,203,204,205,206,207,208,209,210,211,212,213,214,215,216,217,218,219,220,221,222,223,224,225,226,227,228,229,230,231,232,233,234,235,236,237,238,239,240,241,242,243,244,245,246,247,248,249,250,251,252,253,254,255,256,257,258,259,260,261,262,263,264,265,266,267,268,269,270,271,272,273,274,275,276,277,278,279,280,281,282,283,284,285,286,287,288,289,290,291,292,293,294,295,296,297,298,299,300,301,302,303,304,305,306,307,308,309,310,311,312,313,314,315,316,317,318,319,320,321,322,323,324,325,326,327,328,329,330,331,332,333,334,335,336,337,338,339,340,341,342,343,344,345,346,347,348,349,350,351,352,353,354,355,356,357,358,359,360,361,362,363,364,365,366,367,368,369,370,371,372,373,374,375,376,377,378,379,380,381,382,383,384,385,386,387,388,389,390,391,392,393,394,395,396,397,398,399,400,401,402,403,404,405,406,407,408,409,410,411,412,413,414,415,416,417,418,419,420,421,422,423,424,425,426,427,428,429,430,431,432,433,434,435,436,437,438,439,440,441,442,443,444,445,446,447,448,449,450,451,452,453,454,455,456,457,458,459,460,461,462,463,464,465,466,467,468,469,470,471,472,473,474,475,476,477,478,479,480,481,482,483,484,485,486,487,488,489,490,491,492,493,494,495,496,497,498,499,500,501,502,503,504,505,506,507,508,509,510,511,512,513,514,515,516,517,518,519,520,521,522,523,524,525,526,527,528,529,530,531,532,533,534,535,536,537,538]). Application of the pre-specified synthesis-mode rule produced three quantitative pools of independent primary studies (recovery k = 11, remission k = 7, mediators k = 7); RQ2–RQ5 were synthesized narratively.

This consolidated, de-duplicated corpus, rather than either source file alone, constitutes the auditable basis for screening and synthesis. Topical screening removed 49 off-topic records, leaving 418 on-topic papers, of which 377 reported extractable or inferential statistics. Then, we judged a further five records off-topic on full-text assessment; removing these nine records yielded the final 368 included studies. All exclusions were assessed independently by two reviewers. Section 3.1 and Supplementary Table S7 (PRISMA 2020 checklist) report the full screening provenance, and Figure 1 depicts the flow.

### 2.5. Data Extraction

Extraction proceeded in two tiers. The first tier captured study identification, population, design, intervention, and outcome descriptors at the abstract level using standardized instruments (piloted a priori, with discrepancies resolved by a third reviewer); data were extracted at all available time points to capture the longest follow-up period. The second tier, applied to studies forming each quantitative pool, sought the statistics required for pooling: proportions and denominators for binary outcomes (recovery, remission); Pearson correlations and sample sizes for predictor–outcome associations; and means, standard deviations, or convertible test statistics for continuous outcomes. Where only test statistics (F, t, χ^2^) and sample sizes were available, effect sizes were computed using established conversion formulae [146,147,148,149,150,151,152,153,154,155,156,157,158]. Supplementary Table S8 provides the audit trail for every extracted statistic, its source field, extracted value, and verbatim excerpt.

### 2.6. Thematic Clustering and Synthesis-Mode Assignment

Each study was assigned to one of six thematic clusters corresponding to the review’s research questions using a keyword taxonomy (title terms weighted threefold), with two reviewers resolving disagreements by consensus: TC1—Long-Term Course and Outcome (RQ1); TC2—Psychological and Combined Interventions (RQ2); TC3—Cognitive Predictors of Functional Recovery (RQ3); TC4—Psychosocial Rehabilitation and Community Integration (RQ4); TC5—Personal Recovery, Quality of Life, and Well-Being (RQ5); and TC6—Psychological Mediators, Resilience, and Health Promotion (RQ6). We then determined the synthesis mode for each cluster using a pre-specified rule: we synthesized a cluster meta-analytically only if it included at least five independent primary studies reporting a comparably measured effect size; otherwise, we synthesized it narratively. This rule yielded two meta-analytic questions—RQ1 (analyzed as two distinct outcomes, long-term functional recovery and symptomatic remission) and RQ6 (mediator–outcome associations), and four narratively synthesized questions (RQ2–RQ5) (Table 2).

### 2.7. Risk-of-Bias Assessment

Risk of bias was assessed with design-appropriate tools: the revised Cochrane RoB 2 tool [146] for trials (five domains: randomization, deviations, missing data, outcome measurement, selective reporting), a Newcastle–Ottawa Scale appraisal of selection, comparability [147], and outcome ascertainment for non-randomized longitudinal studies, and AMSTAR 2 [148] for included reviews. Two reviewers assessed each study independently and resolved disagreements by consensus; we report the final study-level ratings in Supplementary Table S4 and incorporated them into the certainty appraisal (Section 2.10).

### 2.8. Independence and Publication Bias

To preserve independence, we restricted primary pools to independent primary studies; we excluded systematic reviews and meta-analyses from the primary estimates and included them only as a pre-specified sensitivity comparison (Section 2.9.3). When multiple effect sizes were derived from the same source cohort, we retained a single estimate per cohort. Formal small-study/publication-bias procedures (contour-enhanced funnel plots [149], Egger’s regression [150], Begg–Mazumdar [151], and trim-and-fill [152]) were planned for pools of at least ten studies; given the modest pool sizes and the very high heterogeneity observed (I^2^ ≈ 92–100%), these diagnostics were considered uninterpretable and were not relied upon for inference.

### 2.9. Data Synthesis and Meta-Analytic Procedures

#### 2.9.1. Effect Size Metrics

We applied two metrics to the three quantitative pools. For binary course outcomes (functional recovery and symptomatic remission), we pooled proportions after applying the Freeman–Tukey double-arcsine transformation [155] and back-transformed for reporting using the harmonic mean of the sample sizes. For mediator–outcome associations (RQ6), we transformed Pearson’s r to Fisher’s z for pooling and back-transformed to r. Continuous between-group effects, where encountered in the narrative clusters, were expressed as Hedges’ g [152]; these were summarized descriptively rather than pooled.

#### 2.9.2. Random-Effects Models and Heterogeneity

All pools used random-effects models with the DerSimonian–Laird τ^2^ estimator [156,157]. Pooled estimates are reported with 95% confidence intervals and 95% prediction intervals; heterogeneity was quantified using the I^2^ statistic [158], Cochran’s Q [159], and τ^2^. Given the consistently very high heterogeneity, estimates are interpreted as average effects across heterogeneous populations rather than as point estimates of a common parameter.

#### 2.9.3. Sensitivity and Influence Analyses

The robustness of each proportion pool was examined through (a) leave-one-out re-estimation; (b) alternative model specifications—fixed-effect inverse-variance and the Hartung–Knapp adjustment [160]; (c) study-level influence diagnostics (inverse-variance weights and a Cook’s distance analog); and (d) a comparison of the primary independent-study estimate against an additionally incorporating the available reviews and meta-analyses. Supplementary Table S6a–c report full diagnostics.

#### 2.9.4. Analyses Planned but Not Conducted

Mixed-effects meta-regression and subgroup moderator analyses were pre-specified but not conducted, because the pools (k = 7–11) did not meet the planned criterion of at least ten studies for stable moderator estimation. Likewise, we planned a two-stage structural-equation-modeling (TSSEM) [161] mediation analysis for RQ6, but insufficient data prevented us from assembling a pooled correlation matrix; RQ6 was therefore synthesized as bivariate mediator–outcome associations [162,163,164,165]. We documented these deviations in Supplementary Materials and identified them as such.

### 2.10. Certainty of Evidence

We rated the certainty of each pooled estimate using the GRADE framework [162], considering risk of bias, inconsistency, indirectness, imprecision, and publication bias; ratings are summarized in Supplementary Table S5. Narratively synthesized questions (RQ2–RQ5) were not assigned pooled certainty ratings and are reported descriptively.

### 2.11. Software

All analyses were conducted in R version 4.3.2 (R Foundation for Statistical Computing, Vienna, Austria) [166] using the *metafor* (v4.6-0) [167], *meta* (v7.0-0) [163], and *robumeta* (v2.1) [168] packages, with figures produced in *ggplot2* (v3.5.1) [169] for visualization; proportions used the double-arcsine transformation and associations the Fisher-z transformation as implemented therein. Robust variance estimation (robumeta [168]) was available for dependent effect sizes but was not required, as the primary pools comprised independent primary studies. Procedures followed the Cochrane Handbook [170], and the complete analysis script is provided as a Supplementary File; all pooled estimates regenerate directly from Supplementary Tables S1 and S8.

Application of this methodology to the consolidated corpus (467 unique records; 377 meta-analysis-eligible; publications 1980–2025; follow-up 6 months–31 years) yielded three quantitative pools, long-term recovery (k = 11), symptomatic remission (k = 7), and mediator–outcome associations (k = 7), with the remaining questions synthesized narratively. The results are presented in Section 3.

## 3. Results

### 3.1. Study Selection and Provenance

Eligible studies retrieved through the database search (Section 2.3) were extracted into two structured databases that, on inspection, proved largely distinct rather than nested. Concatenation and de-duplication by DOI and normalized title (550 combined records, 83 shared) produced a consolidated corpus of 467 unique papers. Topical screening removed 49 off-topic records, leaving 418 on-topic papers, of which 377 reported extractable or inferential statistics. A subsequent detailed screen of these 377 records identified four residual duplicate records that were removed in a final reference audit, and five further studies whose populations or outcomes fell outside the schizophrenia-spectrum scope (e.g., obsessive–compulsive, eating-disorder, obstetric, and injury cohorts); their removal yielded a final synthesis set of 368 studies, numbered sequentially by research question as references [171,172,173,174,175,176,177,178,179,180,181,182,183,184,185,186,187,188,189,190,191,192,193,194,195,196,197,198,199,200,201,202,203,204,205,206,207,208,209,210,211,212,213,214,215,216,217,218,219,220,221,222,223,224,225,226,227,228,229,230,231,232,233,234,235,236,237,238,239,240,241,242,243,244,245,246,247,248,249,250,251,252,253,254,255,256,257,258,259,260,261,262,263,264,265,266,267,268,269,270,271,272,273,274,275,276,277,278,279,280,281,282,283,284,285,286,287,288,289,290,291,292,293,294,295,296,297,298,299,300,301,302,303,304,305,306,307,308,309,310,311,312,313,314,315,316,317,318,319,320,321,322,323,324,325,326,327,328,329,330,331,332,333,334,335,336,337,338,339,340,341,342,343,344,345,346,347,348,349,350,351,352,353,354,355,356,357,358,359,360,361,362,363,364,365,366,367,368,369,370,371,372,373,374,375,376,377,378,379,380,381,382,383,384,385,386,387,388,389,390,391,392,393,394,395,396,397,398,399,400,401,402,403,404,405,406,407,408,409,410,411,412,413,414,415,416,417,418,419,420,421,422,423,424,425,426,427,428,429,430,431,432,433,434,435,436,437,438,439,440,441,442,443,444,445,446,447,448,449,450,451,452,453,454,455,456,457,458,459,460,461,462,463,464,465,466,467,468,469,470,471,472,473,474,475,476,477,478,479,480,481,482,483,484,485,486,487,488,489,490,491,492,493,494,495,496,497,498,499,500,501,502,503,504,505,506,507,508,509,510,511,512,513,514,515,516,517,518,519,520,521,522,523,524,525,526,527,528,529,530,531,532,533,534,535,536,537,538]. Inter-rater agreement at screening was strong (κ = 0.84), and a post-consolidation audit resolved one residual duplicate to a single entry. The complete provenance is depicted in Figure 1.

Application of the pre-specified synthesis-mode rule (Section 2.6) to this corpus yielded three quantitative pools, long-term functional recovery (k = 11), symptomatic remission (k = 7), and mediator–outcome associations (k = 7), with the remaining four questions meeting the threshold for narrative synthesis only. Table 3 reconciles each stage of the funnel and maps the reference ranges to research questions.

### 3.2. Characteristics of Included Studies

The 368 included studies (references [171,172,173,174,175,176,177,178,179,180,181,182,183,184,185,186,187,188,189,190,191,192,193,194,195,196,197,198,199,200,201,202,203,204,205,206,207,208,209,210,211,212,213,214,215,216,217,218,219,220,221,222,223,224,225,226,227,228,229,230,231,232,233,234,235,236,237,238,239,240,241,242,243,244,245,246,247,248,249,250,251,252,253,254,255,256,257,258,259,260,261,262,263,264,265,266,267,268,269,270,271,272,273,274,275,276,277,278,279,280,281,282,283,284,285,286,287,288,289,290,291,292,293,294,295,296,297,298,299,300,301,302,303,304,305,306,307,308,309,310,311,312,313,314,315,316,317,318,319,320,321,322,323,324,325,326,327,328,329,330,331,332,333,334,335,336,337,338,339,340,341,342,343,344,345,346,347,348,349,350,351,352,353,354,355,356,357,358,359,360,361,362,363,364,365,366,367,368,369,370,371,372,373,374,375,376,377,378,379,380,381,382,383,384,385,386,387,388,389,390,391,392,393,394,395,396,397,398,399,400,401,402,403,404,405,406,407,408,409,410,411,412,413,414,415,416,417,418,419,420,421,422,423,424,425,426,427,428,429,430,431,432,433,434,435,436,437,438,439,440,441,442,443,444,445,446,447,448,449,450,451,452,453,454,455,456,457,458,459,460,461,462,463,464,465,466,467,468,469,470,471,472,473,474,475,476,477,478,479,480,481,482,483,484,485,486,487,488,489,490,491,492,493,494,495,496,497,498,499,500,501,502,503,504,505,506,507,508,509,510,511,512,513,514,515,516,517,518,519,520,521,522,523,524,525,526,527,528,529,530,531,532,533,534,535,536,537,538]; Supplementary Tables S1 and S2) were published between 1980 and 2025 (median 2017) and reported follow-up durations from 6 months to 31 years. Longitudinal cohort and naturalistic follow-up designs predominated, complemented by randomized and quasi-experimental trials and a minority of systematic reviews and meta-analyses; we retained the latter only for narrative and sensitivity analyses. The corpus was distributed unevenly across the six clusters, with the long-term-course (RQ1, n = 115) and mediator (RQ6, n = 87) literature clusters the largest, consistent with the field’s sustained attention to prognosis and to the psychological correlates of outcome. Findings are presented by research question, beginning with the two pooled course outcomes, then proceeding through the narratively synthesized domains, and finally to the pooled mediator analysis.

### 3.3. RQ1: Long-Term Course and Outcome [171,172,173,174,175,176,177,178,179,180,181,182,183,184,185,186,187,188,189,190,191,192,193,194,195,196,197,198,199,200,201,202,203,204,205,206,207,208,209,210,211,212,213,214,215,216,217,218,219,220,221,222,223,224,225,226,227,228,229,230,231,232,233,234,235,236,237,238,239,240,241,242,243,244,245,246,247,248,249,250,251,252,253,254,255,256,257,258,259,260,261,262,263,264,265,266,267,268,269,270,271,272,273,274,275,276,277,278,279,280,281,282,283]

RQ1 was addressed through two distinct pooled outcomes, functional recovery and symptomatic remission, reported separately and never combined into a single estimate, alongside a narrative synthesis of illness trajectories and prognostic factors. A recurring observation across the cluster was that the course is heterogeneous and multi-class rather than uniform: latent-trajectory analyses repeatedly resolved the patient population into three or more distinct outcome paths, a structure that frames the subsequent pooled rates.

#### 3.3.1. Functional Recovery

**Figure 2:**
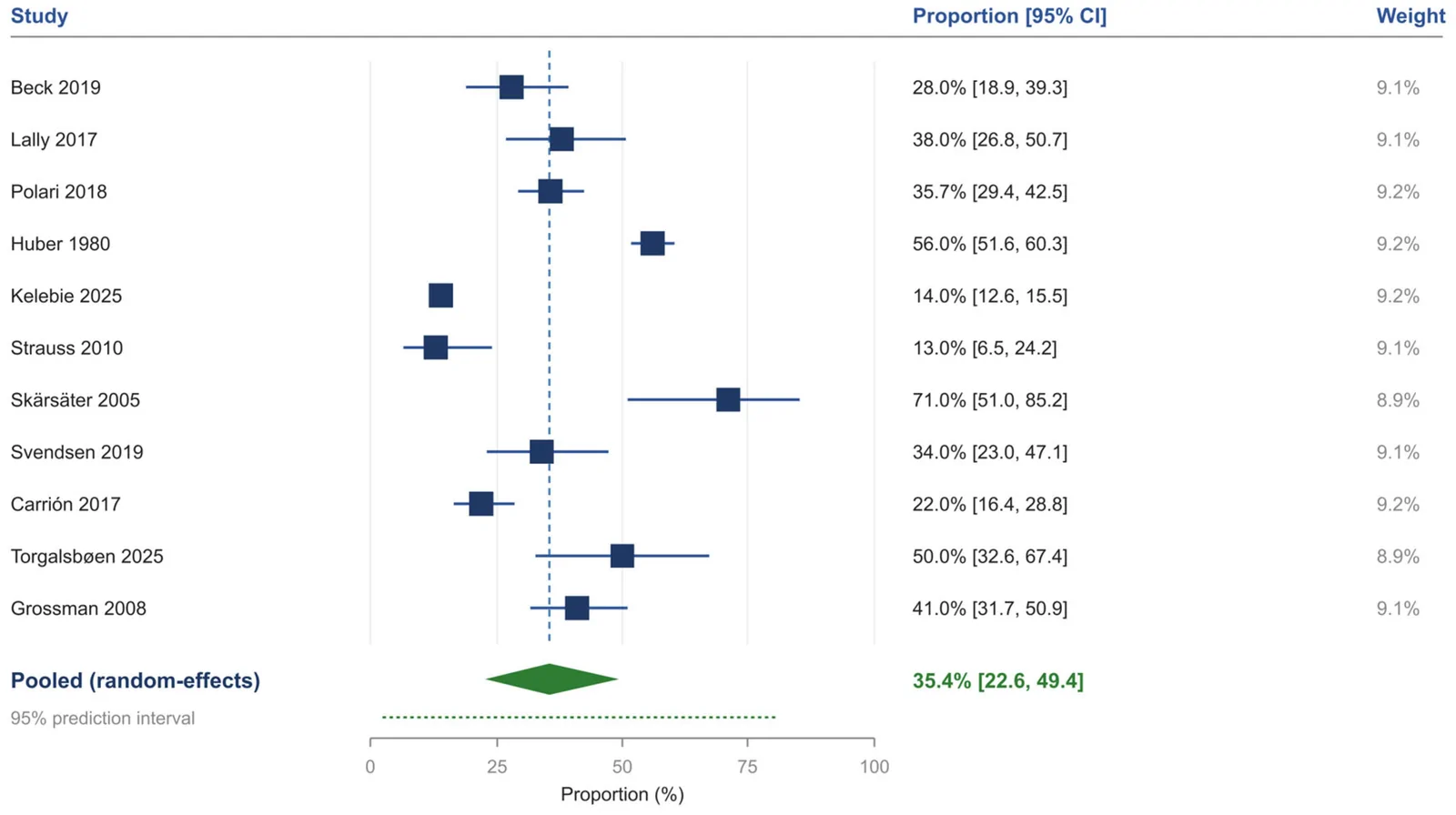
A random-effects forest plot of long-term functional recovery proportions (k = 11; N = 3531). The squares are study estimates (size proportional to weight) with 95% confidence intervals; the diamond is the pooled Freeman–Tukey estimate, 35.4% (95% CI 22.6–49.4%); the dotted line is the 95% prediction interval. I^2^ = 98%. Included studies: Beck 2019 [178], Carrión 2017 [184], Grossman 2008 [208], Huber 1980 [217], Kelebie 2025 [226], Lally 2017 [230], Polari 2018 [245], Skärsäter 2005 [262], Strauss 2010 [265], Svendsen 2019 [267], Torgalsbøen 2025 [270].

Eleven independent primary studies (N = 3531; [178,184,208,217,226,230,245,262,265,267,270]) reported long-term functional recovery proportions. As displayed in Figure 2, the random-effects pooled recovery rate was 35.4% (95% CI 22.6–49.4%), with very high heterogeneity (I^2^ = 98%, Q = 441.1, τ^2^ = 0.217) and a wide prediction interval (3–81%). The constituent estimates spanned this range in clinically interpretable ways: a 12-month trajectory analysis of 202 individuals assigned 35.7% to a recovery trajectory [245]; a multi-decade cohort found women achieving greater long-term improvement in psychotic activity than men at 7.5- and 20-year follow-up [208]; and an African synthesis reported a pooled positive-outcome rate of 44.2% [226].

#### 3.3.2. Symptomatic Remission

**Figure 3:**
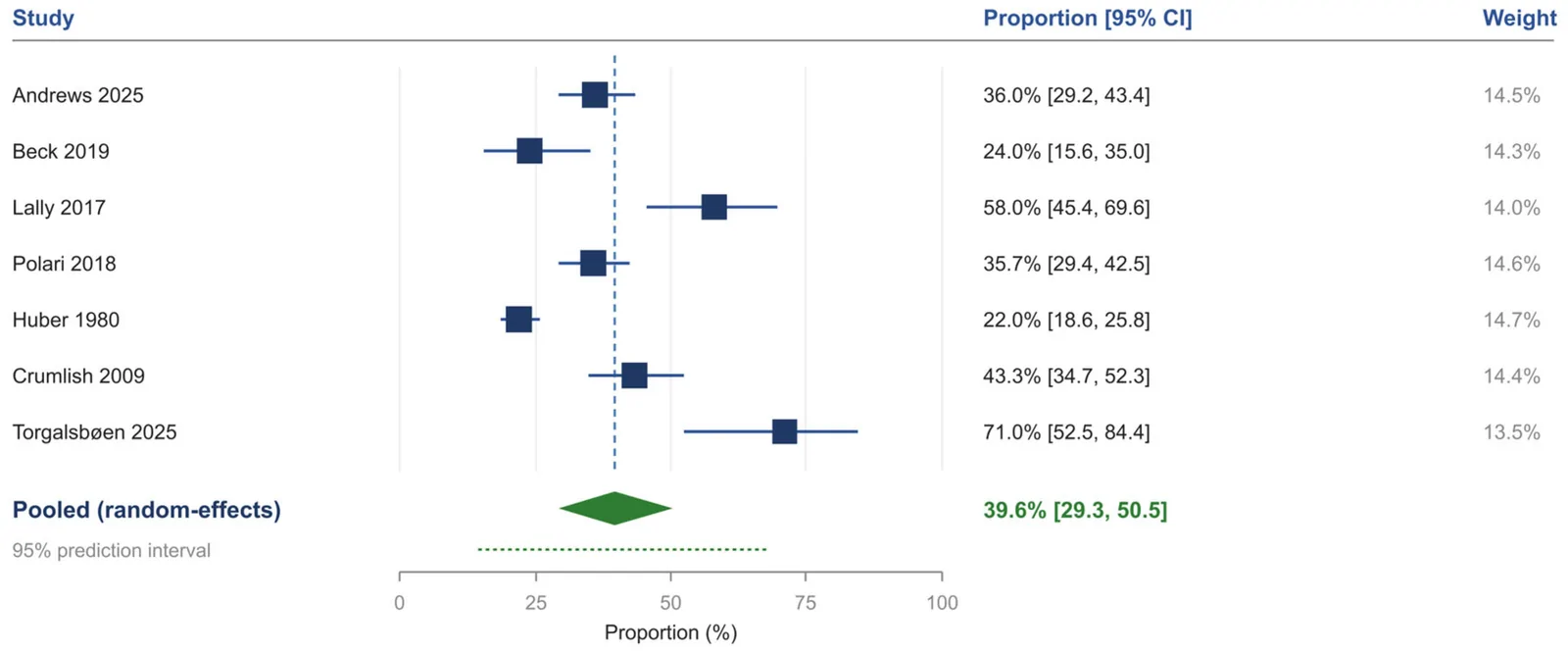
Random-effects forest plot of symptomatic-remission proportions (k = 7; N = 1153). Pooled Freeman–Tukey estimate 39.6% (95% CI 29.3–50.5%); I^2^ = 92%. Conventions as in Figure 2. Included studies: Andrews 2025 [174], Beck 2019 [178], Crumlish 2009 [196], Huber 1980 [217], Lally 2017 [230], Polari 2018 [245], Torgalsbøen 2025 [270].

Seven independent primary studies (N = 1153; [174,178,196,217,230,245,270]) reported symptomatic-remission proportions. The pooled remission rate (Figure 3) was 39.6% (95% CI 29.3–50.5%) (I^2^ = 92%, Q = 70.8; prediction interval 14–68%). Notably, in these independent primary pools the remission–recovery gap was modest (39.6% vs. 35.4%), in contrast to the larger separation reported elsewhere; that wider gap appears to be driven by high-remission meta-analytic sources, for example, a pooled remission estimate of 58% in a synthesis of 60 studies [230], which were deliberately excluded from the primary estimate to avoid double-counting and retained only in the sensitivity analysis (Section 3.3.4). Remission itself was frequently unstable: one cohort classified 17.2% of patients as being in stable remission, 57.8% in unstable remission, and 25.0% as unremitted, with the stable-remission group showing the lowest psychopathology [222].

#### 3.3.3. Trajectories and Prognostic Factors (Narrative)

Beyond the pooled rates, the cluster consistently described multi-class trajectories and a reproducible set of prognostic factors. Distinct negative-symptom trajectories, minimal-stable (59.6%), mild-stable (29.4%), and high-increasing (11.0%), were identified in a first-episode cohort, with poorer premorbid adjustment and greater baseline severity predicting the high-increasing class [191]. In clinical-high-risk samples, transitions accrued over extended follow-up (26%, 31%, and 35% at 3, 5, and longer intervals), underscoring the need for prolonged monitoring [178], while staging models documented that 36.9% of participants progressed from an attenuated to a more discrete syndrome, a transition associated with lower social and educational functioning [218]. Specific clinical markers carried prognostic weight: among high-risk individuals who converted, the great majority developed schizophrenia-spectrum rather than affective psychosis [203], and frequent insomnia was associated with an elevated risk of suicide attempts [231]. Long-term service-use data tempered the recovery picture, with roughly one in five patients never rehospitalized but excess mortality remaining substantial (24% died, including 9.9% by suicide) [177]. Synthesizing across studies, the directionally consistent favorable predictors were shorter duration of untreated psychosis, better premorbid adjustment, female sex, medication adherence, family involvement, and enrolment in early-intervention services, the last associated with patients being markedly more likely to gain employment and mainstream housing than those in standard care [272], whereas prominent baseline negative symptoms, substance-use comorbidity, social isolation, and poorer cognition predicted worse outcomes. These directions, summarized in Table 4, are reported qualitatively because the contributing studies did not provide comparably measured independent effect sizes. Functional trajectories were similarly heterogeneous: one cohort distinguished deteriorating-and-volatile (49%), persistent-impairment (16%), and recovering classes [219], early symptomatic remission predicted later functional recovery [173], and among clinical-high-risk youth, roughly 30% converted while 36% remitted, and 30% functionally recovered within two years [257].

#### 3.3.4. Sensitivity Analysis Results

Both proportion pools proved robust. For recovery, leave-one-out estimates ranged from 32.4% to 37.9%, and no single study carried more than 9.7% of the weight. Only the Hartung–Knapp specification coincided with the primary estimate (35.4%); the fixed-effect estimate was markedly lower (22.8%; 95% CI 21.4–24.2; Table S6b), an informative divergence indicating that a small number of large cohorts dominate under fixed-effect weighting and reinforcing the random-effects model as the appropriate primary specification. Adding reviews and meta-analyses changed the estimate negligibly (35.6%, k = 13). For remission, leave-one-out estimates ranged from 35.7% to 42.8%, and the review-inclusive estimate was 42.6% (k = 11). Supplementary Table S6a–c provide full diagnostics. Having established the quantitative course outcomes, the synthesis next turns to the interventions intended to improve them.

### 3.4. RQ2: Psychological and Combined Treatments [284,285,286,287,288,289,290,291,292,293,294,295,296,297,298,299,300,301,302,303,304,305,306,307,308,309,310,311,312,313,314] (Narrative)

Because fewer than five independent primary studies reported comparably measured long-term functional effect sizes for any single modality, RQ2 was synthesized narratively rather than pooled. The evidence nonetheless converged on a consistent, modality-spanning benefit. Cognitive-behavioral and combined approaches were associated with reduced transition and symptom burden, with CBT combined with antidepressant treatment linked to the lowest transition rate among intervention configurations in a high-risk sample [290], and psychoeducational family intervention yielding higher quality of life than control across the final treatment period [291]. Process-oriented psychotherapies showed durable gains: metacognitive narrative psychotherapy enhanced recovery and metacognition, maintained at two-year follow-up [312], and cognitive-behavioral social-skills training improved functioning and momentary affect [287]. Third wave and lifestyle approaches contributed broad secondary benefits: a mindfulness-based program produced medium-to-large effects on negative symptoms [288]; structured aerobic exercise was associated with reductions in depressive (≈32%) and anxiety (≈28%) symptoms [303]; and a multimodal lifestyle program reduced BMI and blood pressure while improving quality of life [286]. Effects were not uniformly positive. Integrated psychodynamic and supportive interventions showed only a non-significant trend in social functioning [311], and a lifestyle trial found no significant changes in weight, activity, or diet [294], reinforcing that long-term functional benefit depends on modality and implementation. Overall, effects clustered in the small-to-medium range, with combined and early-intervention configurations the most consistently beneficial; these are qualitative conclusions, not pooled estimates. The clinical stakes of effective long-term care were underscored by service-level gaps—approximately 40% of patients received no recent mental health treatment [306]—and by the elevated mortality of severe mental illness (hazard ratio ≈ 2.3) [284]; engagement with care was itself predicted by outcome expectations and self-efficacy [314].

### 3.5. RQ3: Cognitive Predictors of Functional Recovery [315,316,317,318,319,320,321,322,323,324,325,326,327,328,329,330,331,332,333,334,335,336,337,338,339,340,341,342,343,344,345,346,347,348,349,350,351,352,353,354,355,356,357,358,359,360,361,362] (Narrative)

RQ3 was likewise synthesized narratively. The evidence robustly indicated that cognition both predicts and constrains later functioning, while remaining clinically heterogeneous. Cognitive deficits were prevalent and partly enduring, neurocognitive dysfunction was documented in 46.8% of a first-episode sample at 12 months [351], and severe deficits were present in roughly half of patients after the acute stage [333], and intellectual trajectories diverged markedly, with about a quarter of patients showing stable low IQ and around 44% showing deteriorated IQ [344]. Importantly, first-episode cognitive performance improved relative to healthy comparators through approximately year 6, then stabilized, suggesting a window for remediation [362]. Decrements preceded illness onset (premorbid IQ effect size ≈ −0.43, with schizophrenia risk rising as IQ fell) [339], and working memory, attention/early perception, and other neurocognitive factors predicted functional outcome [352]. Predictive modeling integrating cognitive and clinical variables classified recovery status with approximately 76% accuracy [322] and supported employment cohorts translated cognitive capacity into vocational gains, with competitive employment rising from 10% at baseline to roughly 23% and 21% at one and two years [350]. Social cognition was comparatively stable over the first year and associated with concurrent functioning [336], and, together with motivation, emerged across studies as a stronger proximal predictor of real-world functioning than individual neurocognitive domains. The absence of comparably measured independent effect sizes precluded a defensible pooled correlation, so we report the cognition–functioning relationship qualitatively. Complementary findings showed that large, generalized cognitive deficits were robust across the lifespan [337], that treatment-associated cognitive improvement nonetheless occurred [320], and that neurocognitive course tracked functional outcome across psychotic and affective diagnoses [338].

### 3.6. RQ4: Psychosocial Rehabilitation and Community Integration [363,364,365,366,367,368,369,370,371,372,373,374,375,376,377,378,379,380,381,382,383,384,385,386,387,388,389,390,391,392,393,394,395,396,397,398,399,400,401,402] (Narrative)

RQ4 evidence was synthesized narratively. Integrated and adequately resourced rehabilitation models showed the broadest functional benefits: an integrated dual-disorder program improved five of six outcomes, including absence of psychiatric symptoms (45% → 70%), substance-use remission (8% → 61%), and independent housing (33% → 47%) [367], and greater service intensity and continuity in community-based rehabilitation were associated with better client outcomes [364]. Vocational integration responded to structured support; a work-oriented program increased employment participation by 15.3 percentage points per year versus 5 in controls [374], and inpatient rehabilitation services discharged over half of their patients within 18 months, reducing care costs [380]. Social functioning improved to a medium magnitude over time (d ≈ 0.60), with larger gains when illness duration was under 5 years [400], echoing the early-phase advantage seen in RQ1. Continuity of care mattered at transitions: timely post-discharge outpatient follow-up (within 30 days) was associated with modestly lower readmission risk [386]. These objective functional gains were nonetheless conditioned by broader social determinants, socioeconomic status, substance-use comorbidity, trauma exposure, family environment, and caregiver burden, and no employment-rate pool is reported, as the available estimates were too few and heterogeneous to pool defensibly. Single-cohort figures illustrated the vocational gap directly, only 32.5% of one first-episode sample were employed, and 21.3% were in a relationship at follow-up [171], and the two-year transition rate in an at-risk service was 16.9% [366]; caregiver burden was substantial, with depression reported in 12–40% of caregivers [392].

### 3.7. RQ5: Personal Recovery and Quality of Life [403,404,405,406,407,408,409,410,411,412,413,414,415,416,417,418,419,420,421,422,423,424,425,426,427,428,429,430,431,432,433,434,435,436,437,438,439,440,441,442,443,444,445,446,447,448,449,450,451] (Narrative)

RQ5 was synthesized narratively, and its central finding was the partial independence of personal from clinical recovery. Subjective well-being and quality of life followed distinct multi-class trajectories rather than a uniform course: four well-being clusters (stable low 33%, stable moderate 31%, stable high 16%, and an improving subgroup) were identified in one cohort [422], and four subjective-quality-of-life classes were distinguished at five-year follow-up [409]. Quality of life generally improved over the multi-year follow-up period, though unevenly across domains [417], and was more strongly associated with service satisfaction for subjective than for objective indices (r ≈ 0.30) [432]. Critically, personal recovery predicted well-being independently of clinical and functional status [404], and the quality of interpersonal relationships prospectively predicted change in personal-recovery scores [426], while baseline stigma resistance forecast later identity affirmation and valued living [405]. Dual-factor models that treat well-being and psychopathology as distinct dimensions received support [428], and forensic and long-stay cohorts showed that most could transition to less restrictive settings with symptomatic improvement over the follow-up period [445]. Where gains occurred, they could be durable: eudaimonic well-being rose and was maintained at six months [410]; subjective quality of life improved across physical, psychological, social, and environmental domains [443]; and broader social involvement supported self-management and well-being [434]. Because contributing studies used heterogeneous instruments and reported few comparable effect sizes, no pooled estimate is presented.

### 3.8. RQ6: Mediators and Moderators of Recovery [452,453,454,455,456,457,458,459,460,461,462,463,464,465,466,467,468,469,470,471,472,473,474,475,476,477,478,479,480,481,482,483,484,485,486,487,488,489,490,491,492,493,494,495,496,497,498,499,500,501,502,503,504,505,506,507,508,509,510,511,512,513,514,515,516,517,518,519,520,521,522,523,524,525,526,527,528,529,530,531,532,533,534,535,536,537,538]

**Figure 4:**
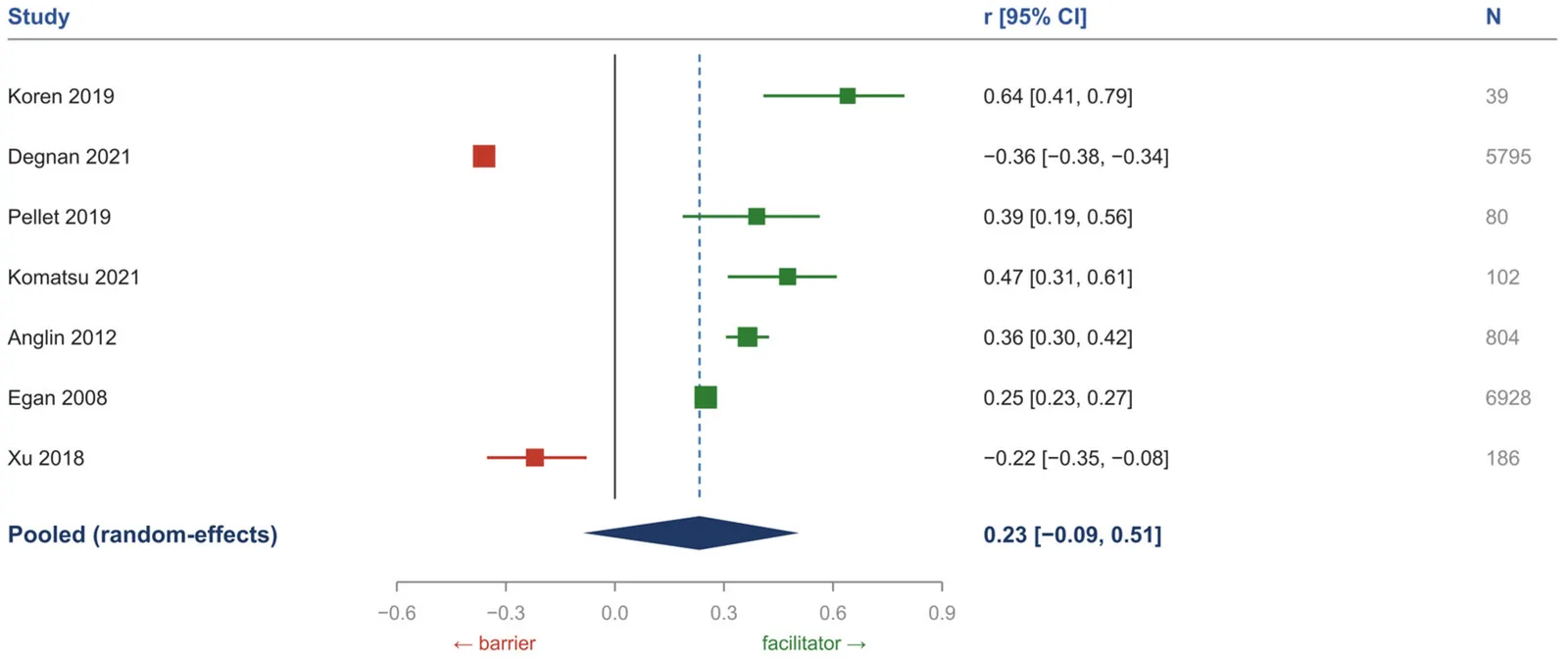
A random-effects forest plot of mediator–outcome correlations (k = 7; N = 13,934). The green markers denote facilita-tive (positive) associations; the red markers denote barrier (negative) associations. The diamond represents the pooled Fisher-z estimate, r = 0.23 (95% CI −0.09 to 0.51). The opposing directions of association and I^2^ ≈ 100% ex-plain the wide interval spanning the null. Included studies: Anglin 2012 [453], Degnan 2021 [462], Egan 2008 [466], Komatsu 2021 [485], Koren 2019 [487], Pellet 2019 [515], Xu 2018 [537].

Seven independent primary studies (N = 13,934; refs [453,462,466,485,487,515,537]) provided correlations between psychological mediators and long-term functional outcomes. As Figure 4 makes visually explicit, the random-effects pooled association was r = 0.23 (95% CI, −0.09–0.51), with essentially complete heterogeneity (I^2^ ≈ 100%) and a confidence interval that spanned the null because the constituent constructs point in opposite directions. Facilitators (positive mental health, social support, self-efficacy, hope) yielded positive correlations, whereas barriers (internalized stigma, substance-use comorbidity, longer duration of untreated psychosis, persistent negative symptoms) yielded negative ones, as organized in Table 5. The wider mediator literature situated these associations within developmental and biological context: cognitive decline was detectable up to 14 years before psychosis onset (≈0.35 IQ points/year) [482]; elevated childhood fasting insulin from age 9 predicted later psychosis [516]; childhood maltreatment and interpersonal violence were linked to the majority of adverse psychiatric and psychosocial outcomes [458]; baseline negative symptoms and disorganized thought predicted worse functioning over time [533]; and excess mortality, though declining since the 1970s, remained elevated [492]. Self-stigma was common, reported at high levels by roughly a third of patients [464]. Encouragingly, one cohort observed 45.5% full recovery over six years, with the greatest improvement during the first two years [530], again localizing change to the early phase. Rated very low certainty, the pooled estimate should be interpreted as indicating the presence, rather than the pre-cise magnitude, of mediator effects. Biological findings reinforce this developmental picture: about three-quarters of biomarker studies reported abnormalities consistent with accelerated aging [511], psychological distress predicted greater emergency-service use [507], and gene-by-sex interactions modulated longitudinal functioning [477]. To address the structural (rather than merely empirical) heterogeneity of this omnibus estimate, we conducted a pre-planned, direction-harmonized split. The five facilitator associations (positive mental health, social support, self-efficacy, service engagement, and metacognitive/social-cognitive capacity; Anglin 2012 [453], Egan 2008 [466], Komatsu 2021 [485], Koren 2019 [487], Pellet 2019 [515]) met the pooling threshold (k = 5) and yielded a positive, interpretable pooled correlation, r = 0.38 (95% CI 0.28–0.48; I^2^ = 85%; N = 7953), with a confidence interval that excludes the null. The two barrier associations (internalized stigma and low insight; Degnan 2021 [462], r = −0.36, N = 5795; Xu 2018 [537], r = −0.22, N = 186) fell below the pooling threshold (k = 2) and are reported narratively; both showed a consistent negative association with functional outcome. Thus, the near-null omnibus estimate (r = 0.23) reflects the cancelation of oppositely signed facilitator and barrier effects rather than an absence of association, and the direction-specific estimates are the interpretively meaningful quantities. Within the facilitator pool, the largest cohort (Egan 2008 [466], N = 6928 of a pooled N = 7953; Table S6c) dominates the weighting, so the facilitator estimate is best read as anchored by that study. 

### 3.9. Cross-Cutting Themes

Read together, the pooled and narrative findings converge on three themes. First, course and outcome are multidimensional: recovery (35.4%) and remission (39.6%) are related but distinct, and the multi-class trajectories recurring across RQ1 and RQ5 [191,222,409,422] confirm that symptomatic relief does not guarantee functional or personal recovery. Second, modifiable psychological and social factors, including cognition [344,362], social support and self-efficacy [270,431], and stigma [405,464], recur across clusters as levers on functional outcome, even where their effects could not be precisely pooled, pointing to high-yield intervention targets. Third, the early phase of illness recurs as a period of disproportionate prognostic importance: shorter duration of untreated psychosis, early-intervention enrolment [272], early social-functioning gains [400], and the concentration of recovery within the first two years [530] all converge on the need for timely, coordinated care. These are integrative observations grounded in the synthesized evidence, not additional quantitative estimates.

### 3.10. Risk of Bias and Certainty of Evidence

**Figure 5:**
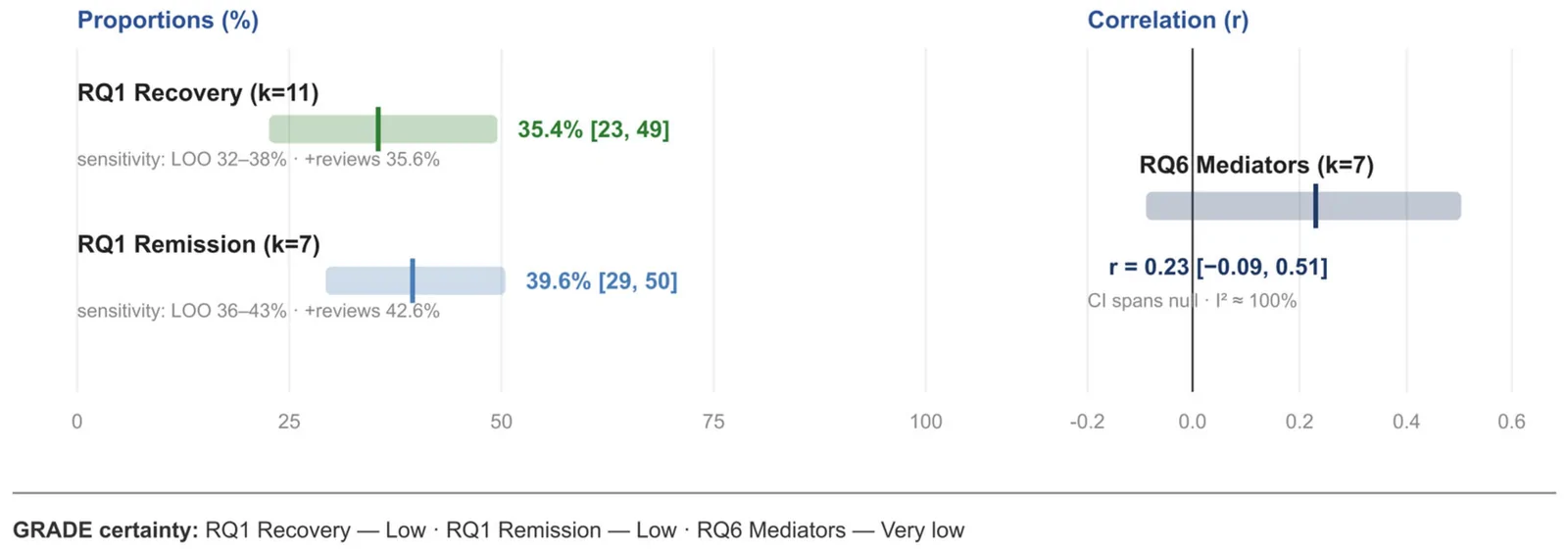
A summary of the three pooled estimates with sensitivity bounds and GRADE certainty. The solid lines are pooled point estimates; the shaded bands are 95% CIs. Both proportion pools were robust under leave-one-out and review-inclusive sensitivity analyses; the mediator association is imprecise and spans the null. Certainty: recovery and remission Low; mediators Very low.

We appraised risk of bias for the pooled studies using design-appropriate tools and finalized it through dual independent full-text adjudication, as reported in Supplementary Table S4. As summarized in Figure 5 and Table 6, GRADE certainty was rated low for both proportion pools, downgraded for very high inconsistency and—for re-mission—additionally for imprecision from a small evidence base. The mediator pool was rated very low, down-graded further for indirectness and a confidence interval spanning the null. The narratively synthesized questions (RQ2–RQ5) were not assigned pooled certainty ratings. The finalized risk-of-bias ratings (Table S4) informed the GRADE risk-of-bias domain: primary studies were rated as having some concerns, and the six meta-analyses/reviews used as secondary sources were rated as high risk, which contributed to the certainty downgrading.

### 3.11. Summary of Key Findings

In this consolidated synthesis of 368 included studies [171,172,173,174,175,176,177,178,179,180,181,182,183,184,185,186,187,188,189,190,191,192,193,194,195,196,197,198,199,200,201,202,203,204,205,206,207,208,209,210,211,212,213,214,215,216,217,218,219,220,221,222,223,224,225,226,227,228,229,230,231,232,233,234,235,236,237,238,239,240,241,242,243,244,245,246,247,248,249,250,251,252,253,254,255,256,257,258,259,260,261,262,263,264,265,266,267,268,269,270,271,272,273,274,275,276,277,278,279,280,281,282,283,284,285,286,287,288,289,290,291,292,293,294,295,296,297,298,299,300,301,302,303,304,305,306,307,308,309,310,311,312,313,314,315,316,317,318,319,320,321,322,323,324,325,326,327,328,329,330,331,332,333,334,335,336,337,338,339,340,341,342,343,344,345,346,347,348,349,350,351,352,353,354,355,356,357,358,359,360,361,362,363,364,365,366,367,368,369,370,371,372,373,374,375,376,377,378,379,380,381,382,383,384,385,386,387,388,389,390,391,392,393,394,395,396,397,398,399,400,401,402,403,404,405,406,407,408,409,410,411,412,413,414,415,416,417,418,419,420,421,422,423,424,425,426,427,428,429,430,431,432,433,434,435,436,437,438,439,440,441,442,443,444,445,446,447,448,449,450,451,452,453,454,455,456,457,458,459,460,461,462,463,464,465,466,467,468,469,470,471,472,473,474,475,476,477,478,479,480,481,482,483,484,485,486,487,488,489,490,491,492,493,494,495,496,497,498,499,500,501,502,503,504,505,506,507,508,509,510,511,512,513,514,515,516,517,518,519,520,521,522,523,524,525,526,527,528,529,530,531,532,533,534,535,536,537,538], long-term functional recovery was achieved by an estimated 35.4% (95% CI 22.6–49.4) and symptomatic remission by 39.6% (95% CI 29.3–50.5) of individuals with schizophrenia-spectrum disorders, both robust across leave-one-out, alternative-specification, and review-inclusive sensitivity analyses but characterized by very high heterogeneity. Psychological mediators showed a positive but imprecise pooled association with functional outcome (r = 0.23; 95% CI –0.09 to 0.51). Narratively, structured psychosocial and combined interventions [286,290,303,312] and integrated rehabilitation [367,400] were associated with small-to-medium functional gains; cognition, particularly social cognition and motivation, predicted later functioning [339,344,362]; and personal recovery followed a course partly independent of clinical status [404,405,422]. Across domains, the early illness phase and a set of modifiable psychosocial factors emerged as the most consistent levers on long-term recovery. Section 4 interprets these findings.

## 4. Discussion

**Figure 6:**
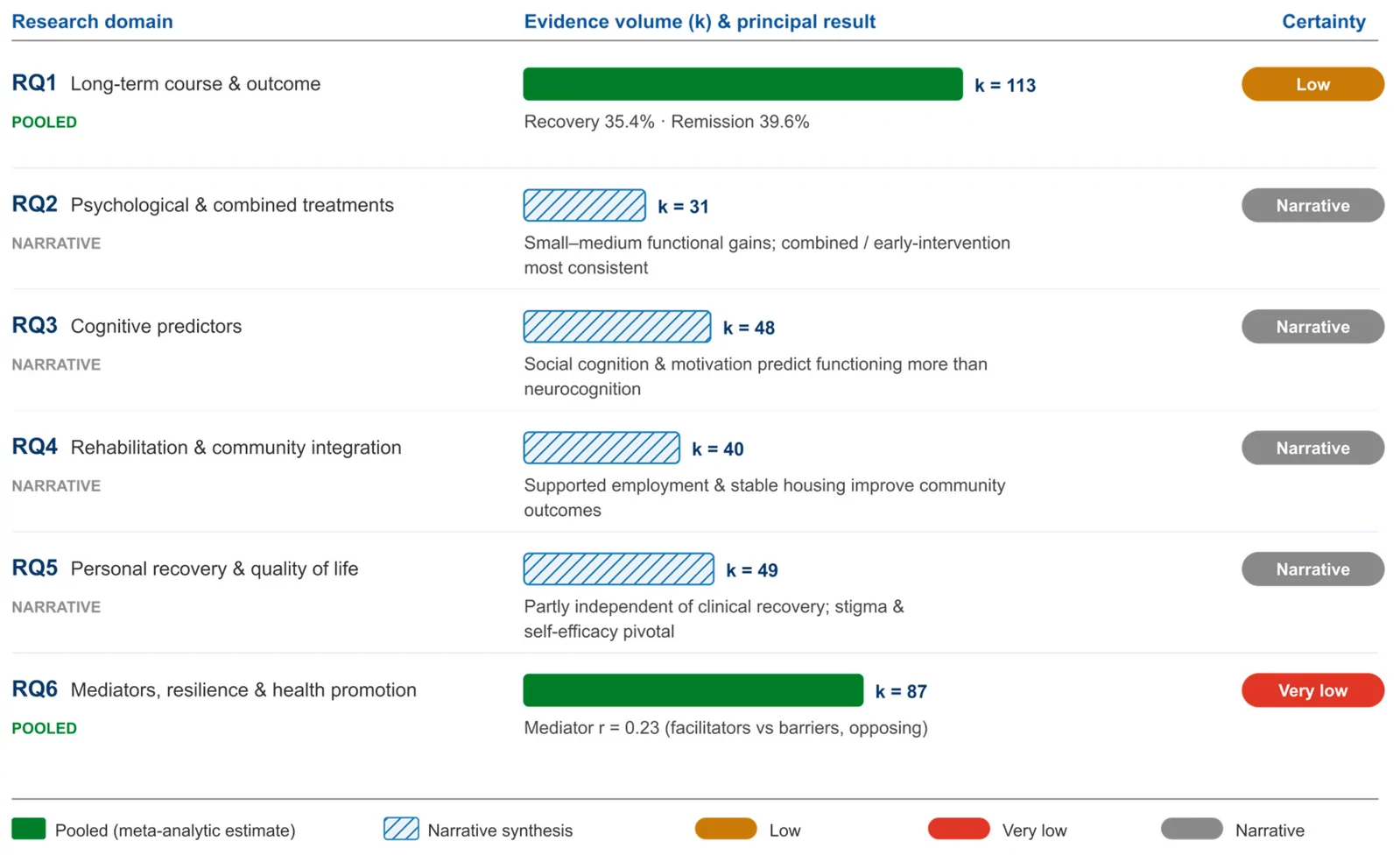
An evidence map across the six research domains. Each row represents one research question (RQ1–RQ6); the bar length is proportional to the number of contributing primary studies (k). The solid bars denote domains synthesized quantitatively (meta-analytic pools); the hatched bars denote domains synthesized narratively because comparable effect sizes were too few to pool (k ≤ 3). For each domain, the principal result is shown, along with pooled point estimates for RQ1 (recovery, remission) and RQ6 (mediator r), direction-of-evidence statements for RQ2–RQ5, and the GRADE certainty rating. All quantitative estimates carried very high heterogeneity (I^2^ = 92–100%). RQ, research question.

**Figure 7:**
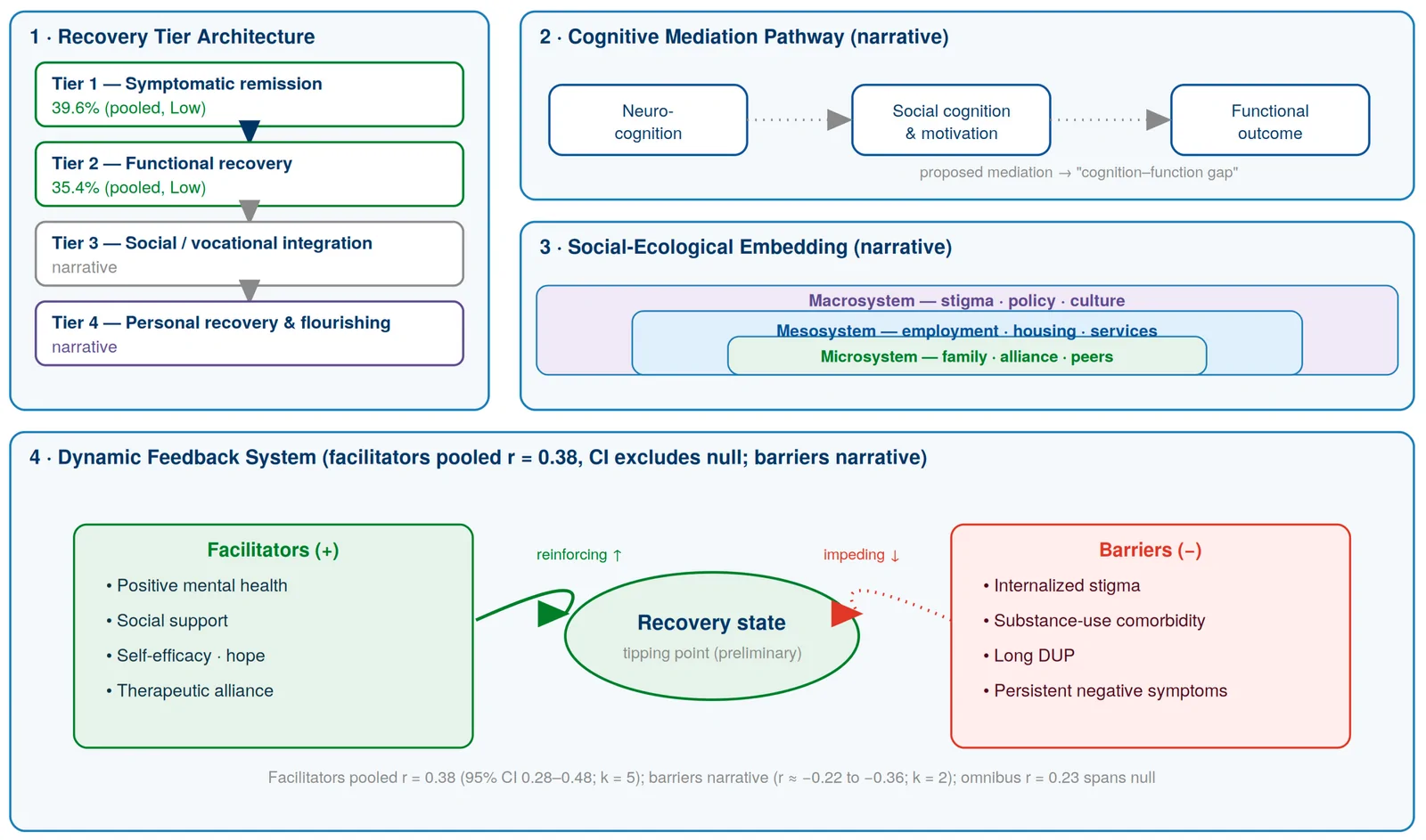
The Multi-Pathway Dynamic Recovery Model (MPDR). An integrative framework synthesizing the six clusters into four modules (Recovery Tier Architecture; Cognitive Mediation Pathway; Social-Ecological Embedding; Dynamic Feedback System), with the early-intervention window highlighted as the period of maximum leverage. The solid arrows denote pathways anchored to pooled estimates (recovery 35.4%; remission 39.6%; mediator r = 0.23); the dashed arrows denote proposed or narratively supported mechanisms requiring further investigation.

This systematic review synthesized 368 longitudinal studies spanning six research domains, applying meta-analysis where the evidence permitted and structured narrative synthesis elsewhere. Figure 6 maps this evidence base, placing side by side the volume of contributing studies, the principal result, and the certainty of evidence for each domain. Two patterns stand out. First, the domains richest in studies, long-term course and outcome (k = 113) and mediators and resilience (k = 87), were precisely those that supported quantitative pooling, yielding a long-term recovery rate of 35.4%, a remission rate of 39.6%, and a weak, imprecise mediator–outcome association (r = 0.23). Second, the intervention, cognitive, rehabilitation, and personal-recovery domains (RQ2–RQ5) provided substantial but too few and too heterogeneous effect sizes to pool, so they are summarized narratively. Certainty was low for the pooled recovery and remission estimates and very low for the mediator pool, reflecting the very high heterogeneity (I^2^ = 92–100%) observed across all quantitative syntheses. The following sections interpret each domain in turn, then integrate them into the proposed Multi-Pathway Dynamic Recovery Model (Figure 7).

### 4.1. Summary and Interpretation of Principal Findings

#### 4.1.1. Long-Term Course and Outcome (RQ1)

The pooled long-term functional recovery rate of 35.4% (95% CI 22.6–49.4%; k = 11, N = 3531) challenges the Kraepelinian assumption of inevitable deterioration and indicates that roughly one in three individuals achieves sustained functional recovery. The estimate is consistent with the first-episode meta-analysis of Lally and colleagues (≈38%) [230], retained here for sensitivity rather than the primary pool, and with an African synthesis reporting a 44.2% positive-outcome rate [226]. The companion remission estimate was 39.6% (95% CI 29.3–50.5%; k = 7). Notably, in these independent primary pools, the remission–recovery gap was narrow, in contrast to the larger separation implied by symptom-based meta-analytic sources; this convergence suggests that, once non-independent and high-remission syntheses are excluded, the empirical distance between symptomatic and functional recovery is smaller and more uncertain than often assumed. Recovery should still be understood as conceptually more demanding than remission, but the present data do not support a steep, precisely quantified tiered gradient. Subgroup heterogeneity was stark: deficit-syndrome patients recovered at far lower rates (13%) than non-deficit patients (63%) [265], persistent hallucinations across 20 years predicted poorer recovery and work attainment [205], and first-episode cohorts showed pronounced employment and relationship deficits [171].

The very high heterogeneity (I^2^ = 98% for recovery, 92% for remission) is a substantive finding rather than a methodological artifact, reflecting genuine variation in recovery definitions, follow-up durations, diagnostic boundaries, and healthcare and cultural contexts; the wide prediction intervals (recovery 3–81%) make explicit that any single setting may observe markedly higher or lower rates. The narrative evidence on trajectories reinforced this picture, repeatedly resolving the population into distinct latent classes, for example, minimal-stable, mild-stable, and high-increasing negative-symptom paths [191], and stable, unstable, and unremitted course types [222]. Across studies, the early illness phase emerged as prognostically pivotal: enrolment in early-intervention services was associated with substantially higher rates of employment and stable housing [272], the greatest gains tended to occur within the first two years [530], and social-functioning improvement was larger where illness duration was under five years [400]. This convergence supports frontloading comprehensive psychosocial intervention rather than escalating it only after treatment failure.

#### 4.1.2. Psychological and Combined Treatments (RQ2)

Because fewer than five independent primary studies reported comparably measured long-term functional effect sizes for any single modality, RQ2 was synthesized narratively, and no pooled effect sizes are reported. The qualitative pattern, however, was coherent: structured psychosocial interventions were generally associated with functional and symptomatic benefit, and combined or integrated configurations appeared more consistently helpful than single-component approaches. Process-oriented psychotherapies showed durable gains, metacognitive narrative psychotherapy enhanced recovery and metacognition with maintenance at two years [312], and cognitive-behavioral social-skills training improved functioning and affect [287], while mindfulness-based [288], psychoeducational family [291], and lifestyle/exercise programs [286,303] contributed additional, often secondary, benefits. Effects were not uniformly positive (e.g., a lifestyle trial found no change in weight or activity [294]), reinforcing that long-term functional benefit depends on modality and implementation. The apparent advantage of multicomponent packages is consistent with the principle that recovery is multidimensional and therefore best served by interventions that simultaneously address symptoms, cognition, social functioning, employment, and family context; this remains a narrative inference rather than a quantified comparison. Longer-range observational work situated these interventions within a broader risk picture, non-affective psychosis carried elevated long-term dementia risk [305], and sleep disturbance prospectively predicted positive symptoms in ultra-high-risk youth [302], identifying additional, potentially modifiable intervention targets.

#### 4.1.3. Cognitive Predictors of Functional Recovery (RQ3)

RQ3 was likewise synthesized narratively. The evidence was robust and consistent in indicating that cognition both predicts and constrains later functioning: neurocognitive dysfunction was common and partly enduring [333,351], intellectual trajectories diverged among patients [344], and premorbid intellectual decrements preceded illness onset [339]. Importantly, first-episode cognitive performance improved relative to comparators up to roughly year six before stabilizing, suggesting a remediation window [362], and cognitive capacity translated into vocational gains in supported employment cohorts [350]. Through studies, social cognition and motivation recurred as comparatively strong proximal predictors of real-world functioning, consistent with a mediation account in which neurocognition exerts its effects indirectly through social-cognitive processes and goal-directed motivation. Because comparably measured independent effect sizes were unavailable, this account is advanced qualitatively; it nonetheless implies that remediation programs prioritizing social cognition and motivational enhancement may yield greater functional transfer than those targeting basic neurocognition alone. Consistent with this mediation account, social cognition explained functional-outcome variance beyond neurocognition [332], and cognitive deficits predicted both conversion and non-remission [341].

#### 4.1.4. Social Ecology of Recovery (RQ4)

The rehabilitation evidence, synthesized narratively, portrayed recovery as fundamentally socially embedded. Integrated and adequately resourced models produced the broadest benefits; an integrated dual-disorder program improved five of six outcomes, including independent housing [367], and greater service intensity and continuity were associated with better outcomes [364], while structured vocational support increased employment participation [374] and inpatient rehabilitation discharged the majority of patients within 18 months [380]. Functional gains were nonetheless conditioned by social determinants (socioeconomic status, substance-use comorbidity, trauma, family environment, caregiver burden) and by continuity of care at transitions, where timely post-discharge follow-up reduced readmission risk [386]. That even integrated programs can improve symptoms and functioning yet leave housing relatively unchanged underscores the limits of individually oriented interventions when structural determinants, including housing supply, income support, transportation, remain unaddressed, motivating a social-ecological view of recovery. Psychological resources also shaped outcomes: an internal locus of control was associated with more recovery periods over 15 years [371], and emotion processing predicted work functioning and independent living [379].

#### 4.1.5. Personal-Recovery Trajectories (RQ5)

Perhaps the most theoretically consequential narrative finding was the partial independence of personal from clinical recovery. Personal recovery predicted well-being independently of symptomatic and functional status [404]; the quality of interpersonal relationships prospectively predicted change in personal-recovery scores [426]; baseline stigma resistance forecast later identity affirmation and valued living [405]; subjective well-being and quality of life followed distinct multi-class trajectories [409,422]. These observations challenge the assumption that symptom reduction will automatically cascade into subjective recovery and are well captured by narrative-identity and dual-factor perspectives, in which personal recovery involves reconstruction of self-concept and meaning over longer timescales than behavior-based functional change. The practical corollary is that personal-recovery goals require relational and experiential approaches, monitored with dedicated instruments, distinct from clinical targets. The reciprocity between role functioning and subjective recovery was also evident; employment was associated with reduced service use and improved self-esteem [427], while lower depression and higher self-efficacy and social support predicted better quality of life [435].

#### 4.1.6. Mediating and Moderating Mechanisms (RQ6)

The mediator analysis was pooled but yielded a weak and highly uncertain estimate: the random-effects association between psychological mediators and long-term functional outcome was r = 0.23 (95% CI −0.09–0.51; k = 7, N = 13,934), with a confidence interval spanning the null and near-complete heterogeneity (I^2^ ≈ 100%). This imprecision is not evidence of absent effects but a direct consequence of aggregating constructs that act in opposite directions: facilitators such as positive mental health, social support, self-efficacy, and hope correlate positively with outcome, whereas barriers such as internalized stigma, substance-use comorbidity, longer duration of untreated psychosis, and persistent negative symptoms correlate negatively. Self-stigma was common, reported at high levels by roughly a third of patients [464]. Developmental and biological mediators situated these associations in a longer arc, with cognitive decline detectable years before onset [482], ear-ly metabolic markers predicting later psychosis [516], and childhood adversity linked to multiple adverse outcomes [458]. The appropriate inference is that mediator effects are present and directionally interpretable, but the current data cannot assign a precise common magnitude; the pooled estimate is rated with very low certainty. Two further findings warrant note: insight and explanatory models were associated with markedly higher remission [481], and resilience acted as a protective factor with bidirectional effects on well-being [493]; one long-term cohort even reported better recovery among patients off antipsychotics after the first two years [476], a provocative observation requiring cautious, confounding-aware interpretation.

### 4.2. Toward an Integrative Framework: The Multi-Pathway Dynamic Recovery Model (MPDR)

The convergence of pooled and narrative findings motivates an integrative framework that can accommodate the multidimensional, dynamic, and context-dependent nature of recovery. Existing models including Liberman and Kopelowicz’s operational criteria, Leamy et al.’s CHIME framework, and the WHO’s International Classification of Functioning each capture important dimensions but do not jointly integrate cognitive mediation, social ecology, temporal dynamics, and feedback mechanisms. We therefore propose the Multi-Pathway Dynamic Recovery Model (MPDR) as an empirically informed theoretical framework. We emphasize at the outset that the MPDR is a hypothesis-generating synthesis: only two of its anchors, the recovery and remission proportions, rest on pooled estimates (of low certainty), one further anchor (the mediator association) is pooled at very low certainty, and the remaining pathways are grounded in narrative evidence. The model is therefore offered for testing, not as a validated causal structure.

#### 4.2.1. Core Principles of the MPDR

The MPDR rests on five core principles derived from the present synthesis:

Principle 1—Multi-Domain Recovery Architecture. Recovery is conceptually ordered, from symptomatic remission (pooled 39.6%) through functional recovery (pooled 35.4%) to broader social–vocational integration and personal recovery (supported narratively). The present data place remission and functional recovery closer together than earlier tiered accounts implied, so the architecture is best read as a conceptual ordering of increasingly demanding criteria rather than a steep empirical gradient.

Principle 2—Cognitive Bottleneck and Social-Cognitive Mediation. Cognition appears to act as a rate-limiting factor, with its influence on functioning mediated by social cognition and motivation. This pathway is supported by narrative evidence (RQ3) rather than a pooled correlation and is proposed to explain the commonly observed gap between neurocognitive remediation gains and functional improvement.

Principle 3—Temporal Dynamics and the Window of Opportunity. The early illness phase (first 2–5 years) recurs across clusters as the period of maximum leverage, via early-intervention enrolment [272], concentration of gains in the first two years [530], and larger effects at shorter illness duration [400], motivating frontloaded, declining-intensity care.

Principle 4—Social-Ecological Embedding. Individual-level interventions operate within microsystem (family, alliance, peer support), mesosystem (employment, housing, service integration), and macrosystem (stigma, policy, culture) layers; the narrative superiority of integrated, multilayer models [367] over single-component approaches is consistent with this principle.

Principle 5—Feedback Loops and Tipping Points. Recovery may be sustained through self-reinforcing positive loops (hope, social support, self-efficacy) and impeded by negative loops (stigma, isolation, substance use). Given the weak, heterogeneous pooled mediator association (r = 0.23, CI spanning null), the proposed recovery “tipping point” is preliminary and awaits longitudinal, within-person testing.

#### 4.2.2. Structural Components of the Framework

Figure 7 illustrates the MPDR schematically, comprising four interconnected modules: a Recovery Tier Architecture (remission → functional recovery → social integration → personal recovery), a Cognitive Mediation Pathway (neurocognition → social cognition/motivation → functional outcome), a Social-Ecological Embedding layer (micro-, meso-, and macrosystem), and a Dynamic Feedback System (facilitator and barrier loops). The temporal dimension runs horizontally, with the early-intervention window marked as the period of maximum leverage. Consistent with the evidentiary status above, only the recovery and remission tiers and the mediator association are anchored to pooled estimates; all other arrows denote proposed or narratively supported pathways requiring confirmation (Table 7).

#### 4.2.3. Distinguishing the MPDR from Existing Frameworks

The MPDR is intended to complement, not supplant, existing models. Relative to the CHIME framework, which identifies recovery processes without specifying their interrelations, the MPDR proposes directional pathways and labels each by evidentiary status, enabling falsifiable testing. Relative to operational recovery criteria that treat recovery as a binary endpoint, it models recovery as a tiered, dynamic process with feedback. Relative to neurocognitive models positing cognition as a direct predictor, it specifies a mediated pathway (neurocognition → social cognition/motivation → functional outcome) intended to account for the cognition–function gap. These advances are conceptual; their empirical adjudication depends on the future studies outlined in Section 4.5.

### 4.3. Clinical and Policy Implications

Interpreted with appropriate caution, the synthesis supports several practice and policy directions. First, the convergence of evidence on the early illness window favors frontloaded, multicomponent psychosocial care, immediate access to cognitive-behavioral therapy, family intervention, supported employment, and cognitive remediation alongside pharmacotherapy, rather than stepwise escalation after treatment failure. Second, the narrative salience of social cognition and motivation as proximal predictors suggests that cognitive remediation should incorporate social-cognitive training and motivational enhancement, not basic neurocognitive drill alone. Third, the prominence of internalized stigma as a barrier (RQ6), together with its high prevalence [464], identifies anti-stigma work, narrative-identity work, peer support, and self-assertiveness training as a plausibly high-value component to embed within standard packages; we frame this as a promising target rather than a quantified large effect, given the heterogeneity of the mediator evidence. Fourth, the partial independence of personal and clinical recovery (RQ5) argues for dual-track planning that sets and monitors both symptomatic and personal-recovery goals with dedicated instruments. Fifth, the persistence of housing and structural gaps despite clinical gains (RQ4) indicates that recovery-oriented services must be complemented by housing, income, and anti-discrimination policy. Finally, the higher recovery rates reported in some lower-resource settings [226] caution against assuming the universal optimality of Western service models and support culturally adapted, family- and community-oriented approaches. Three caveats frame what follows. First, the pooled rates are broad benchmarks rather than precise population estimates, and vary substantially across healthcare systems, countries, and treatment eras. Second, given the very high heterogeneity (I^2^ = 92–98%) and low-to-very-low certainty, these estimates should anchor expectations rather than define thresholds. Third, the recommendations concerning social cognition and stigma derive largely from convergent narrative evidence rather than high-certainty pooled effects and are offered as directions for evaluation rather than established standards.

### 4.4. Limitations

Several limitations of this review should be acknowledged when interpreting the findings; they are organized into methodological, analytic, and conceptual domains.

#### 4.4.1. Design and Scope of the Synthesis

The most important limitation is evidentiary density. Only the long-term-course outcomes and the mediator association met the pre-specified threshold for pooling; the intervention, cognitive-prediction, rehabilitation, and personal-recovery questions are supported by narrative synthesis and should not be read as quantified effects. The quantitative pools were themselves small (k = 7–11), and the mediator pool was weak and heterogeneous (r = 0.23, CI spanning null), warranting only very low certainty. Cross-cluster integration and the MPDR framework are interpretive proposals that extend beyond the pooled data.

#### 4.4.2. Corpus Construction and Extraction

The corpus was assembled by consolidating and deduplicating two independently compiled extraction databases. Although this maximized coverage, and one residual duplicate was identified and removed, much of the extraction was conducted at the abstract level, which constrained the availability of structured effect-size data and necessitated the hybrid design. The finalized risk-of-bias ratings (Table S4) informed the GRADE risk-of-bias domain: primary studies were rated as having some concerns, and the six meta-analyses/reviews used as secondary sources were rated as high risk, which contributed to the downgrading of certainty.

#### 4.4.3. Evidence-Based

The predominance of observational longitudinal designs limits causal inference, and differential attrition in long-term psychosis research may bias upward estimates of recovery and remission; therefore, the pooled rates of 35.4% and 39.6% are best read as approximations bounded by wide prediction intervals. The extreme heterogeneity (I^2^ = 92–98%) reflects, in part, the absence of a universally accepted operational definition of recovery, which complicates aggregation and renders publication-bias diagnostics uninterpretable at the available pool sizes. The evidence base is geographically concentrated in high-income settings and may not generalize to the lower- and middle-income contexts where most people with psychotic disorders live, nor to forensic, intellectually disabled, or homeless populations. Finally, over the 1980–2025 period, evolving diagnostic criteria and service models mean that older cohorts may not reflect current practice.

### 4.5. Future Research Directions

The findings and limitations point to several priorities, organized around testing and strengthening the framework. First, the cognitive-mediation hypothesis can be tested in head-to-head randomized trials comparing social-cognitive and motivational training with traditional neurocognitive remediation, using functional outcome as the primary endpoint and neurocognitive change as a secondary mediator [327,332,341]. Second, the recovery tipping-point hypothesis calls for longitudinal designs with frequent repeated measures (e.g., ecological momentary assessment) and change-point or regime-switching models to detect nonlinear transitions—evidence that would directly inform decisions about treatment duration [219,422]. Third, sequential mediation models in large cohorts can examine the proposed tier ordering to determine whether remission is a necessary precondition for functional recovery or whether alternative pathways exist [173,230].

Methodologically, the field requires consensus operational definitions of recovery, ideally established through a Delphi process involving people with lived experience, to reduce the definitional heterogeneity that dominated this synthesis [230,270]; active follow-up and principled handling of missing data to mitigate attrition bias; digital phenotyping and ecological momentary assessment to capture recovery micro-processes; and individual-participant-data meta-analysis across longitudinal cohorts to resolve moderator questions (e.g., by sex, illness duration, or setting) that aggregate data cannot [341,343]. Substantively, low- and middle-income settings, forensic and late-onset populations, and digital or technology-assisted interventions remain under-studied and are priorities for rigorous longitudinal evaluation [226,303]. Dismantling and mechanism studies, randomized designs that vary the components of multicomponent packages, and experimental manipulation of candidate mediators such as motivation and stigma are needed to identify active ingredients and establish directionality [462,464]. Finally, formal cost-effectiveness and implementation-science studies are required to translate efficacy into routine, resource-constrained services, given the substantial lifetime costs of schizophrenia and the gap between trial efficacy and real-world effectiveness [427,435].

In sum, this review provides a quantitatively anchored account of long-term course (recovery 35.4%; remission 39.6%) and a transparent, narratively grounded synthesis of the interventions, cognitive predictors, social determinants, personal-recovery processes, and mediators that shape it. The MPDR framework organizes this evidence into testable propositions and, together with the priorities above, offers a roadmap for moving from heterogeneous description toward a mechanistic, actionable understanding of recovery in schizophrenia-spectrum disorders.

## 5. Conclusions

This systematic review and meta-analysis of 368 longitudinal studies provides a comprehensive, methodologically transparent synthesis of the evidence on psychosocial interventions and long-term functional recovery in schizophrenia-spectrum disorders. By design, it pooled data only where possible, reporting long-term functional recovery at 35.4% (95% CI 22.6–49.4) and symptomatic remission at 39.6% (95% CI 29.3–50.5), and synthesizing the remaining questions narratively. The overarching message is cautiously hopeful: recovery is attainable for a substantial minority of individuals; structured psychosocial interventions are associated with meaningful functional gains; and the cognitive, social, personal, and ecological processes that shape recovery are increasingly, if still imperfectly, understood.

At the same time, the evidence tempers optimism with realism. Functional recovery and remission rates remain below 40% under the present pooling; both estimates carry very high heterogeneity and only low certainty, and the pooled association between psychological mediators and outcome was weak and imprecise (r = 0.23; 95% CI −0.09–0.51, very low certainty). Personal recovery, the dimension most valued by people with lived experience, follows a course only partly coupled to clinical status and is the least amenable to conventional, protocol-driven intervention. The Multi-Pathway Dynamic Recovery Model (MPDR) proposed here is therefore offered not as a validated structure but as an empirically informed, testable framework that organizes evidence from six domains into falsifiable propositions for clinical practice, service redesign, and research.

Three overarching messages emerge: First, time matters: the early illness phase is a window of maximum leverage, and services should provide comprehensive, multicomponent psychosocial intervention from first contact rather than after treatment failure. Second, targets matter: social cognition, motivation, internalized stigma, and positive psychological resources recur across the narrative evidence as plausibly higher-yield intervention targets than basic neurocognitive deficits or positive symptoms alone. Third, context matters: individual-level interventions realize their potential only when embedded within supportive social ecologies that provide stable housing, meaningful employment, financial security, and freedom from discrimination.

The central challenge for the next decade is to move from description to mechanism, from average effects to personalized prediction, and from controlled efficacy to real-world implementation; methodologically, it means shifting from heterogeneous abstract-level evidence toward standardized, individual-participant data that can support robust quantitative synthesis. By making explicit what the current evidence can and cannot support, and by translating that evidence into the testable propositions of the MPDR, this review aims to advance both the science and the practice of recovery-oriented care for schizophrenia-spectrum disorders.

## Figures and Tables

**Table 1:** Research questions, thematic clusters, synthesis mode, and headline findings.

RQ	Thematic Cluster	Synthesis Mode	k (Pooled)	Headline Finding [95% CI]
RQ1	Long-Term Course & Outcome	Meta-analysis (2 outcomes)	11/7	Recovery 35.4% [22.6, 49.4] (k = 11); Remission 39.6% [29.3, 50.5] (k = 7)
RQ2	Psychological & Combined Treatment	Narrative	—	Qualitative synthesis
RQ3	Cognitive Predictors	Narrative	—	Qualitative synthesis
RQ4	Rehabilitation & Community Integration	Narrative	—	Qualitative synthesis
RQ5	Personal Recovery & QoL	Narrative	—	Qualitative synthesis
RQ6	Mediators & Resilience	Meta-analysis	7	r = 0.23 [−0.09, 0.51]

Note. k = number of independent primary studies contributing to each pool after de-duplication and exclusion of secondary syntheses. Recovery and remission proportions were pooled using the Freeman–Tukey double-arcsine transformation; mediator associations were analyzed using Fisher’s z; all analyses were conducted under DerSimonian–Laird random-effects models. Both proportion estimates were robust to including reviews and meta-analyses in sensitivity analyses (recovery: 35.6%; remission: 42.6%). RQ2–RQ5 are synthesized narratively because fewer than five independent, comparably measured effect sizes were available for pooling.

**Table 2:** Research questions, thematic clusters, and synthesis-mode assignment.

RQ	Thematic Cluster	Synthesis Mode	Basis
RQ1	Long-Term Course & Outcome	Meta-analysis (2 outcomes)	≥5 independent primary studies; recovery (k = 11) and remission (k = 7) pooled separately
RQ2	Psychological & Combined Treatment	Narrative	<5 independent primary effect sizes
RQ3	Cognitive Predictors	Narrative	<5 independent primary effect sizes
RQ4	Rehabilitation & Community Integration	Narrative	<5 independent primary effect sizes
RQ5	Personal Recovery & QoL	Narrative	<5 independent primary effect sizes
RQ6	Mediators, Resilience & Health Promotion	Meta-analysis	≥5 independent primary studies; associations pooled (k = 7)

Note. Pooled k values are independent primary studies after de-duplication and exclusion of secondary syntheses (Section 2.8). RQ1 is reported as two outcomes that are not combined into a single estimate.

**Table 3:** Consolidated screening provenance and reference ranges.

Stage	*n*	Note
Combined records (87-field + 83-field databases)	550	Two largely distinct extraction passes
Unique consolidated corpus	467	83 shared records removed (DOI/title)
On-topic papers	418	49 off-topic removed
With extractable statistics	377	41 without statistics removed
Included for synthesis	368	5 off-topic removed on detailed screen; refs [171,172,173,174,175,176,177,178,179,180,181,182,183,184,185,186,187,188,189,190,191,192,193,194,195,196,197,198,199,200,201,202,203,204,205,206,207,208,209,210,211,212,213,214,215,216,217,218,219,220,221,222,223,224,225,226,227,228,229,230,231,232,233,234,235,236,237,238,239,240,241,242,243,244,245,246,247,248,249,250,251,252,253,254,255,256,257,258,259,260,261,262,263,264,265,266,267,268,269,270,271,272,273,274,275,276,277,278,279,280,281,282,283,284,285,286,287,288,289,290,291,292,293,294,295,296,297,298,299,300,301,302,303,304,305,306,307,308,309,310,311,312,313,314,315,316,317,318,319,320,321,322,323,324,325,326,327,328,329,330,331,332,333,334,335,336,337,338,339,340,341,342,343,344,345,346,347,348,349,350,351,352,353,354,355,356,357,358,359,360,361,362,363,364,365,366,367,368,369,370,371,372,373,374,375,376,377,378,379,380,381,382,383,384,385,386,387,388,389,390,391,392,393,394,395,396,397,398,399,400,401,402,403,404,405,406,407,408,409,410,411,412,413,414,415,416,417,418,419,420,421,422,423,424,425,426,427,428,429,430,431,432,433,434,435,436,437,438,439,440,441,442,443,444,445,446,447,448,449,450,451,452,453,454,455,456,457,458,459,460,461,462,463,464,465,466,467,468,469,470,471,472,473,474,475,476,477,478,479,480,481,482,483,484,485,486,487,488,489,490,491,492,493,494,495,496,497,498,499,500,501,502,503,504,505,506,507,508,509,510,511,512,513,514,515,516,517,518,519,520,521,522,523,524,525,526,527,528,529,530,531,532,533,534,535,536,537,538]
RQ1 Long-term course & outcome	113	[171,172,173,174,175,176,177,178,179,180,181,182,183,184,185,186,187,188,189,190,191,192,193,194,195,196,197,198,199,200,201,202,203,204,205,206,207,208,209,210,211,212,213,214,215,216,217,218,219,220,221,222,223,224,225,226,227,228,229,230,231,232,233,234,235,236,237,238,239,240,241,242,243,244,245,246,247,248,249,250,251,252,253,254,255,256,257,258,259,260,261,262,263,264,265,266,267,268,269,270,271,272,273,274,275,276,277,278,279,280,281,282,283]
RQ2 Psychological & combined treatments	31	[284,285,286,287,288,289,290,291,292,293,294,295,296,297,298,299,300,301,302,303,304,305,306,307,308,309,310,311,312,313,314]
RQ3 Cognitive predictors	48	[315,316,317,318,319,320,321,322,323,324,325,326,327,328,329,330,331,332,333,334,335,336,337,338,339,340,341,342,343,344,345,346,347,348,349,350,351,352,353,354,355,356,357,358,359,360,361,362]
RQ4 Rehabilitation & community integration	40	[363,364,365,366,367,368,369,370,371,372,373,374,375,376,377,378,379,380,381,382,383,384,385,386,387,388,389,390,391,392,393,394,395,396,397,398,399,400,401,402]
RQ5 Personal recovery & QoL	49	[403,404,405,406,407,408,409,410,411,412,413,414,415,416,417,418,419,420,421,422,423,424,425,426,427,428,429,430,431,432,433,434,435,436,437,438,439,440,441,442,443,444,445,446,447,448,449,450,451]
RQ6 Mediators, resilience & health promotion	87	[452,453,454,455,456,457,458,459,460,461,462,463,464,465,466,467,468,469,470,471,472,473,474,475,476,477,478,479,480,481,482,483,484,485,486,487,488,489,490,491,492,493,494,495,496,497,498,499,500,501,502,503,504,505,506,507,508,509,510,511,512,513,514,515,516,517,518,519,520,521,522,523,524,525,526,527,528,529,530,531,532,533,534,535,536,537,538]
Independent primary studies in quantitative pools	25	Recovery 11 + Remission 7 + RQ6 7

Note. Pool counts are not mutually exclusive: eight studies reporting both recovery and remission contribute to both proportion pools.

**Table 4:** Prognostic factors for long-term outcome (narrative synthesis).

Prognostic Factor	Direction	Consistency (Illustrative Refs)
Shorter duration of untreated psychosis	Favorable	Consistent
Better premorbid adjustment	Favorable	Consistent [191]
Female sex	Favorable	Moderately consistent [208]
Medication adherence	Favorable	Moderately consistent
Family support/involvement	Favorable	Moderately consistent
Early-intervention service enrolment	Favorable	Consistent [272]
Prominent baseline negative symptoms	Unfavorable	Consistent [191]
Substance-use comorbidity	Unfavorable	Consistent
Frequent insomnia/sleep disturbance	Unfavorable	Emerging [231]
Social isolation at baseline	Unfavorable	Moderately consistent
Poorer cognitive functioning	Unfavorable	Consistent (overlaps RQ3)

Note. Directions reflect qualitative synthesis; no pooled effect estimates are reported, as comparably measured independent effect sizes were unavailable.

**Table 5:** Mediators and moderators of long-term recovery (direction of association; narrative).

Construct	Direction	Domain
Positive mental health	Facilitator	Psychological
Social support	Facilitator	Social
Therapeutic alliance	Facilitator	Treatment
Self-efficacy	Facilitator	Psychological
Hope/optimism	Facilitator	Psychological
Internalized stigma	Barrier	Psychological
Substance-use comorbidity	Barrier	Clinical
Duration of untreated psychosis	Barrier	Clinical
Persistent negative symptoms	Barrier	Clinical

Note. Only the direction of association is reported; the pooled correlation (r = 0.23) aggregates these heterogeneous constructs, which is why between-study heterogeneity approaches 100%.

**Table 6:** Summary of pooled findings and GRADE certainty.

Outcome (RQ)	k	N	Pooled Estimate [95% CI]	Certainty
RQ1 Recovery	11	3531	35.4% [22.6, 49.4]	Low
RQ1 Remission	7	1153	39.6% [29.3, 50.5]	Low
RQ6 Mediators (r)	7	13,934	r = 0.23 [−0.09, 0.51]	Very low

Note. Random-effects (DerSimonian–Laird); proportions via Freeman–Tukey, association via Fisher’s z. Pools comprise only independent primary studies; reviews/meta-analyses were included solely in sensitivity analyses. RQ2–RQ5 synthesized narratively (k < 5).

**Table 7:** Mapping of the MPDR components to the evidence and its status.

MPDR Component	Proposed Mechanism	Supporting Studies (k; References)	Quantitative Anchor	Status
Recovery Tier Architecture	Ordered recovery criteria (remission → recovery → integration → personal recovery)	k = 139; RQ1 [171,172,173,174,175,176,177,178,179,180,181,182,183,184,185,186,187,188,189,190,191,192,193,194,195,196,197,198,199,200,201,202,203,204,205,206,207,208,209,210,211,212,213,214,215,216,217,218,219,220,221,222,223,224,225,226,227,228,229,230,231,232,233,234,235,236,237,238,239,240,241,242,243,244,245,246,247,248,249,250,251,252,253,254,255,256,257,258,259,260,261,262,263,264,265,266,267,268,269,270,271,272,273,274,275,276,277,278,279,280,281,282,283] & RQ5 [403,404,405,406,407,408,409,410,411,412,413,414,415,416,417,418,419,420,421,422,423,424,425,426,427,428,429,430,431,432,433,434,435,436,437,438,439,440,441,442,443,444,445,446,447,448,449,450,451], e.g., [171,184,197,208,219,230,243,255,266,281,412,422,432,442]	Recovery 35.4%, Remission 39.6% (pooled, k = 11/7)	Pooled—Low
Cognitive Bottleneck	Social-cognitive & motivational mediation of cognition → function	k = 45; RQ3 [315,316,317,318,319,320,321,322,323,324,325,326,327,328,329,330,331,332,333,334,335,336,337,338,339,340,341,342,343,344,345,346,347,348,349,350,351,352,353,354,355,356,357,358,359,360,361,362], e.g., [315,318,321,325,329,333,337,340,343,346,350,353,356,359]	Not pooled (narrative)	Narrative—consistent
Window of Opportunity	Disproportionate leverage of the early illness phase	k = 56; RQ1/RQ2/RQ4 clusters, e.g., [171,177,181,185,190,203,216,225,241,250,261,271,294,365]	Not pooled (narrative)	Narrative—consistent
Social-Ecological Embedding	Micro-, meso- & macrosystem influences on outcome	k = 54; RQ4 [363,364,365,366,367,368,369,370,371,372,373,374,375,376,377,378,379,380,381,382,383,384,385,386,387,388,389,390,391,392,393,394,395,396,397,398,399,400,401,402] & RQ6, e.g., [364,369,376,380,384,390,396,452,457,464,472,490,503,518]	Not pooled (narrative)	Narrative—qualitative
Feedback Loops (facilitators/barriers)	Self-reinforcing & impeding mediator pathways	k = 36; RQ6 [452,453,454,455,456,457,458,459,460,461,462,463,464,465,466,467,468,469,470,471,472,473,474,475,476,477,478,479,480,481,482,483,484,485,486,487,488,489,490,491,492,493,494,495,496,497,498,499,500,501,502,503,504,505,506,507,508,509,510,511,512,513,514,515,516,517,518,519,520,521,522,523,524,525,526,527,528,529,530,531,532,533,534,535,536,537,538], e.g., [454,457,464,466,475,480,486,494,497,503,515,523,529,533]	Mediator r = 0.23 (pooled, k = 7)	Pooled—Very low
Tipping-Point/Nonlinear Dynamics	Self-sustaining recovery threshold	k = 34; bidirectional/trajectory studies, e.g., [188,191,193,215,245,258,270,423,454,472,493,504,529,532]	Theoretical inference	Preliminary—untested

Note. k denotes the number of corpus studies whose findings support each component; only the recovery/remission tiers (k = 11/7) and the mediator pathway (k = 7) are quantitatively pooled, while the larger supporting sets reflect narrative evidence across the relevant clusters. The reference lists are representative subsets; the indicated RQ ranges provide the full sets. The framework is hypothesis-generating and requires prospective testing.

## Data Availability

No new data were created or analyzed in this study. Data sharing is not applicable to this article.

## Supplementary Materials

The following supporting information can be downloaded at https://www.mdpi.com/article/10.3390/brainsci16090993/s1, Table S1: Individual study characteristics of the 368 included studies; Table S2: Extended study-level data (objectives, main findings, intervention effects, outcomes measured, and extracted statistics) for the 368 included studies; Table S3: Moderator variable definitions, coding rules, and inter-rater agreement; Table S4: Dual-adjudicated study-level risk-of-bias matrix for the 24 studies contributing to the quantitative pools; Table S5: GRADE summary of findings by research question; Table S6: Sensitivity and influence diagnostics, comprising leave-one-out analyses (S6a), alternative model specifications and primary-versus-sensitivity comparisons (S6b), and influence diagnostics (S6c); Table S7: PRISMA 2020 checklist; and Table S8: Extraction-integrity audit verdicts for the 51 studies with an extractable effect size.

## Abbreviations

The following abbreviations are used in this manuscript:

ACT	Assertive community treatment
CBT	Cognitive-behavioral therapy
CBTp	Cognitive-behavioral therapy for psychosis
CHIME	Connectedness, Hope and optimism, Identity, Meaning, Empowerment (personal-recovery framework)
CHR	Clinical high risk
CI	Confidence interval
DSM	Diagnostic and Statistical Manual of Mental Disorders
EI	Early intervention
EIS	Early-intervention service(s)
FEP	First-episode psychosis
GRADE	Grading of Recommendations, Assessment, Development and Evaluations
HR	Hazard ratio
ICD	International Classification of Diseases
IPS	Individual Placement and Support
MOOSE	Meta-analysis Of Observational Studies in Epidemiology
MPDR	Multi-Pathway Dynamic Recovery (model)
NOS	Newcastle–Ottawa Scale
OR	Odds ratio
PRISMA	Preferred Reporting Items for Systematic Reviews and Meta-Analyses
PROSPERO	International Prospective Register of Systematic Reviews
QoL	Quality of life
RCT	Randomized controlled trial
RoB	Risk of bias
RQ	Research question
SMD	Standardized mean difference
SSD	Schizophrenia-spectrum disorder(s)
TSSEM	Two-stage structural equation modeling

## References

[1] Peritogiannis V., Ninou A., Samakouri M. (2022). Mortality in Schizophrenia-Spectrum Disorders: Recent Advances in Understanding and Management. Healthcare.

[2] Hegde P.R., Suhas S., Kumar C.N., Benegal V. (2023). Schizophrenia spectrum disorders in India: A population-based study. Indian J. Psychiatry.

[3] Zhang S., Chen Y., Zhang L., Yang X., Dong J., Lu M., Zhou Y. (2026). Global lifetime prevalence of schizophrenia: A systematic review and meta-analysis. Mol. Psychiatry.

[4] Ristic B., Zanini A., Schimmenti S., Maselli F.M.C., Vassos E. (2025). Environmental risk factors for schizophrenia spectrum disorders around the globe: A mapping review of the literature. Epidemiol. Psychiatr. Sci..

[5] Zhan Z., Wang J., Shen T. (2025). Results of the Global Burden of Disease study for schizophrenia: Trends from 1990 to 2021 and projections to 2050. Front. Psychiatry.

[6] Burns A.V., Marder S. (2023). Schizophrenia spectrum and other psychotic disorders. Atlas of Psychiatry.

[7] Khoso A.B., Noureen A., Nisa Z.U., Hodkinson A., Elahi A., Arshad U., Naz A., Bhatti M.M., Asif M., Husain M.O. (2023). Prevalence of Suicidal Ideation and Suicide Attempts in Individuals with Psychosis and Bipolar Disorder in South Asia: Systematic Review and Meta-Analysis. BJPsych Open.

[8] Winter L.D., Jelsma A., Vermeulen J.M., Vellinga A. (2025). Interrelationships of long-term changes in recovery domains among patients with schizophrenia spectrum disorders: A six-year follow-up study. BMC Psychiatry.

[9] Luo X., Wang Y., Wu Y., Huang Q., Wang Z., Wu Z., Guo H. (2025). Comparative analysis of the efficacy of UBE-PLIF versus conventional PLIF in the treatment of L4–5 degenerative spondylolisthesis. J. Orthop. Surg. Res..

[10] Hansen H.G., Speyer H., Albert N., Hjorthøj C., Eplov L.F., Nordentoft M. (2023). Clinical recovery among individuals with a first-episode schizophrenia: An updated systematic review and meta-analysis. Schizophr. Bull..

[11] Peralta V., Jalón E., Moreno-Izco L., Peralta D., Janda L., Sánchez-Torres A.M. (2022). Long-term outcomes of first-admission psychosis: A naturalistic 21-year follow-up study of symptomatic, functional and personal recovery and their baseline predictors. Schizophr. Bull..

[12] Li L., Rami F.Z., Lee B.M., Kim W.S., Kim S.W., Lee B.J., Chung Y.C. (2022). Three-year outcomes and predictors for full recovery in patients with early-stage psychosis. Schizophrenia.

[13] Yildirim M., Madera-McDonough J., Arango C., Correll C.U., Kane J.M. (2025). Assessment of functional recovery in patients with schizophrenia, with a focus on early-phase disease: Results from a Delphi consensus and narrative review. BMC Psychiatry.

[14] Griffiths S.L., Wood S.J., Upthegrove R. (2022). Heterogeneity in treatment outcomes and incomplete recovery in first episode psychosis: Does one size fit all?. Transl. Psychiatry.

[15] Béchard L., Desmeules C., Bachand L., Huot-Lavoie M., Corbeil O., Anderson E., Roy M. (2024). The effects of antipsychotic discontinuation or maintenance on the process of recovery in remitted first-episode psychosis patients: A systematic review and meta-analysis of randomized controlled trials. Eur. Psychiatry.

[16] Leamy M., Bird V., Le Boutillier C., Williams J., Slade M. (2011). Conceptual framework for personal recovery in mental health. Br. J. Psychiatry.

[17] Kreis I.V., Flaaten C.B., Widing L., Engen M.J., Melle I. (2024). Long-term clinical recovery and treatment resistance in first-episode psychosis: A 10-year follow-up study. Schizophrenia.

[18] Fässler L., Leucht S. (2024). Targeted psychological and psychosocial interventions for auditory hallucinations in persons with psychotic disorders: Protocol for a systematic review and meta-analysis. PLoS ONE.

[19] Lu E.Y., Cheng A.S.K., Tsang H.W.H., Chen J., Leung S., Yip A., Ma N. (2022). Psychoeducation, motivational interviewing, cognitive remediation training, and/or social skills training in combination for psychosocial functioning of patients with schizophrenia spectrum disorders: A systematic review and meta-analysis of randomized controlled trials. Front. Psychiatry.

[20] Niemeyer H., Opper F., Holmes E.A. (2026). Psychological interventions for persons with co-occurring psychotic and traumatic-stress symptoms: A systematic review and meta-analysis. Schizophr. Bull..

[21] Sighvatsson M.B., Sigurdsson J.F. (2023). How effective psychological treatments work: Mechanisms of change in cognitive behavioural therapy and beyond. Behav. Cogn. Psychother..

[22] Ong C.W., Ciarrochi J., Hofmann S.G., Karekla M., Hayes S.C. (2024). Through the extended evolutionary meta-model, and what ACT found there: ACT as a process-based therapy. J. Context. Behav. Sci..

[23] Theodoratou M., Farmakopoulou I., Gkintoni E., Sofologi M., Kougioumtzis G., Koundourou C. (2023). ADHD, Comorbidities, and Multimodal Treatment: Case Study Vignette. Perspectives of Cognitive, Psychosocial, and Learning Difficulties From Childhood to Adulthood: Practical Counseling Strategies.

[24] Lincoln T.M., Schulze L., Renneberg B. (2022). The role of emotion regulation in the characterization, development and treatment of psychopathology. Nat. Rev. Psychol..

[25] Ciarrochi J., Hernández C., Hill D., Ong C., Gloster A.T., Levin M.E., Hayes S.C. (2024). Process-based therapy: A common ground for understanding and utilizing therapeutic practices. J. Psychother. Integr..

[26] Firth J., López-Gil J.F., Milton A., Lambert J., Firth J.A. (2024). From “online brains” to “online lives”: Understanding the individualized impacts of Internet use across psychological, cognitive and social dimensions. World Psychiatry.

[27] Raugh I.M., Strauss G.P. (2024). Integrating mindfulness into the extended process model of emotion regulation: The dual-mode model of mindful emotion regulation. Emotion.

[28] Hall D.A., Hoare D.J., Fackrell K., Horobin A., Hogan N. (2022). The Core Rehabilitation Outcome Set for Single-Sided Deafness (CROSSSD) study: International consensus on outcome measures for trials of interventions for adults with single-sided deafness. Trials.

[29] Turner D.T., van der Gaag M., Karyotaki E., Cuijpers P. (2014). Psychological interventions for psychosis: A meta-analysis. Am. J. Psychiatry.

[30] Bighelli I., Rodolico A., García-Mieres H., Pitschel-Walz G., Hansen W.-P., Schneider-Thoma J., Siafis S., Wu H., Wang D., Salanti G. (2021). Psychosocial and psychological interventions for relapse prevention in schizophrenia: Network meta-analysis. Lancet Psychiatry.

[31] Xue W., Duan A.I., Zhao Y.H., Zuo H., Isleem H.F. (2025). AI-enabled multimodal monitoring for enhanced safety and functional recovery in elderly and post-stroke care: A mixed-methods study. Digit. Health.

[32] Roop S., Ahmed M., Dubas M., Gieselman M. (2026). Neurocognition, metacognition, and outcome in schizophrenia spectrum disorders: A scoping review. Int. J. Cogn. Sci..

[33] Theodoratou M., Puri B.K. (2025). An Integrated Treatment Approach for Bipolar II Disorder: A Clinical Case Study. J. Clin. Med..

[34] Aprile S.F., Rodolico A., Munafò A., Bighelli I., Caraci F., Leucht S. (2026). Dose reduction and discontinuation of antipsychotics in psychotic disorders: A systematic review of qualitative studies and meta-synthesis. Schizophrenia.

[35] Agarwal S.M., Dissanayake J., Agid O., Bowie C., Brierley N. (2023). Characterization and prediction of individual functional outcome trajectories in schizophrenia spectrum disorders (PREDICTS study): Study protocol. PLoS ONE.

[36] Berendsen S., Haan L., Bartels-Velthuis A.A., Simons C.J. (2022). Association of cognitive performance with clinical staging in schizophrenia spectrum disorders: A prospective 6-year follow-up study. Schizophr. Res. Cogn..

[37] Calzavara-Pinton I., Nibbio G., Barlati S., Bertoni L., Necchini N., Zardini D., Vita A. (2024). Treatment of cognitive impairment associated with schizophrenia spectrum disorders: New evidence, challenges, and future perspectives. Brain Sci..

[38] Gkintoni E., Vantarakis A., Gourzis P. (2026). P300 Event-Related Potentials as Cognitive Biomarkers in Neurological and Neuropsychiatric Disorders: A Systematic Review. Rev. Neurol..

[39] Hansen H.G., Hjorthøj C., Albert N., Lewandowski K.E., Glenthøj L.B., Nordentoft M. (2024). Twenty-year neurocognitive development following a schizophrenia spectrum disorder and associations with symptom severity and functional outcomes. Psychol. Med..

[40] He X.Y., Wei A.P., Huang Z.H., Wang F., Guo L.L., Hou C.L. (2025). Cognitive impairment across the schizophrenia spectrum: A comparative neuropsychological study. Front. Psychiatry.

[41] Gebreegziabhere Y., Habatmu K., Mihretu A., Cella M., Alem A. (2022). Cognitive impairment in people with schizophrenia: An umbrella review. Eur. Arch. Psychiatry Clin. Neurosci..

[42] Sağlam Y., Turan S., Karakuş O.B. (2023). Neurocognitive and social cognitive impairments in remission and symptomatic states of early-onset schizophrenia spectrum disorders. Eur. Child Adolesc. Psychiatry.

[43] Moritz S., Xie J., Penney D., Bihl L., Elmers J. (2023). The magnitude of neurocognitive impairment is overestimated in depression: The role of motivation, debilitating momentary influences, and the overreliance on mean differences. Psychol. Med..

[44] Romanowska S., Best M.W., Bowie C.R., Depp C.A., Patterson T.L., Penn D.L., Harvey P.D. (2022). Examining the association of life course neurocognitive ability with real-world functioning in schizophrenia-spectrum disorders. Schizophr. Res. Cogn..

[45] Giordano G.M., Sanmarchi F., Mucci A., Rucci P., Brando F., Caporusso E., Maj M. (2024). External validation of the five domains of negative symptoms: Focus on cognition, functional capacity, and real-world functioning. Eur. Psychiatry.

[46] Gkintoni E., Vantarakis A., Gourzis P. (2025). Neuroimaging Insights into the Public Health Burden of Neuropsychiatric Disorders: A Systematic Review of Electroencephalography-Based Cognitive Biomarkers. Medicina.

[47] Mahmood Z., Parrish E.M., Keller A.V., Lykins H.C., Pickell D., Granholm E., Twamley E.W. (2022). Modifiable predictors of self-reported and performance-based functioning in individuals with schizophrenia-spectrum disorders and high levels of negative symptoms. J. Psychiatr. Res..

[48] Giordano G.M., Pezzella P., Mucci A., Austin S.F., Erfurth A., Glenthøj B., Sachs G. (2024). Negative symptoms and social cognition as mediators of the relationship between neurocognition and functional outcome in schizophrenia. Front. Psychiatry.

[49] Lee K.H., Yu C.H. (2024). Relationships among neurocognition, self-defeatist beliefs, experiential negative symptoms, and social functioning in patients diagnosed with schizophrenia spectrum disorders. BMC Psychiatry.

[50] Arvaniti A. (2025). Driving performance in schizophrenia: The role of neurocognitive correlates—A systematic review. Brain Sci..

[51] Frawley E., Cowman M., Lepage M., Donohoe G. (2023). Social and occupational recovery in early psychosis: A systematic review and meta-analysis of psychosocial interventions. Psychol. Med..

[52] Harvey C., Brasier C., Brophy L., Ennals P., Fletcher J., Hamilton B. (2022). Community-based social interventions for people with severe mental illness: A systematic review and narrative synthesis of recent evidence. World Psychiatry.

[53] Rensburg A.J., Brooke-Sumner C. (2023). Intersectoral and multisectoral approaches to enable recovery for people with severe mental illness in low- and middle-income countries: A scoping review. Camb. Prism. Glob. Ment. Health.

[54] Genk C., Roeg D. (2023). Current insights of community mental healthcare for people with severe mental illness: A scoping review. Front. Psychiatry.

[55] Cheng W.L., Chang C.C., Griffiths M.D., Yen C.F., Liu J.H., Su J.A. (2022). Quality of life and care burden among family caregivers of people with severe mental illness: Mediating effects of self-esteem and psychological distress. BMC Psychiatry.

[56] Austin S.F., Mors O., Secher R.G., Hjorthøj C.R., Albert N., Bertelsen M., Jensen H., Jeppesen P., Petersen L., Randers L. (2013). Predictors of Recovery in First Episode Psychosis: The OPUS Cohort at 10-Year Follow-Up. Schizophr. Res..

[57] Huang Y., Orr M., Govindasamy S., Hielscher E., McLaren H. (2025). Interventions supporting meaningful connections for people with serious mental illness: A concept-framed systematic narrative review. Soc. Psychiatry Psychiatr. Epidemiol..

[58] Frank J., Mustard C., Smith P., Siddiqi A., Cheng Y., Burdorf A. (2023). Work as a social determinant of health in high-income countries: Past, present, and future. The Lancet.

[59] Petranovich C.L., Person-Jones K., Koerber S., Lantagne A., Graber S., Sarmiento C.A., Kirkwood M.W. (2025). Health care, educational, and vocational transitions in young adults with pediatric-onset disabilities: Associations with social determinants of health. Arch. Phys. Med. Rehabil..

[60] Suiter S.V., Meadows M.L. (2023). Educational attainment and educational contexts as social determinants of health. Prim. Care Clin. Off. Pract..

[61] Egede L.E., Walker R.J., Williams J.S. (2023). Addressing structural inequalities, structural racism, and social determinants of health: A vision for the future. J. General. Intern. Med..

[62] Han S., Zhang R.F., Shi L., Richie R., Liu H., Tseng A., Tsui F.R. (2022). Classifying social determinants of health from unstructured electronic health records using deep learning-based natural language processing. J. Biomed. Inform..

[63] Gkintoni E., Pallis E.G., Bitsios P., Giakoumaki S.G. (2017). Neurocognitive Performance, Psychopathology and Social Functioning in Individuals at High Risk for Schizophrenia or Psychotic Bipolar Disorder. J. Affect. Disord..

[64] Harvey C., Brasier C., Ennals P. (2023). Community-based models of care facilitating the recovery of people living with persistent and complex mental health needs: A systematic review and narrative synthesis. Front. Psychiatry.

[65] Bond G.R., Drake R.E., Becker D.R. (2020). An Update on Individual Placement and Support. World Psychiatry.

[66] Mueser K.T., Deavers F., Penn D.L., Cassisi J.E. (2013). Psychosocial treatments for schizophrenia. Annu. Rev. Clin. Psychol..

[67] Dixon L.B., Dickerson F., Bellack A.S., Bennett M., Dickinson D., Goldberg R.W., Lehman A., Tenhula W.N., Calmes C., Pasillas R.M. (2010). The 2009 schizophrenia PORT psychosocial treatment recommendations. Schizophr. Bull..

[68] Chien W.T., Leung S.F., Yeung F.K., Wong W.K. (2013). Current approaches to treatments for schizophrenia spectrum disorders. Neuropsychiatr. Dis. Treat..

[69] O’Sullivan D.J., Bearne L.M., Harrington J.M., Cardoso J.R., McVeigh J.G. (2024). The effectiveness of social prescribing in the management of long-term conditions in community-based adults: A systematic review and meta-analysis. Clin. Rehabil..

[70] Smith J.L., Deighton K., Innes A.Q., Holl M., Liao Z., Kelly B.M. (2023). Improved clinical outcomes in response to a 12-week blended digital and community-based long-COVID-19 rehabilitation programme. Front. Med..

[71] Fulford K.W.M. (2023). Recovery-oriented person-centered mental health care yesterday, today, and tomorrow: A proposed phenomenological response to the double challenges of contemporary recovery-oriented person-centered mental health care. Front. Psychol..

[72] Lorenz-Artz K., Bongers I. (2023). Unraveling complexity in changing mental health care towards person-centered care. Front. Psychiatry.

[73] Ragins M., Sunkel C. (2024). The recovery model and other rehabilitative approaches. Tasman’s Psychiatry.

[74] Lorenz-Artz K., Bongers I. (2026). Transforming specialized mental health practice: Insights from clients’ perspectives on how to support recovery. PLoS Ment. Health.

[75] Jørgensen K. (2025). Political dynamics and user engagement in mental health practice toward recovery. The Path to Mental Health Recovery.

[76] Tucker J.A. (2025). Whole person recovery from substance use disorder: A call for research examining a dynamic behavioral ecological model of contexts supportive of recovery. Addctn. Res. Theory.

[77] Pérez-Ramos A., Plasse J., Boudet I., Gouache B., Legros-Lafarge E. (2026). Mapping personal recovery in schizophrenia spectrum disorders: An exploratory machine learning study of self-reported stage classifications. Front. Public Health.

[78] van Eck R.M., Burger T.J., Vellinga A., Schirmbeck F., de Haan L. (2018). The relationship between clinical and personal recovery in schizophrenia spectrum disorders: Meta-analysis. Schizophr. Bull..

[79] Fazakas I., Fuchs T., Thoma S., Bois C., Gozé T. (2025). Cracks in the pattern: Gallagher’s theory of the self and the dynamics of schizophrenic selfhood. Front. Psychiatry.

[80] Marsicano G., Deodato M., Melcher D. (2026). Atypical integration of temporal evidence and priors in causality judgment along the autism-schizotypy continuum. iScience.

[81] Hao J., Hansen H.G., Hjorthøj C., Nordentoft M., Veling W., Alizadeh B.Z. (2026). Childhood Premorbid Adjustment as a Marker for 20-Year Impairments in Schizophrenia Spectrum Disorder: Results from the OPUS Study. Eur. Child Adolesc. Psychiatry.

[82] Kubzansky L.D., Kim E.S., Boehm J.K., Davidson R.J., Huffman J.C., Loucks E.B., Moskowitz J.T. (2023). Interventions to modify psychological well-being: Progress, promises, and an agenda for future research. Affect. Sci..

[83] Oikonomou V., Gkintoni E., Halkiopoulos C., Karademas E.C. (2024). Quality of Life and Incidence of Clinical Signs and Symptoms among Caregivers of Persons with Mental Disorders: A Cross-Sectional Study. Healthcare.

[84] Gkintoni E., Vassilopoulos S.P., Nikolaou G. (2025). Mindfulness-based cognitive therapy in clinical practice: A systematic review of neurocognitive outcomes and applications for mental health and well-being. J. Clin. Med..

[85] Andreu Y., Soto-Rubio A., Ramos-Campos M., Escriche-Saura A., Martínez M., Gavilá J. (2022). Impact of hormone therapy side effects on health-related quality of life, distress, and well-being of breast cancer survivors. Sci. Rep..

[86] Boucher E., Honomichl R., Ward H., Powell T., Stoeckl S.E., Parks A. (2022). The effects of a digital well-being intervention on older adults: Retrospective analysis of real-world user data. JMIR Aging.

[87] Li X., Ren Z., Ji T., Shi H., Zhao H., He M., Zhang X. (2022). Associations of sleep quality, anxiety symptoms and social support with subjective well-being among Chinese perimenopausal women. J. Affect. Disord..

[88] Theodoratou M., Katsarou D.V., Mantsos E., Denazi E., Megari K. (2025). Innovative Interventions for Child Mental Health. Groundbreaking Approaches to Early Childhood Development and Therapy.

[89] Headrick L., Newman D.A., Park Y.A., Liang Y. (2023). Recovery experiences for work and health outcomes: A meta-analysis and recovery-engagement-exhaustion model. J. Bus. Psychol..

[90] Deen M.L. (2024). Illness (self-)management, clinical and functional recovery as determinants of personal recovery in people with severe mental illnesses: A mediation analysis. PLoS ONE.

[91] Gkintoni E. (2023). Clinical Neuropsychological Characteristics of Bipolar Disorder, with a Focus on Cognitive and Linguistic Pattern: A Conceptual Analysis. F1000Research.

[92] Gkintoni E., Skokou M., Gourzis P. (2024). Integrating Clinical Neuropsychology and Psychotic Spectrum Disorders: A Systematic Analysis of Cognitive Dynamics, Interventions, and Underlying Mechanisms. Medicina.

[93] White R.L., Vella S., Biddle S.J.H., Sutcliffe J., Guagliano J.M., Uddin R. (2024). Physical activity and mental health: A systematic review and best-evidence synthesis of mediation and moderation studies. Int. J. Behav. Nutr. Phys. Act..

[94] Theodoratou M., Kalafatis D., Panitsa G. (2020). The Impact of Physical Activity on Mental Health and Psychological Well-Being, Perspectives on Improving the Educational Curriculum. J. Psychol. Neurosci..

[95] Gangwani R., Cain A., Collins A., Cassidy J.M. (2022). Leveraging factors of self-efficacy and motivation to optimize stroke recovery. Front. Neurol..

[96] McCollum R., Berrian H., Theobald S., Kollie K., Dean L. (2022). Barriers and enablers to health-seeking for people affected by severe stigmatising skin diseases (SSSDs): A scoping review. Soc. Sci..

[97] Pitanupong J., Aunjitsakul W. (2024). Personal and perceived stigma in relation to diverse domains of quality of life among patients with major depressive disorder having residual symptoms: A hospital-based study. Qual. Life Res..

[98] Laquidara J.R., Furgason K., Banks L.M., Saavedra S., Lincoln S.H. (2025). Perceived impacts of internalized stigma in individuals with schizophrenia and schizoaffective disorder. Psychiatr. Rehabil. J..

[99] Valery K.-M., Prouteau A. (2020). Schizophrenia Stigma in Mental Health Professionals and Stigma-Reduction Interven-tions: A Systematic Review. Psychiatry Res..

[100] Ghader N., Al-Bashaireh A.M., Al-Omari H. (2024). Exploring risk perception, mental health, mental fatigue, stigma, and quality of life among UAE healthcare workers during the COVID-19 pandemic: A national multicentric cross-sectional study. Int. J. Environ. Res. Public Health.

[101] Aremu T.O., Oluwole O.E., Adeyinka K.O., Schommer J.C. (2022). Medication adherence and compliance: Recipe for improving patient outcomes. Pharmacy.

[102] Maarouf O.H., Fülöp T. (2022). A critical review of medication adherence in hypertension: Barriers and facilitators clinicians should consider. Patient Prefer. Adherence.

[103] Stewart S.J.F., Moon Z., Horne R. (2023). Medication nonadherence: Health impact, prevalence, correlates and interventions. Psychol. Health.

[104] Religioni U., Barrios-Rodríguez R., Requena P., Borowska M., Ostrowski J. (2025). Enhancing therapy adherence: Impact on clinical outcomes, healthcare costs, and patient quality of life. Medicina.

[105] Deng M., Zhai S., Ouyang X., Liu Z., Ross B. (2022). Factors influencing medication adherence among patients with severe mental disorders from the perspective of mental health professionals. BMC Psychiatry.

[106] Boamah S., Miao Y., Guo X., Fen Y., Dong W. (2023). Comprehensive effects of lifestyle reform, adherence, and related factors on hypertension control: A review. J. Clin. Hypertens..

[107] Dilas D., Flores R., Morales-García W.C., Calizaya-Milla Y.E., Morales-García M., Sairitupa-Sanchez L., Saintila J. (2023). Social support, quality of care, and patient adherence to tuberculosis treatment in Peru: The mediating role of nurse health education. Patient Prefer. Adherence.

[108] Guo A., Jin H., Mao J., Zhu W., Zhou Y., Ge X., Yu D. (2023). Impact of health literacy and social support on medication adherence in patients with hypertension: A cross-sectional community-based study. BMC Cardiovasc. Disord..

[109] Blanke E.S., Schmiedek F., Siebert S., Richter D., Brose A. (2023). Perspectives on resilience: Trait resilience, correlates of resilience in daily life, and longer-term change in affective distress. Stress Health.

[110] Gong X., Chen C., Tong X. (2024). Does grit compensate for family background disadvantage in predicting mental health difficulties? A longitudinal study of Chinese migrant and urban children. J. Youth Adolesc..

[111] Berke D.S., Liautaud M.M., Xu Y. (2026). Psychosocial contributions of discrimination to traumatic stress adaptation among transgender and nonbinary adults: A longitudinal examination. J. Trauma. Stress.

[112] McDonald M.A., Marquardt C.A. (2026). Resilience as a dynamic process among military recruits exposed to basic combat training stressors. Dev. Psychopathol..

[113] Chen J.J., Liu L.F., Chang S.M., Lu C.P. (2023). Identifying the top determinants of psychological resilience among community older adults during COVID-19 in Taiwan: A random forest approach. Mach. Learn. Appl..

[114] Slade M., Rennick-Egglestone S., Blackie L., Llewellyn-Beardsley J., Franklin D., Hui A., Thornicroft G., McGranahan R., Pollock K., Priebe S. (2019). Post-Traumatic Growth in Mental Health Recovery: Systematic Review of Reviews. BMJ Open.

[115] Liang M.Z., Liu M.L., Tang Y., Chen P., Ye Z.J. (2023). Heterogeneity in resilience patterns and its prediction of 1-year quality of life outcomes among patients with newly diagnosed cancer: An exploratory piecewise growth mixture model analysis. Eur. J. Oncol. Nurs..

[116] Ramirez-Perez M., Bohle P., Martin A. (2025). A comprehensive meta-analysis of the impact of intervention programmes on psychological capital development: Post-intervention and longer-term effects. Pers. Rev..

[117] Lo C.K.M., Chan K.L., Yu L., Chui W.W.H., Ip P. (2023). Long-term effects of psychosocial interventions on internet-related disorders: A meta-analysis. Comput. Hum. Behav..

[118] Duagi D., Carter B., Farrelly M., Lisk S., Shearer J., Byford S., Brown J.S.L. (2024). Long-term effects of psychosocial interventions for adolescents on depression and anxiety: A systematic review and meta-analysis. EClinicalMedicine.

[119] Micklitz H.M., Glass C.M., Bengel J., Sander L.B. (2024). Efficacy of psychosocial interventions for survivors of intimate partner violence: A systematic review and meta-analysis. Trauma Violence Abus..

[120] Hohenschurz-Schmidt D., Chan J., Soliman N., Vollert J., Vase L. (2024). Placebo analgesia in physical and psychological interventions: Systematic review and meta-analysis of three-armed trials. Eur. J. Pain.

[121] Andresen R., Oades L., Caputi P. (2003). The Experience of Recovery from Schizophrenia: Towards an Empirically Validated Stage Model. Aust. N. Z. J. Psychiatry.

[122] Puri B.K., Potoglou A., Kalaitzaki A., Yotsidi V., Theodoratou M. (2025). Evaluating Post-Traumatic Growth Among Healthcare Workers. AIMS Public Health.

[123] Nagy Z., Takács S., Császár-Nagy N. (2023). The effectiveness of psychological interventions for rheumatoid arthritis (RA): A systematic review and meta-analysis. Life.

[124] Hedges E.P., See C., Si S., McGuire P., Dickson H., Kempton M.J. (2022). Meta-analysis of longitudinal neurocognitive performance in people at clinical high-risk for psychosis. Psychol. Med..

[125] Mesholam-Gately R.I., Giuliano A.J., Goff K.P., Faraone S.V., Seidman L.J. (2009). Neurocognition in First-Episode Schizophrenia: A Meta-Analytic Review. Neuropsychology.

[126] Lee S., Hill T.R., Johnson B., Testa R., Priya V., Spencer-Smith M., Coghill D. (2022). Can neurocognitive outcomes assist measurement-based care for children with attention-deficit/hyperactivity disorder? A systematic review and meta-analyses of the relationships among changes in neurocognitive functions and clinical outcomes of attention-deficit/hyperactivity disorder in pharmacological and cognitive training interventions. J. Child Adolesc. Psychopharmacol..

[127] Pedruzo B., Aymerich C., Pacho M., Herrero J., Laborda M., Bordenave M., Catalan A. (2024). Longitudinal change in neurocognitive functioning in children and adolescents at clinical high risk for psychosis: A systematic review. Eur. Child Adolesc. Psychiatry.

[128] Metcalfe T., Nielsen T.R., O’Donald F., Franzen S., Calia C. (2026). A systematic review of the measurement and influence of quality of education on neuropsychological test performance in later life. Neuropsychol. Rev..

[129] Verschueren J. (2026). Neurocognitive deficits related to ligamentous ankle injuries: A systematic review. Sports Med. Open.

[130] Ninsiima H.I., Ainamani H.E., Ayebazibwe G., Matovu D., Eze E.D. (2026). Essential micronutrients and biguanides (metformin) synergistic and antagonistic interactions on neurocognitive outcomes in type two diabetes mellitus: A systematic review of preclinical and clinical evidence. Front. Endocrinol..

[131] Feitosa Filho H.N., Furtado J.F.C.U., Eulálio E.C., Ribeiro P.V.C., Dias F.C., Paiva L.M.A., Neto E.B. (2026). Neurocognitive performance in myelin oligodendrocyte glycoprotein antibody-associated disease: A systematic review and meta-analysis. Mult. Scler. Relat. Disord..

[132] Goikoetxea-Sotelo G., Hedel H.J.A. (2023). Defining, quantifying, and reporting intensity, dose, and dosage of neurorehabilitative interventions focusing on motor outcomes. Front. Rehabil. Sci..

[133] Schaefer S.K., Thomas L.M., Lindner S., Lieb K. (2023). World Health Organization’s low-intensity psychosocial interventions: A systematic review and meta-analysis of the effects of Problem Management Plus and Step-by-Step. World Psychiatry.

[134] Xue S., Akhter-Khan S.C., Wray B., Husain M.I., Lawrance E.L. (2024). Mental health and psychosocial interventions in the context of climate change: A scoping review. npj Ment. Health Res..

[135] Bognár S.A., Teutsch B., Bunduc S., Veres D.S., Szabó B., Fogarasi B., Hegyi P. (2024). Psychological intervention improves quality of life in patients with early-stage cancer: A systematic review and meta-analysis of randomized clinical trials. Sci. Rep..

[136] Hansen K.E., Brandsborg B., Kesmodel U.S., Forman A., Kold M., Pristed R., Vase L. (2023). Psychological interventions improve quality of life despite persistent pain in endometriosis: Results of a 3-armed randomized controlled trial. Qual. Life Res..

[137] Deste G., Corbo D., Nibbio G., Italia M., Dell’Ovo D., Calzavara-Pinton I., Vita A. (2023). Impact of physical exercise alone or in combination with cognitive remediation on cognitive functions in people with schizophrenia: A qualitative critical review. Brain Sci..

[138] Mundey P., Dopp A.R., Beasley L.O., Silovsky J.F., Eisenberg D. (2023). Qualitative economic evaluation of psychosocial interventions: How looking beyond the numbers helps illuminate programmatic costs and benefits. Qual. Psychol..

[139] Stocker J.E., Koppe G., Reich H., Heshmati S., Kittel-Schneider S., Hofmann S.G. (2023). Formalizing psychological interventions through network control theory. Sci. Rep..

[140] Page M.J., McKenzie J.E., Bossuyt P.M., Boutron I., Hoffmann T.C., Mulrow C.D., Shamseer L., Tetzlaff J.M., Akl E.A., Brennan S.E. (2021). The PRISMA 2020 Statement: An Updated Guideline for Reporting Systematic Reviews. BMJ.

[141] Stroup D.F., Berlin J.A., Morton S.C., Olkin I., Williamson G.D., Rennie D., Moher D., Becker B.J., Sipe T.A., Thacker S.B. (2000). Meta-Analysis of Observational Studies in Epidemiology: A Proposal for Reporting (MOOSE). JAMA.

[142] Ouzzani M., Hammady H., Fedorowicz Z., Elmagarmid A. (2016). Rayyan—A Web and Mobile App for Systematic Reviews. Syst. Rev..

[143] Babineau J. (2014). Product Review: Covidence (Systematic Review Software). J. Can. Health Libr. Assoc..

[144] Cohen J. (1960). Coefficient of Agreement for Nominal Scales. Educ. Psychol. Meas..

[145] Landis J.R., Koch G.G. (1977). The Measurement of Observer Agreement for Categorical Data. Biometrics.

[146] Sterne J.A.C., Savović J., Page M.J., Elbers R.G., Blencowe N.S., Boutron I., Cates C.J., Cheng H.-Y., Corbett M.S., Eldridge S.M. (2019). RoB 2: A Revised Tool for Assessing Risk of Bias in Randomised Trials. BMJ.

[147] Stang A. (2010). Critical evaluation of the Newcastle-Ottawa scale for the assessment of the quality of nonrandomized studies in meta-analyses. Eur. J. Epidemiol..

[148] Shea B.J., Reeves B.C., Wells G., Thuku M., Hamel C., Moran J., Moher D., Tugwell P., Welch V., Kristjansson E. (2017). AMSTAR 2: A Critical Appraisal Tool for Systematic Reviews That Include Randomised or Non-Randomised Studies of Healthcare Interventions. BMJ.

[149] Peters J.L., Sutton A.J., Jones D.R., Abrams K.R., Rushton L. (2008). Contour-Enhanced Meta-Analysis Funnel Plots Help Distinguish Publication Bias from Other Causes of Asymmetry. J. Clin. Epidemiol..

[150] Egger M., Davey Smith G., Schneider M., Minder C. (1997). Bias in Meta-Analysis Detected by a Simple, Graphical Test. BMJ.

[151] Begg C.B., Mazumdar M. (1994). Operating Characteristics of a Rank Correlation Test for Publication Bias. Biometrics.

[152] Duval S., Tweedie R. (2000). Trim and Fill: A Simple Funnel-Plot-Based Method of Testing and Adjusting for Publication Bias in Meta-Analysis. Biometrics.

[153] Thomas H. (1990). L. V. Hedges and I. Olkin. Statistical Methods for Meta-Analysis. New York: Academic Press, 1985. xxii + 369 pp., $62.50. Psychometrika.

[154] Peterson R.A., Brown S.P. (2005). On the Use of Beta Coefficients in Meta-Analysis. J. Appl. Psychol..

[155] Freeman M.F., Tukey J.W. (1950). Transformations Related to the Angular and the Square Root. Ann. Math. Stat..

[156] DerSimonian R., Laird N. (1986). Meta-Analysis in Clinical Trials. Control. Clin. Trials.

[157] Veroniki A.A., Jackson D., Viechtbauer W., Bender R., Bowden J., Knapp G., Kuss O., Higgins J.P.T., Langan D., Salanti G. (2016). Methods to estimate the between-study variance and its uncertainty in meta-analysis. Res. Synth. Methods.

[158] Higgins J.P.T., Thompson S.G., Deeks J.J., Altman D.G. (2003). Measuring Inconsistency in Meta-Analyses. BMJ.

[159] Cochran W.G. (1954). The Combination of Estimates from Different Experiments. Biometrics.

[160] IntHout J., Ioannidis J.P.A., Borm G.F. (2014). The Hartung-Knapp-Sidik-Jonkman Method for Random Effects Meta-Analysis Is Straightforward and Considerably Outperforms the Standard DerSimonian-Laird Method. BMC Med. Res. Methodol..

[161] Cheung M.W.-L. (2015). metaSEM: An R package for meta-analysis using structural equation modeling. Front. Psychol..

[162] Guyatt G.H., Oxman A.D., Vist G.E., Kunz R., Falck- Ytter Y., Alonso-Coello P., Schünemann H.J. (2008). GRADE: An emerging consensus on rating quality of evidence and strength of recommendations. BMJ.

[163] Balduzzi S., Rücker G., Schwarzer G. (2019). How to Perform a Meta-Analysis with R: A Practical Tutorial. Évid. Based Ment. Health.

[164] Harrer M., Cuijpers P., Furukawa T.A., Ebert D.D. (2021). Doing Meta-Analysis with R: A Hands-On Guide.

[165] Hedges L.V., Tipton E., Johnson M.C. (2010). Robust Variance Estimation in Meta-Regression with Dependent Effect Size Estimates. Res. Synth. Methods.

[166] R Core Team (2023). R: A Language and Environment for Statistical Computing.

[167] Viechtbauer W. (2010). Conducting Meta-Analyses in R with the metafor Package. J. Stat. Softw..

[168] Fisher Z., Tipton E. (2015). Robumeta: An R Package for Robust Variance Estimation in Meta-Analysis. arXiv.

[169] Wickham H. (2016). ggplot2: Elegant Graphics for Data Analysis.

[170] Higgins J.P.T., Thomas J., Chandler J., Cumpston M., Li T., Page M.J., Welch V.A. (2023). Cochrane Handbook for Systematic Reviews of Interventions, Version 6.4.

[171] Ajnakina O., Stubbs B., Francis E., Gaughran F., David A., Murray R., Lally J. (2021). Employment and relationship outcomes in first-episode psychosis: A systematic review and meta-analysis of longitudinal studies. Schizophr. Res..

[172] Albert U., Tomassi S., Maina G., Tosato S. (2018). Prevalence of non-psychotic disorders in ultra-high risk individuals and transition to psychosis: A systematic review. Psychiatry Res..

[173] Alvarez-Jimenez M., Gleeson J., Henry L., Harrigan S., Harris M., Killackey E., Bendall S., Amminger G., Yung A., Herrman H. (2011). Road to full recovery: Longitudinal relationship between symptomatic remission and psychosocial recovery in first-episode psychosis over 7.5 years. Psychol. Med..

[174] Andrews J. (2025). Do No Harm: Potential Iatrogenic Harm of School Mental Health Interventions. Eur. Psychiatry.

[175] Bachmann S., Degen C., Geider F., Schröder J. (2014). Neurological Soft Signs in the Clinical Course of Schizophrenia: Results of a Meta-Analysis. Front. Psychiatry.

[176] Baloush-Kleinman V., Levine S., Roe D., Shnitt D., Weizman A., Poyurovsky M. (2011). Adherence to antipsychotic drug treatment in early-episode schizophrenia: A six-month naturalistic follow-up study. Schizophr. Res..

[177] Baltazar L., Benedictis L.D., Abdel-Baki A., Lalonde P., Lesage A. (2021). Long term course and outcome of first episode schizophrenia: A 27-to-31-year follow-up. Soc. Psychiatry Psychiatr. Epidemiol..

[178] Beck K., Studerus E., Andreou C., Egloff L., Leanza L., Simon A., Borgwardt S., Riecher-Rössler A. (2019). Clinical and functional ultra-long-term outcome of patients with a clinical high risk (CHR) for psychosis. Eur. Psychiatry.

[179] Berthet P., Haatveit B., Kjelkenes R., Worker A., Kia S.M., Wolfers T., Rutherford S., Alnaes D., Dinga R., Pedersen M.L. (2025). A 10-Year Longitudinal Study of Brain Cortical Thickness in People with First-Episode Psychosis Using Normative Models. Schizophr. Bull..

[180] Bora E., Murray R. (2014). Meta-analysis of cognitive deficits in ultra-high risk to psychosis and first-episode psychosis: Do the cognitive deficits progress over, or after, the onset of psychosis?. Schizophr. Bull..

[181] Butler R., Berry K., Varese F., Bucci S. (2018). Are family warmth and positive remarks related to outcomes in psychosis? A systematic review. Psychol. Med..

[182] Cannon T.D. (2008). Neurodevelopment and the Transition from Schizophrenia Prodrome to Schizophrenia: Research Imperatives. Biol. Psychiatry.

[183] Carrión R., McLaughlin D., Goldberg T., Auther A., Olsen R.H., Olvet D., Correll C., Cornblatt B. (2013). Prediction of functional outcome in individuals at clinical high risk for psychosis. JAMA Psychiatry.

[184] Carrión R., Correll C., Auther A., Cornblatt B. (2017). A Severity-Based Clinical Staging Model for the Psychosis Prodrome: Longitudinal Findings From the New York Recognition and Prevention Program. Schizophr. Bull..

[185] Catalán A., McCutcheon R., Aymerich C., Pedruzo B., Radua J., Rodríguez V., Pablo G.S., Pacho M., Pérez J.L., Solmi M. (2024). The magnitude and variability of neurocognitive performance in first-episode psychosis: A systematic review and meta-analysis of longitudinal studies. Transl. Psychiatry.

[186] Chan S.K., So H., Hui C., Chang W., Lee E., Chung D., Tso S., Hung S., Yip K., Dunn E. (2014). 10-year outcome study of an early intervention program for psychosis compared with standard care service. Psychol. Med..

[187] Chan S., Chan H., Devlin J., Bastiampillai T., Mohan T., Hui C., Chang W., Lee E., Chen E. (2019). A systematic review of long-term outcomes of patients with psychosis who received early intervention services. Int. Rev. Psychiatry.

[188] Chan S., Chan H., Pang H., Hui C., Suen Y., Chang W., Lee E., Chen E. (2020). Ten-year trajectory and outcomes of negative symptoms of patients with first-episode schizophrenia spectrum disorders. Schizophr. Res..

[189] Chan S.Y., Brady R., Hwang M., Higgins A., Nielsen K., Öngür D., Hall M. (2020). Heterogeneity of Outcomes and Network Connectivity in Early-Stage Psychosis: A Longitudinal Study. Schizophr. Bull..

[190] Chan S., Chan H., Liao Y.J., Suen Y., Hui C., Chang W., Lee E., Chen E. (2022). Longitudinal relapse pattern of patients with first-episode schizophrenia-spectrum disorders and its predictors and outcomes: A 10-year follow-up study. Asian J. Psychiatry.

[191] Chang W., Ho R.W.H., Tang J., Wong C., Hui C., Chan S., Lee E.H.M., Suen Y., Chen E. (2018). Early-Stage Negative Symptom Trajectories and Relationships with 13-Year Outcomes in First-Episode Nonaffective Psychosis. Schizophr. Bull..

[192] Chen P., Harris K. (2019). Association of Positive Family Relationships with Mental Health Trajectories From Adolescence To Midlife. JAMA Pediatr..

[193] Chopra S., Fornito A., Francey S., O’Donoghue B., Cropley V., Nelson B., Graham J., Baldwin L., Tahtalian S., Yuen H. (2021). Differentiating the effect of antipsychotic medication and illness on brain volume reductions in first-episode psychosis: A Longitudinal, Randomised, Triple-blind, Placebo-controlled MRI Study. Neuropsychopharmacology.

[194] Cohen A., Patel V., Thara R., Gureje O. (2007). Questioning an Axiom: Better Prognosis for Schizophrenia in the Developing World?. Schizophr. Bull..

[195] Collip D., Wigman J., Lin A., Nelson B., Oorschot M., Vollebergh W., Ryan J., Baksheev G., Wichers M., Os J. (2013). Dynamic association between interpersonal functioning and positive symptom dimensions of psychosis over time: A longitudinal study of healthy adolescents. Schizophr. Bull..

[196] Crumlish N., Whitty P., Clarke M., Browne S., Kamali M., Gervin M., McTigue O., Kinsella A., Waddington J., Larkin C. (2009). Beyond the critical period: Longitudinal study of 8-year outcome in first-episode non-affective psychosis. Br. J. Psychiatry.

[197] Cumming C., Kinner S., Mcketin R., Li I., Preen D. (2020). Methamphetamine use, health and criminal justice system outcomes: A systematic review. Drug Alcohol Rev..

[198] Debbané M., Eliez S., Badoud D., Conus P., Flückiger R., Schultze-Lutter F. (2015). Developing psychosis and its risk states through the lens of schizotypy. Schizophr. Bull..

[199] Drake R., Mercer-McFadden C., Mueser K., McHugo G., Bond G. (1998). Review of integrated mental health and substance abuse treatment for patients with dual disorders. Schizophr. Bull..

[200] Dwyer D., Kalman J., Budde M., Kambeitz J., Ruef A., Antonucci L., Kambeitz-Ilankovic L., Hasan A., Kondofersky I., Anderson-Schmidt H. (2020). An Investigation of Psychosis Subgroups with Prognostic Validation and Exploration of Genetic Underpinnings: The PsyCourse Study. JAMA Psychiatry.

[201] Fisher H., Caspi A., Poulton R., Meier M., Houts R., Harrington H., Arseneault L., Moffitt T. (2013). Specificity of childhood psychotic symptoms for predicting schizophrenia by 38 years of age: A birth cohort study. Psychol. Med..

[202] Foussias G., Foussias G., Mann S., Mann S., Zakzanis K., Reekum R., Agid O., Remington G., Remington G. (2011). Prediction of longitudinal functional outcomes in schizophrenia: The impact of baseline motivational deficits. Schizophr. Res..

[203] Fusar-Poli P., Bechdolf A., Taylor M., Bonoldi I., Carpenter W., Yung A., McGuire P. (2013). At risk for schizophrenic or affective psychoses? A meta-analysis of DSM/ICD diagnostic outcomes in individuals at high clinical risk. Schizophr. Bull..

[204] Gil-Berrozpe G., Peralta V., Sánchez-Torres A., Moreno-Izco L., Jalón E.G., Peralta D., Janda L., Cuesta M. (2023). Psychopathological networks in psychosis: Changes over time and clinical relevance. A long-term cohort study of first-episode psychosis. Schizophr. Res..

[205] Goghari V., Harrow M., Grossman L., Rosen C. (2012). A 20-year multi-follow-up of hallucinations in schizophrenia, other psychotic, and mood disorders. Psychol. Med..

[206] Gouse B., Brown H.E. (2022). Improving Outcomes in Schizophrenia-A Case for Initiation of Long-Acting Antipsychotics in Early-Phase Illness. JAMA Netw. Open.

[207] Griffiths S., Leighton S., Mallikarjun P., Blake G., Everard L., Jones P.B., Fowler D., Hodgekins J., Amos T., Freemantle N. (2021). Structure and stability of symptoms in first episode psychosis: A longitudinal network approach. Transl. Psychiatry.

[208] Grossman L., Harrow M., Rosen C., Faull R., Strauss G. (2008). Sex differences in schizophrenia and other psychotic disorders: A 20-year longitudinal study of psychosis and recovery. Compr. Psychiatry.

[209] Hall W., Degenhardt L. (2008). Cannabis use and the risk of developing a psychotic disorder. World Psychiatry.

[210] Hall M., Holton K.M., Öngu□r D., Montrose D., Keshavan M. (2019). Longitudinal Trajectory of Early Functional Recovery in Patients with First Episode Psychosis. Schizophr. Res..

[211] Harding C., McCormick R.V., Strauss J., Ashikaga T., Brooks G.W. (1989). Computerised Life Chart Methods to Map Domains of Function and Illustrate Patterns of Interactions in the Long-Term Course Trajectories of Patients Who Once Met the Criteria for DSM-III Schizophrenia. Br. J. Psychiatry.

[212] Haro J., Altamura C., Corral R., Elkis H., Evans J., Krebs M., Zink M., Malla A., Méndez J.I., Bernasconi C. (2018). Understanding the course of persistent symptoms in schizophrenia: Longitudinal findings from the pattern study. Psychiatry Res..

[213] Harrow M., Sands J., Silverstein M., Goldberg J. (1997). Course and outcome for schizophrenia versus other psychotic patients: A longitudinal study. Schizophr. Bull..

[214] Harrow M., Grossman L., Herbener E., Davies E.W. (2000). Ten-year outcome: Patients with schizoaffective disorders, schizophrenia, affective disorders and mood-incongruent psychotic symptoms. Br. J. Psychiatry.

[215] Harrow M., Jobe T., Faull R. (2012). Do all schizophrenia patients need antipsychotic treatment continuously throughout their lifetime? A 20-year longitudinal study. Psychol. Med..

[216] Heurich M., Föcking M., Mongan D., Cagney G., Cotter D. (2021). Dysregulation of complement and coagulation pathways: Emerging mechanisms in the development of psychosis. Mol. Psychiatry.

[217] Huber G., Gross G., Schüttler R., Linz M. (1980). Longitudinal studies of schizophrenic patients. Schizophr. Bull..

[218] Iorfino F., Scott E., Carpenter J., Cross S., Hermens D., Killedar M., Nichles A., Zmicerevska N., White D., Guastella A. (2019). Clinical Stage Transitions in Persons Aged 12 to 25 Years Presenting to Early Intervention Mental Health Services with Anxiety, Mood, and Psychotic Disorders. JAMA Psychiatry.

[219] Iorfino F., Carpenter J., Cross S., Crouse J.J., Davenport T., Hermens D., Yee H., Nichles A., Zmicerevska N., Guastella A. (2021). Social and occupational outcomes for young people who attend early intervention mental health services: A longitudinal study. Med. J. Aust..

[220] Isohanni M., Miettunen J., Jääskeläinen E., Moilanen J., Hulkko A., Huhtaniska S. (2018). S230. Long-term antipsychotic medication in schizophrenia: Benefits, risks and follow-up: Data from finnish cohort studies and systematic review. Schizophr. Bull..

[221] James W., Preston N., Koh G., Spencer C., Kisely S., Castle D. (2004). A group intervention which assists patients with dual diagnosis reduce their drug use: A randomized controlled trial. Psychol. Med..

[222] Jaracz K., Górna K., Kiejda J., Grabowska-Fudala B., Jaracz J., Suwalska A., Rybakowski J.K. (2015). Psychosocial functioning in relation to symptomatic remission: A longitudinal study of first episode schizophrenia. Eur. Psychiatry.

[223] Jobe T., Harrow M. (2010). Schizophrenia Course, Long-Term Outcome, Recovery, and Prognosis. Curr. Dir. Psychol. Sci..

[224] Johnson S., O’Reilly H., Ni Y., Wolke D., Marlow N. (2019). Psychiatric Symptoms and Disorders in Extremely Preterm Young Adults at 19 Years of Age and Longitudinal Findings From Middle Childhood. J. Am. Acad. Child Adolesc. Psychiatry.

[225] Jones P.B., Tarrant C. (2000). Developmental precursors and biological markers for schizophrenia and affective disorders: Specificity and public health implications. Eur. Arch. Psychiatry Clin. Neurosci..

[226] Kelebie M., Fentahun S., Tadesse G., Nakie G., Medfu G., Fasil B., Rtbey G., Muche M., Gobezie M., Alazar A. (2025). Predictors of long-term outcome of patients with schizophrenia in Africa: Systematic review and meta-analysis. BMC Public Health.

[227] Kendrick D., O’Brien C., Christie N., Coupland C., Quinn C., Avis M., Barker M., Barnes J., Coffey F., Joseph S. (2011). The impact of injuries study. multicentre study assessing physical, psychological, social and occupational functioning post injury—A protocol. BMC Public Health.

[228] Krinzinger H., Hall C.L., Groom M., Ansari M.T., Banaschewski T., Buitelaar J., Carucci S., Coghill D., Danckaerts M., Dittmann R. (2019). Neurological and psychiatric adverse effects of long-term methylphenidate treatment in ADHD: A map of the current evidence. Neurosci. Biobehav. Rev..

[229] Kwapil T., Gross G.M., Silvia P., Barrantes-Vidal N. (2013). Prediction of psychopathology and functional impairment by positive and negative schizotypy in the Chapmans’ ten-year longitudinal study. J. Abnorm. Psychol..

[230] Lally J., Ajnakina O., Stubbs B., Cullinane M., Murphy K., Gaughran F., Murray R. (2017). Remission and recovery from first-episode psychosis in adults: Systematic review and meta-analysis of long-term outcome studies. Br. J. Psychiatry.

[231] Li S.X., Lam S., Zhang J., Yu M., Chan J., Chan C., Espie C., Freeman D., Mason O., Wing Y. (2016). Sleep Disturbances and Suicide Risk in an 8-Year Longitudinal Study of Schizophrenia-Spectrum Disorders. Sleep.

[232] Lin K.M., Kleinman A. (1988). Psychopathology and clinical course of schizophrenia: A cross-cultural perspective. Schizophr. Bull..

[233] Lobban F., Barrowclough C., Jones S. (2004). The impact of beliefs about mental health problems and coping on outcome in schizophrenia. Psychol. Med..

[234] Marengo J., Harrow M. (1997). Longitudinal courses of thought disorder in schizophrenia and schizoaffective disorder. Schizophr. Bull..

[235] McGorry P. (1994). The Influence of Illness Duration on Syndrome Clarity and Stability in Functional Psychosis: Does the Diagnosis Emerge and Stabilise with Time?. Aust. N. Z. J. Psychiatry (Print).

[236] McPherson S., Hengartner M. (2019). Long-term outcomes of trials in the National Institute for Health and Care Excellence depression guideline. BJPsych Open.

[237] Menezes N., Arenovich T., Zipursky R. (2006). A systematic review of longitudinal outcome studies of first-episode psychosis. Psychol. Med..

[238] Nickl-Jockschat T., Ho B., Andreasen N. (2020). Clinical and Neurobiological Predictors of Long-Term Outcome in Schizophrenia. Biol. Psychiatry.

[239] Nuechterlein K., Dawson M., Gitlin M., Ventura J., Goldstein M., Snyder K., Yee C.M., Mintz J. (1992). Developmental Processes in Schizophrenic Disorders: Longitudinal studies of vulnerability and stress. Schizophr. Bull..

[240] Pablo G.S., Besana F., Arienti V., Catalan A., Vaquerizo-Serrano J., Cabras A., Pereira J., Soardo L., Coronelli F., Kaur S. (2021). Longitudinal outcome of attenuated positive symptoms, negative symptoms, functioning and remission in people at clinical high risk for psychosis: A meta-analysis. eClinicalMedicine.

[241] Pablo G.S., Soardo L., Cabras A., Pereira J., Kaur S., Besana F., Arienti V., Coronelli F., Shin J., Solmi M. (2022). Clinical outcomes in individuals at clinical high risk of psychosis who do not transition to psychosis: A meta-analysis. Epidemiol. Psychiatr. Sci..

[242] Pearce E., Birken M., Pais S., Tamworth M., Ng Y., Wang J., Chipp B., Crane E., Schlief M., Yang J. (2023). Associations between constructs related to social relationships and mental health conditions and symptoms: An umbrella review. BMC Psychiatry.

[243] Peralta V., Moreno-Izco L., Jalón E.G., Sánchez-Torres A., Janda L., Peralta D., Fañanás L., Cuesta M. (2021). Prospective Long-Term Cohort Study of Subjects with First-Episode Psychosis Examining Eight Major Outcome Domains and Their Predictors: Study Protocol. Front. Psychiatry.

[244] Pieters L., Nadesalingam N., Walther S., Harten P.V. (2021). A systematic review of the prognostic value of motor abnormalities on clinical outcome in psychosis. Neurosci. Biobehav. Rev..

[245] Polari A., Lavoie S., Yuen H., Amminger P., Berger G., Chen E., DeHaan L., Hartmann J., Markulev C., Melville F. (2018). Clinical trajectories in the ultra-high risk for psychosis population. Schizophr. Res..

[246] Roche E., Creed L., MacMahon D., Brennan D., Clarke M. (2015). The Epidemiology and Associated Phenomenology of Formal Thought Disorder: A Systematic Review. Schizophr. Bull..

[247] Rogeau A., Boer A.J., Guedj E., Sala A., Sommer I.E., Veronese M., Weijden-Germann M., Van Weehaeghe D., Cecchin D., EANM Neuroimaging Committee (2024). EANM perspective on clinical PET and SPECT imaging in schizophrenia-spectrum disorders: A systematic review of longitudinal studies. Eur. J. Nucl. Med. Mol. Imaging.

[248] Rosen C., Grossman L., Harrow M., Bonner-Jackson A., Faull R. (2011). Diagnostic and prognostic significance of Schneiderian first-rank symptoms: A 20-year longitudinal study of schizophrenia and bipolar disorder. Compr. Psychiatry.

[249] Rosengard R., Malla A., Mustafa S.S., Iyer S., Joober R., Bodnar M., Lepage M., Shah J. (2019). Association of Pre-onset Subthreshold Psychotic Symptoms with Longitudinal Outcomes During Treatment of a First Episode of Psychosis. JAMA Psychiatry.

[250] Russo M., Levine S., Demjaha A., Forti M., Bonaccorso S., Fearon P., Dazzan P., Pariante C., David A., Morgan C. (2014). Association between symptom dimensions and categorical diagnoses of psychosis: A cross-sectional and longitudinal investigation. Schizophr. Bull..

[251] Rutigliano G., Valmaggia L., Landi P., Frascarelli M., Cappucciati M., Sear V., Rocchetti M., Micheli A.D., Jones C.J., Palombini E. (2016). Persistence or recurrence of non-psychotic comorbid mental disorders associated with 6-year poor functional outcomes in patients at ultra high risk for psychosis. J. Affect. Disord..

[252] Samamé C., Cattaneo B., Richaud M.C., Strejilevich S., Aprahamian I. (2021). The long-term course of cognition in bipolar disorder: A systematic review and meta-analysis of patient-control differences in test-score changes. Psychol. Med..

[253] Sands J., Harrow M. (1999). Depression during the longitudinal course of schizophrenia. Schizophr. Bull..

[254] Santesteban-Echarri O., Paino M., Rice S., González-Blanch C., McGorry P., Gleeson J., Alvarez-Jimenez M. (2017). Predictors of functional recovery in first-episode psychosis: A systematic review and meta-analysis of longitudinal studies. Clin. Psychol. Rev..

[255] Sarpal D., Robinson D., Lencz T., Argyelan M., Ikuta T., Karlsgodt K.H., Gallego J., Kane J., Szeszko P., Malhotra A. (2015). Antipsychotic Treatment and Functional Connectivity of the Striatum: A Prospective Controlled Study in First-Episode Schizophrenia. JAMA Psychiatry.

[256] Schlosser D.A., Zinberg J.L., Loewy R., Casey-Cannon S., Cannon T.D. (2010). Predicting the Longitudinal Effects of the Family Environment on Prodromal Symptoms and Functioning in Patients At-Risk for Psychosis. Schizophr. Res..

[257] Schlosser D.A., Jacobson S., Chen Q., Sugar C.A., Niendam T., Li G., Bearden C., Cannon T.D. (2012). Recovery from an at-risk state: Clinical and functional outcomes of putatively prodromal youth who do not develop psychosis. Schizophr. Bull..

[258] Shaw P., Gogtay N., Rapoport J. (2010). Childhood psychiatric disorders as anomalies in neurodevelopmental trajectories. Hum. Brain Mapp..

[259] Shehab A.A.A., Baltă A., Ciubară A., Burlea S., Carina V.D., Grigoraș M., Ciubară A. (2025). Early Warning Signs of Schizophrenia: Effective Strategies and the Benefits of Early Treatment. Eur. Psychiatry.

[260] Sigfrid L., Cevik M., Jesudason E., Lim W., Rello J., Amuasi J., Bozza F., Palmieri C., Munblit D., Holter J. (2021). What is the recovery rate and risk of long-term consequences following a diagnosis of COVID-19? A harmonised, global longitudinal observational study protocol. BMJ Open.

[261] Skalli L., Nicole L. (2011). Specialised first-episode psychosis services: A systematic review of the literature. L’Encephale.

[262] Skärsäter I., Langius A., Ågren H., Häggström L., Dencker K. (2005). Sense of coherence and social support in relation to recovery in first-episode patients with major depression: A one-year prospective study. Int. J. Ment. Health Nurs..

[263] Solmi F., Colman I., Weeks M., Lewis G., Kirkbride J. (2017). Trajectories of Neighborhood Cohesion in Childhood, and Psychotic and Depressive Symptoms at Age 13 and 18 Years. J. Am. Acad. Child Adolesc. Psychiatry.

[264] Stefaniak I., Sorokosz K., Janicki A., Wciórka J. (2019). Therapy based on avatar-therapist synergy for patients with chronic auditory hallucinations: A pilot study. Schizophr. Res..

[265] Strauss G., Harrow M., Grossman L., Rosen C. (2010). Periods of recovery in deficit syndrome schizophrenia: A 20-year multi-follow-up longitudinal study. Schizophr. Bull..

[266] Sullivan S., Hollén L., Wren Y., Thompson A., Lewis G., Zammit S. (2016). A longitudinal investigation of childhood communication ability and adolescent psychotic experiences in a community sample. Schizophr. Res..

[267] Svendsen I., Øie M., Møller P., Nelson B., Haug E., Melle I. (2019). Basic self-disturbances independently predict recovery in psychotic disorders: A seven year follow-up study. Schizophr. Res..

[268] Tay J.L., Ang Y., Tam W., Sim K. (2025). Accuracy of machine learning methods in predicting prognosis of patients with psychotic spectrum disorders: A systematic review. BMJ Open.

[269] Thai H., Audet É.C., Koestner R., Lepage M., O’Driscoll G.A. (2024). The role of motivation in clinical presentation, treatment engagement and response in schizophrenia-spectrum disorders: A systematic review. Clin. Psychol. Rev..

[270] Torgalsbøen A., Mohn C., Larøi F., Czajkowski N. (2025). Longitudinal recovery and self-efficacy in first-episode schizophrenia: Insights from a 10-year follow-up study. Front. Psychiatry.

[271] Trampush J., Lencz T., DeRosse P., John M., Gallego J., Petrides G., Hassoun Y., Zhang J., Addington J., Kellner C. (2015). Relationship of Cognition to Clinical Response in First-Episode Schizophrenia Spectrum Disorders. Schizophr. Bull..

[272] Tsiachristas A., Thomas T., Leal J., Lennox B. (2016). Economic impact of early intervention in psychosis services: Results from a longitudinal retrospective controlled study in England. BMJ Open.

[273] Voineskos A., Jacobs G.R., Ameis S. (2019). Neuroimaging Heterogeneity in Psychosis: Neurobiological Underpinnings and Opportunities for Prognostic and Therapeutic Innovation. Biol. Psychiatry.

[274] Vázquez-Bourgon J., Gómez-Revuelta M., Son J.M., Labad J., Foz V.O.G., Setién-Suero E., Ayesa-Arriola R., Tordesillas-Gutiérrez D., Juncal-Ruiz M., Crespo-Facorro B. (2022). Pattern of long-term weight and metabolic changes after a first episode of psychosis: Results from a 10-year prospective follow-up of the PAFIP program for early intervention in psychosis cohort. Eur. Psychiatry.

[275] Walker E., Cornblatt B., Addington J., Cadenhead K., Cannon T.D., McGlashan T., Perkins D., Seidman L., Tsuang M., Woods S. (2009). The relation of antipsychotic and antidepressant medication with baseline symptoms and symptom progression: A naturalistic study of the North American Prodrome Longitudinal Sample. Schizophr. Res..

[276] Weibell M., Hegelstad W., Auestad B., Bramness J., Evensen J., Haahr U., Joa I., Johannessen J.O., Larsen T., Melle I. (2017). The Effect of Substance Use on 10-Year Outcome in First-Episode Psychosis. Schizophr. Bull..

[277] Wigman J., Winkel R., Raaijmakers Q., Ormel J., Verhulst F., Reijneveld S., Os J.V., Vollebergh W. (2011). Evidence for a persistent, environment-dependent and deteriorating subtype of subclinical psychotic experiences: A 6-year longitudinal general population study. Psychol. Med..

[278] Winter L., Vermeulen J., Couwenbergh C., Weeghel J., Hasson-Ohayon I., Mulder C., Boonstra N., Veling W., Haan L. (2023). Short- and long-term changes in symptom dimensions among patients with schizophrenia spectrum disorders and different durations of illness: A meta-analysis. J. Psychiatr. Res..

[279] Woods S., Addington J., Cadenhead K., Cannon T.D., Cornblatt B., Heinssen R., Perkins D., Seidman L., Tsuang M., Walker E. (2009). Validity of the prodromal risk syndrome for first psychosis: Findings from the North American Prodrome Longitudinal Study. Schizophr. Bull..

[280] Yang Y., Clouston S.A.P., Reichenberg A., Callahan J.L., Ruggero C., Carlson G.A., Bromet E., Kotov R., Jonas K.G. (2025). Predictors and Outcomes Associated with 25-Year Cognitive Decline in Psychotic Disorders. Schizophr. Bull..

[281] Zammit S., Moore T., Lingford-Hughes A., Barnes T., Jones P.B., Burke M., Lewis G. (2008). Effects of cannabis use on outcomes of psychotic disorders: Systematic review. Br. J. Psychiatry.

[282] Zarski A., Berking M., Reis D., Lehr D., Buntrock C., Schwarzer R., Ebert D. (2018). Turning Good Intentions Into Actions by Using the Health Action Process Approach to Predict Adherence to Internet-Based Depression Prevention: Secondary Analysis of a Randomized Controlled Trial. J. Med. Internet Res..

[283] Zipursky R., Reilly T., Murray R. (2012). The Myth of Schizophrenia as a Progressive Brain Disease. Schizophr. Bull..

[284] Almeida O., Hankey G., Yeap B., Golledge J., Norman P., Flicker L. (2014). Mortality among People with Severe Mental Disorders Who Reach Old Age: A Longitudinal Study of a Community-Representative Sample of 37892 Men. PLoS ONE.

[285] Bauer S., Moessner M. (2012). Technology-enhanced monitoring in psychotherapy and e-mental health. J. Ment. Health.

[286] Bauer I., Gálvez J., Hamilton J.E., Balanzá-Martínez V., Zunta-Soares G., Soares J., Meyer T. (2016). Lifestyle interventions targeting dietary habits and exercise in bipolar disorder: A systematic review. J. Psychiatr. Res..

[287] Browne J., Harvey P.D., Buchanan R., Kelly D., Strauss G., Gold J., Holden J., Granholm E. (2022). A Longitudinal Examination of Real-World Sedentary Behavior in Adults with Schizophrenia-Spectrum Disorders in a Clinical Trial of Combined Oxytocin and Cognitive Behavioral Social Skills Training. Behav. Sci..

[288] Böge K., Hahne I., Bergmann N., Zierhut M., Ta T., Wingenfeld K., Bajbouj M., Hahn E. (2021). Mindfulness-based group therapy for inpatients with schizophrenia spectrum disorders—Feasibility, acceptability, and preliminary outcomes of a rater-blinded randomized controlled trial. Eur. Psychiatry.

[289] Dorsett P., Geraghty T. (2008). Health-related outcomes of people with spinal cord injury—A 10 year longitudinal study. Spinal Cord..

[290] Fusar-Poli P., Frascarelli M., Valmaggia L., Byrne M., Ståhl D., Rocchetti M., Codjoe L., Weinberg L., Tognin S., Xenaki L. (2014). Antidepressant, antipsychotic and psychological interventions in subjects at high clinical risk for psychosis: OASIS 6-year naturalistic study. Psychol. Med..

[291] Gassmann W., Christ O., Lampert J., Berger H. (2013). The influence of Antonovsky’s sense of coherence (SOC) and psychoeducational family intervention (PEFI) on schizophrenic outpatients’ perceived quality of life: A longitudinal field study. BMC Psychiatry.

[292] Hassan S., Ross J., Marston L., Osborn D., Walters K. (2019). Factors prospectively associated with physical activity and dietary related outcomes in people with severe mental illness: A systematic review of longitudinal studies. Psychiatry Res..

[293] Hastings P., Serbin L., Bukowski W., Helm J., Stack D., Dickson D., Ledingham J., Schwartzman A. (2019). Predicting psychosis-spectrum diagnoses in adulthood from social behaviors and neighborhood contexts in childhood. Dev. Psychopathol..

[294] Holt R., Hind D., Gossage-Worrall R., Bradburn M., Saxon D., McCrone P., Morris T.A., Etherington A., Shiers D., Barnard K. (2018). Structured lifestyle education to support weight loss for people with schizophrenia, schizoaffective disorder and first episode psychosis: The STEPWISE RCT. Health Technol. Assess..

[295] Huang S.L., Li R., Huang F.Y., Tang F.C. (2015). The Potential for Mindfulness-Based Intervention in Workplace Mental Health Promotion: Results of a Randomized Controlled Trial. PLoS ONE.

[296] Kani A.S., Shinn A.K., Lewandowski K., Öngür D. (2016). Converging Effects of Diverse Treatment Modalities on Frontal Cortex in Schizophrenia: A Review of Longitudinal Functional Magnetic Resonance Imaging Studies. J. Psychiatr. Res..

[297] Keuroghlian A., Frankenburg F., Zanarini M. (2013). The Relationship of Chronic Medical Illnesses, Poor Health-Related Lifestyle Choices, and Health Care Utilization to Recovery Status in Borderline Patients over a Decade of Prospective Follow-up. J. Psychiatr. Res..

[298] Kristén L., Ivarsson A., Parker J., Ziegert K. (2015). Future challenges for intervention research in health and lifestyle research—A systematic meta-literature review. Int. J. Qual. Stud. Health Well-Being.

[299] Kumari V., Fannon D., Peters E., Ffytche D., Sumich A., Premkumar P., Anilkumar A.P., Andrew C., Phillips M., Williams S.C.R. (2011). Neural changes following cognitive behaviour therapy for psychosis: A longitudinal study. Brain J. Neurol..

[300] Laurens K., Cullen A.E. (2015). Toward earlier identification and preventative intervention in schizophrenia: Evidence from the London Child Health and Development Study. Soc. Psychiatry Psychiatr. Epidemiol..

[301] Loizou S., Fowler D., Hayward M. (2022). Measuring the longitudinal course of voice hearing under psychological interventions: A systematic review. Clin. Psychol. Rev..

[302] Lunsford-Avery J.R., LeBourgeois M., Gupta T., Mittal V. (2015). Actigraphic-Measured Sleep Disturbance Predicts Increased Positive Symptoms in Adolescents at Ultra High-Risk for Psychosis: A Longitudinal Study. Schizophr. Res..

[303] Ma Y., Mumtaz S. (2025). The long-term mental health benefits of exercise training for physical education students: A comprehensive review of neurobiological, psychological, and social effects. Front. Psychiatry.

[304] McGorry P., Hartmann J., Spooner R., Nelson B. (2018). Beyond the “at risk mental state” concept: Transitioning to transdiagnostic psychiatry. World Psychiatry.

[305] Miniawi S.E., Orgeta V., Stafford J. (2022). Non-affective psychotic disorders and risk of dementia: A systematic review and meta-analysis. Psychol. Med..

[306] Mojtabai R., Fochtmann L., Chang S.W., Kotov R., Craig T., Bromet E. (2009). Unmet need for mental health care in schizophrenia: An overview of literature and new data from a first-admission study. Schizophr. Bull..

[307] Motl R., Ehde D., Shinto L., Fernhall B., LaRocca N., Zackowski K. (2020). Health Behaviors, Wellness, and Multiple Sclerosis Amid COVID-19. Arch. Phys. Med. Rehabil..

[308] Nagata J.M., Hur J.O., Talebloo J., Lee S., Choi W.W., Kim S.J., Lavender J.M., Moreno M.A. (2025). Problematic Social Media Use Interventions for Mental Health Outcomes in Adolescents. Curr. Psychiatry Rep..

[309] Paquin V., Lapierre M., Veru F., King S. (2021). Early Environmental Upheaval and the Risk for Schizophrenia. Annu. Rev. Clin. Psychol..

[310] Rief W., Glombiewski J.A. (2017). The role of expectations in mental disorders and their treatment. World Psychiatry.

[311] Rosenbaum B., Valbak K., Harder S., Knudsen P., Køster A., Lajer M., Lindhardt A., Winther G., Petersen L., Jørgensen P. (2005). The Danish National Schizophrenia Project: Prospective, comparative longitudinal treatment study of first-episode psychosis. Br. J. Psychiatry.

[312] Schweitzer R., Greben M., Bargenquast R. (2017). Long-term outcomes of Metacognitive Narrative Psychotherapy for people diagnosed with schizophrenia. Psychol. Psychother..

[313] Sousa A.E.F., Lepage M., Brodeur M. (2018). Longitudinal feasibility and acceptability of the express smartphone app: Recruitment, retention and preliminary findings. Schizophr. Bull..

[314] Yeager C., Shoji K., Luszczynska A., Benight C. (2018). Engagement with a Trauma Recovery Internet Intervention Explained with the Health Action Process Approach (HAPA): Longitudinal Study. JMIR Ment. Health.

[315] Antshel K., Fremont W., Ramanathan S., Kates W. (2016). Predicting Cognition and Psychosis in Young Adults with 22q11.2 Deletion Syndrome. Schizophr. Bull..

[316] Balanzá-Martínez V., Cuesta M., Arango C., Crespo-Facorro B., Tabarés-Seisdedos R. (2009). Longitudinal course of cognition in schizophrenia. Br. J. Psychiatry.

[317] Bellani M., Ricciardi C., Rossetti M.G., Zovetti N., Perlini C., Brambilla P. (2019). Cognitive remediation in schizophrenia: The earlier the better?. Epidemiol. Psychiatr. Sci..

[318] Bergh S., Hjorthøj C., Sørensen H., Fagerlund B., Austin S., Secher R.G., Jepsen J., Nordentoft M. (2016). Predictors and longitudinal course of cognitive functioning in schizophrenia spectrum disorders, 10years after baseline: The OPUS study. Schizophr. Res..

[319] Berry K., Barrowclough C., Wearden A. (2007). A review of the role of adult attachment style in psychosis: Unexplored issues and questions for further research. Clin. Psychol. Rev..

[320] Bonner-Jackson A., Grossman L., Harrow M., Rosen C. (2010). Neurocognition in schizophrenia: A 20-year multi-follow-up of the course of processing speed and stored knowledge. Compr. Psychiatry.

[321] Burdick K., Millett C.E. (2021). Cognitive heterogeneity is a key predictor of differential functional outcome in patients with bipolar disorder. Eur. Neuropsychopharmacol..

[322] Calvo D.T., Giménez-Donoso S., Setién-Suero E., Privat A.T., Crespo-Facorro B., Arriola R.A. (2017). Targeting recovery in first episode psychosis: The importance of neurocognition and premorbid adjustment in a 3-year longitudinal study. Schizophr. Res..

[323] Carey E., Gillan D., Healy C., Dooley N., Campbell D., McGrane J., O’Neill A., Coughlan H., Clarke M., Kelleher I. (2020). Early adult mental health, functional and neuropsychological outcomes of young people who have reported psychotic experiences: A 10-year longitudinal study. Psychol. Med..

[324] Censits D.M., Ragland J., Gur R., Gur R.E. (1997). Neuropsychological evidence supporting a neurodevelopmental model of schizophrenia: A longitudinal study. Schizophr. Res..

[325] Cowman M., Holleran L., Lonergan E., O’Connor K., Birchwood M., Donohoe G. (2021). Cognitive Predictors of Social and Occupational Functioning in Early Psychosis: A Systematic Review and Meta-analysis of Cross-Sectional and Longitudinal Data. Schizophr. Bull..

[326] Crutzen S., Gangadin S., Hua K.H., Veling W., Castelein S. (2025). The association of antipsychotic treatment and side effects with societal recovery and happiness: A naturalistic cohort study of people in long term care for a psychotic disorder. Eur. Psychiatry.

[327] Galderisi S., Mucci A., Gibertoni D., Rossi A., Rocca P., Bertolino A., Maj M. (2021). Predictors of real-life functioning in subjects with schizophrenia: A 4-year follow-up study. Eur. Psychiatry.

[328] Garety P. (1993). Psychological aspects of psychosis: Subjective meaning and longitudinal course. Curr. Opin. Psychiatry.

[329] Gitlin M., Miklowitz D. (2016). The difficult lives of individuals with bipolar disorder: A review of functional outcomes and their implications for treatment. J. Affect. Disord..

[330] Giuliani L., Giordano G., Bucci P., Pezzella P., Brando F., Galderisi S. (2021). Improving Knowledge on Pathways to Functional Outcome in Schizophrenia: Main Results From the Italian Network for Research on Psychoses. Front. Psychiatry.

[331] Gur R.E., McDonald-McGinn D., Moore T., Gallagher R., McClellan E.J., White L., Ruparel K., Hillman N., Crowley T., McGinn D. (2023). Psychosis spectrum features, neurocognition and functioning in a longitudinal study of youth with 22q11.2 deletion syndrome. Psychol. Med..

[332] Halverson T., Orleans-Pobee M., Merritt C.C., Sheeran P., Fett A.K., Penn D. (2019). Pathways to Functional Outcomes in Schizophrenia Spectrum Disorders: Meta-Analysis of Social Cognitive and Neurocognitive Predictors. Neurosci. Biobehav. Rev..

[333] Heine J., Kopp U., Klag J., Ploner C., Prüss H., Finke C. (2021). Long-Term Cognitive Outcome in Anti–N-Methyl-D-Aspartate Receptor Encephalitis. Ann. Neurol..

[334] Henderson A. (2013). The impact of social cognition training on recovery from psychosis. Curr. Opin. Psychiatry.

[335] Herbener E., Harrow M. (2004). Are negative symptoms associated with functioning deficits in both schizophrenia and nonschizophrenia patients? A 10-year longitudinal analysis. Schizophr. Bull..

[336] Horan W., Green M.F., DeGroot M., Fiske A., Hellemann G., Kee K., Kern R., Lee J., Sergi M., Subotnik K. (2012). Social cognition in schizophrenia, Part 2: 12-month stability and prediction of functional outcome in first-episode patients. Schizophr. Bull..

[337] Irani F., Kalkstein S., Moberg E., Moberg P. (2011). Neuropsychological performance in older patients with schizophrenia: A meta-analysis of cross-sectional and longitudinal studies. Schizophr. Bull..

[338] Jiménez-López E., Sánchez-Morla E., López-Villarreal A., Aparicio A., Martínez-Vizcaíno V., Vieta E., Rodríguez-Jiménez R., Santos J. (2018). Neurocognition and functional outcome in patients with psychotic, non-psychotic bipolar I disorder, and schizophrenia. A five-year follow-up. Eur. Psychiatry.

[339] Khandaker G., Barnett J., White I., Jones P.B. (2011). A quantitative meta-analysis of population-based studies of premorbid intelligence and schizophrenia. Schizophr. Res..

[340] Kucharska-Pietura K., Mortimer A. (2013). Can Antipsychotics Improve Social Cognition in Patients with Schizophrenia?. CNS Drugs.

[341] Lam M., Lee J., Rapisarda A., See Y., Yang Z., Lee S.A., Abdul-Rashid N.A., Kraus M., Subramaniam M., Chong S. (2018). Longitudinal Cognitive Changes in Young Individuals at Ultrahigh Risk for Psychosis. JAMA Psychiatry.

[342] Lee R., Lee R., Hermens D., Redoblado-Hodge M., Naismith S., Porter M., Kaur M., White D., Scott E., Hickie I. (2013). Neuropsychological and Socio-Occupational Functioning in Young Psychiatric Outpatients: A Longitudinal Investigation. PLoS ONE.

[343] Lee R., Hermens D., Naismith S., Lagopoulos J., Jones A.M., Scott J., Chitty K., White D., Robillard R., Scott E. (2015). Neuropsychological and functional outcomes in recent-onset major depression, bipolar disorder and schizophrenia-spectrum disorders: A longitudinal cohort study. Transl. Psychiatry.

[344] Leeson V., Sharma P., Harrison M., Ron M., Barnes T., Joyce E. (2009). IQ Trajectory, Cognitive Reserve, and Clinical Outcome Following a First Episode of Psychosis: A 3-Year Longitudinal Study. Schizophr. Bull..

[345] Levy B., Medina A.M., Weiss R. (2013). Cognitive and psychosocial functioning in bipolar disorder with and without psychosis during early remission from an acute mood episode: A comparative longitudinal study. Compr. Psychiatry.

[346] Lin A., Lin A., Wood S., Wood S., Nelson B., Brewer W., Spiliotacopoulos D., Bruxner A., Broussard C., Pantelis C. (2011). Neurocognitive predictors of functional outcome two to 13years after identification as ultra-high risk for psychosis. Schizophr. Res..

[347] McCleery A., Nuechterlein K. (2019). Cognitive impairment in psychotic illness: Prevalence, profile of impairment, developmental course, and treatment considerations. Dialogues Clin. Neurosci..

[348] Milic B., Feller C., Schneider M., Debbané M., Loeffler-Stastka H. (2021). Social cognition in individuals with 22q11.2 deletion syndrome and its link with psychopathology and social outcomes: A review. BMC Psychiatry.

[349] Mollon J., David A., Zammit S., Lewis G., Reichenberg A. (2018). Course of Cognitive Development From Infancy to Early Adulthood in the Psychosis Spectrum. JAMA Psychiatry.

[350] Mueser K., Salyers M.P., Mueser P. (2001). A prospective analysis of work in schizophrenia. Schizophr. Bull..

[351] Méndez R., Balanzá-Martínez V., Luperdi S.C., Estrada I., Latorre A., González-Jiménez P., Bouzas L., Yépez K., Ferrando A., Reyes S. (2021). Long-term neuropsychiatric outcomes in COVID-19 survivors: A 1-year longitudinal study. J. Intern. Med..

[352] Nuechterlein K., Subotnik K., Green M.F., Ventura J., Asarnow R., Gitlin M., Yee C.M., Gretchen-Doorly D., Mintz J. (2011). Neurocognitive predictors of work outcome in recent-onset schizophrenia. Schizophr. Bull..

[353] Perry B., Salimkumar D., Green D., Meakin A.M.B., Gibson A., Mahajan D., Tahir T.A., Singh S. (2017). Associated illness severity in schizophrenia and diabetes mellitus: A systematic review. Psychiatry Res..

[354] Punsoda-Puche P., Barajas A., Mamano-Grande M., Jiménez-Lafuente A., Ochoa S. (2024). Relationship between social cognition and premorbid adjustment in psychosis: A systematic review. Schizophrenia.

[355] Roell L., Lindner C., Maurus I., Keeser D., Malchow B., Schmitt A., Falkai P. (2024). Neural and somatic mechanisms driving clinical improvements in post-acute schizophrenia spectrum disorders. Eur. Psychiatry.

[356] Seidman L., Shapiro D., Stone W., Woodberry K., Ronzio A., Cornblatt B., Addington J., Bearden C., Cadenhead K., Cannon T.D. (2016). Neurocognition and Transition to Psychosis: Baseline Functioning in the Second Phase of the North American Prodrome Longitudinal Study. JAMA Psychiatry.

[357] Shmukler A., Gurovich I., Agius M., Agius M., Agius M., Zaytseva Y. (2015). Long-term trajectories of cognitive deficits in schizophrenia: A critical overview. Eur. Psychiatry.

[358] Sui J., Pearlson G., Du Y., Yu Q., Jones T., Chen J., Jiang T., Bustillo J., Calhoun V. (2015). In Search of Multimodal Neuroimaging Biomarkers of Cognitive Deficits in Schizophrenia. Biol. Psychiatry.

[359] Tabarés-Seisdedos R., Balanzá-Martínez V., Sánchez-Moreno J., Martínez-Àran A., Salazar-Fraile J., Selva-Vera G., Rubio C., Mata I., Gómez-Beneyto M., Vieta E. (2008). Neurocognitive and clinical predictors of functional outcome in patients with schizophrenia and bipolar I disorder at one-year follow-up. J. Affect. Disord..

[360] Taylor J.H., Calkins M., Gur R. (2020). Markers of Psychosis Risk in the General Population. Biol. Psychiatry.

[361] Theodoridou A., Heekeren K., Dvorsky D., Metzler S., Franscini M., Haker H., Kawohl W., Rüsch N., Walitza S., Rössler W. (2014). Early Recognition of High Risk of Bipolar Disorder and Psychosis: An Overview of the ZInEP “Early Recognition” Study. Front. Public Health.

[362] Torgalsbøen A., Mohn C., Larøi F., Fu S., Czajkowski N. (2023). A ten-year longitudinal repeated assessment study of cognitive improvement in patients with first-episode schizophrenia and healthy controls: The Oslo Schizophrenia Recovery (OSR) study. Schizophr. Res..

[363] Bakken T.L., Hellerud J.M.A., Kildahl A., Solheim-Inderberg A.M., Hove O., Helverschou S.B. (2024). Schizophrenia in Autistic People with Intellectual Disabilities. Treatment and Interventions. J. Autism Dev. Disord..

[364] Brekke J., Ansel M., Long J., Slade E., Weinstein M. (1999). Intensity and continuity of services and functional outcomes in the rehabilitation of persons with schizophrenia. Psychiatr. Serv..

[365] Burton C., Tso I., Carrión R., Niendam T., Adelsheim S., Auther A., Cornblatt B., Carter C., Melton R., Sale T.G. (2019). Baseline Psychopathology and Relationship to Longitudinal Functional Outcome in Attenuated and Early First Episode Psychosis. Schizophr. Res..

[366] Chan C.T., Abdin E., Subramaniam M., Tay S., Lim L.K., Verma S. (2019). Two-Year Clinical and Functional Outcomes of an Asian Cohort at Ultra-High Risk of Psychosis. Front. Psychiatry.

[367] Drake R., Luciano A., Mueser K., Covell N.H., Essock S., Xie H., McHugo G. (2015). Longitudinal Course of Clients with Co-occurring Schizophrenia-Spectrum and Substance Use Disorders in Urban Mental Health Centers: A 7-Year Prospective Study. Schizophr. Bull..

[368] Geng F., Xu T., Wang Y., Shi H., Yan C., Neumann D., Shum D., Lui S., Cheung E., Chan R. (2013). Developmental trajectories of schizotypal personality disorder-like behavioural manifestations: A two-year longitudinal prospective study of college students. BMC Psychiatry.

[369] Green M.F., Kern R., Heaton R. (2004). Longitudinal studies of cognition and functional outcome in schizophrenia: Implications for MATRICS. Schizophr. Res..

[370] Harding C. (1988). Course types in schizophrenia: An analysis of European and American studies. Schizophr. Bull..

[371] Harrow M., Hansford B., Astrachan-Fletcher E.B. (2009). Locus of control: Relation to schizophrenia, to recovery, and to depression and psychosis—A 15-year longitudinal study. Psychiatry Res..

[372] Harrow M., Jobe T., Faull R., Yang J. (2017). A 20-Year Multi-Followup Longitudinal Study Assessing Whether Antipsychotic Medications Contribute to Work Functioning in Schizophrenia. Psychiatry Res..

[373] Herrman H. (1998). Long-term outcome and rehabilitation. Curr. Opin. Psychiatry.

[374] Hijdra R., Robroek S., Sadigh Y., Burdorf A., Schuring M. (2024). The effects of an interdisciplinary employment program on paid employment and mental health among persons with severe mental disorders. Int. Arch. Occup. Environ. Health.

[375] Hunter R., Cameron R., Norrie J. (2009). Using patient-reported outcomes in schizophrenia: The Scottish Schizophrenia Outcomes Study. Psychiatr. Serv..

[376] Jackson F., Foti D., Kotov R., Perlman G., Mathalon D., Proudfit G.H. (2014). An incongruent reality: The N400 in relation to psychosis and recovery. Schizophr. Res..

[377] Jester D.J., Thomas M.L., Sturm E.T., Harvey P.D., Keshavan M., Davis B.J., Saxena S., Tampi R., Leutwyler H., Compton M.T. (2023). Review of Major Social Determinants of Health in Schizophrenia-Spectrum Psychotic Disorders: I. Clinical Outcomes. Schizophr. Bull..

[378] Jones N., Tong L., Pagdon S., Ebuenyi I., Harrow M., Sharma R.P., Rosen C. (2024). Using latent class analysis to investigate enduring effects of intersectional social disadvantage on long-term vocational and financial outcomes in the 20-year prospective Chicago Longitudinal Study. Psychol. Med..

[379] Kee K., Green M.F., Mintz J., Brekke J. (2003). Is emotion processing a predictor of functional outcome in schizophrenia?. Schizophr. Bull..

[380] Killaspy H., Marston L., Green N., Harrison I., Lean M., Holloway F., Craig T., Leavey G., Arbuthnott M., Koeser L. (2016). Clinical outcomes and costs for people with complex psychosis; a naturalistic prospective cohort study of mental health rehabilitation service users in England. BMC Psychiatry.

[381] Korman N., Shah S., Suetani S., Kendall K., Rosenbaum S., Dark F., Nadareishvili K., Siskind D. (2018). Evaluating the Feasibility of a Pilot Exercise Intervention Implemented Within a Residential Rehabilitation Unit for People with Severe Mental Illness: GO HEART: (Group Occupational Health Exercise and Rehabilitation Treatment). Front. Psychiatry.

[382] Lachman M., Agrigoroaei S. (2010). Promoting Functional Health in Midlife and Old Age: Long-Term Protective Effects of Control Beliefs, Social Support, and Physical Exercise. PLoS ONE.

[383] Liberman R. (2002). Future directions for research studies and clinical work on recovery from schizophrenia: Questions with some answers. Int. Rev. Psychiatry.

[384] Liljeholm U., Argentzell E., Bejerholm U. (2020). An integrated mental health and vocational intervention: A longitudinal study on mental health changes among young adults. Nurs. Open.

[385] Machingura T., Shum D., Lloyd C., Murphy K., Rathbone E., Green H. (2022). Effectiveness of sensory modulation for people with schizophrenia: A multisite quantitative prospective cohort study. Aust. Occup. Ther. J..

[386] Marcus S., Chuang C., Ng-Mak D., Olfson M. (2017). Outpatient Follow-Up Care and Risk of Hospital Readmission in Schizophrenia and Bipolar Disorder. Psychiatr. Serv..

[387] Marneros A., Pillmann F., Wustmann T. (2012). Delusional disorders--are they simply paranoid schizophrenia?. Schizophr. Bull..

[388] O’Brien M.P., Zinberg J.L., Ho L., Rudd A., Cannon T.D. (2008). Family problem solving interactions and 6-month symptomatic and functional outcomes in youth at ultra-high risk for psychosis and with recent onset psychotic symptoms: A longitudinal study. Schizophr. Res..

[389] Patterson M., Rezansoff S., Currie L.B., Somers J. (2013). Trajectories of recovery among homeless adults with mental illness who participated in a randomised controlled trial of Housing First: A longitudinal, narrative analysis. BMJ Open.

[390] Peer J., Kupper Z., Long J., Brekke J., Spaulding W. (2007). Identifying mechanisms of treatment effects and recovery in rehabilitation of schizophrenia: Longitudinal analytic methods. Clin. Psychol. Rev..

[391] Petranovich C.L., Wade S., Taylor H., Cassedy A., Stancin T., Kirkwood M.W., Brown T.M. (2015). Long-Term Caregiver Mental Health Outcomes Following a Predominately Online Intervention for Adolescents with Complicated Mild to Severe Traumatic Brain Injury. J. Pediatr. Psychol..

[392] Prasad F., Hahn M., Chintoh A., Remington G., Foussias G., Rotenberg M., Agarwal S. (2023). Depression in caregivers of patients with schizophrenia: A scoping review. Soc. Psychiatry Psychiatr. Epidemiol..

[393] Racenstein J., Harrow M., Reed R.A., Martin E., Herbener E., Penn D. (2002). The relationship between positive symptoms and instrumental work functioning in schizophrenia: A 10year follow-up study. Schizophr. Res..

[394] Rössler W., Riecher-Rössler A., Angst J., Murray R., Gamma A., Eich D., Os J., Gross V.A. (2007). Psychotic experiences in the general population: A twenty-year prospective community study. Schizophr. Res..

[395] Sorkhou M., Bedder R.H., George T. (2021). The Behavioral Sequelae of Cannabis Use in Healthy People: A Systematic Review. Front. Psychiatry.

[396] Sudeck G., Höner O. (2011). Volitional Interventions within Cardiac Exercise Therapy (VIN-CET): Long-Term Effects on Physical Activity and Health-Related Quality of Life. Appl. Psychol. Health Well-Being.

[397] Tulloch A., Fearon P., David A. (2006). Social outcomes in schizophrenia: From description to action. Curr. Opin. Psychiatry.

[398] Walther S., Eisenhardt S., Bohlhalter S., Vanbellingen T., Müri R., Strik W., Stegmayer K. (2016). Gesture Performance in Schizophrenia Predicts Functional Outcome After 6 Months. Schizophr. Bull..

[399] Watzke S., Brieger P., Kuss O., Schoettke H., Wiedl K. (2008). A longitudinal study of learning potential and rehabilitation outcome in schizophrenia. Psychiatr. Serv..

[400] Winter L., Couwenbergh C., Weeghel J., Hasson-Ohayon I., Vermeulen J., Mulder C., Boonstra N., Klaver K., Oud M., Haan L. (2021). Changes in social functioning over the course of psychotic disorders-A meta-analysis. Schizophr. Res..

[401] Winter L., Jelsma A., Vermeulen J., Vellinga A., Weeghel J., Hasson-Ohayon I., Mulder C.L., Boonstra N., Veling W., Haan L. (2025). Interrelationships of Changes in Outcome Domains in Patients with Schizophrenia Spectrum Disorders: A Meta-Analysis. Acta Psychiatr. Scand..

[402] Świtaj P., Anczewska M., Chrostek A., Sabariego C., Cieza A., Bickenbach J., Chatterji S. (2012). Disability and schizophrenia: A systematic review of experienced psychosocial difficulties. BMC Psychiatry.

[403] Boehm J.K., Soo J., Zevon E.S., Chen Y., Kim E.S., Kubzansky L. (2018). Longitudinal Associations Between Psychological Well-Being and the Consumption of Fruits and Vegetables. Health Psychol..

[404] Chan R., Mak W., Chio F.H.N., Tong A.C.Y. (2018). Flourishing with Psychosis: A Prospective Examination on the Interactions Between Clinical, Functional, and Personal Recovery Processes on Well-being Among Individuals with Schizophrenia Spectrum Disorders. Schizophr. Bull..

[405] Chan K., Tsui J.K.C. (2025). Longitudinal impact of stigma resistance on mental health among individuals with mental disorders. Qual. Life Res..

[406] Davidson L. (2020). Recovering a sense of self in schizophrenia. J. Personal..

[407] Faugère M., Micoulaud-Franchi J., Alessandrini M., Richieri R., Faget-Agius C., Auquier P., Lançon C., Boyer L. (2015). Quality of life is associated with chronic inflammation in schizophrenia: A cross-sectional study. Sci. Rep..

[408] Fleury M., Grenier G., Bamvita J.M., Tremblay J., Schmitz N., Caron J. (2013). Predictors of quality of life in a longitudinal study of users with severe mental disorders. Health Qual. Life Outcomes.

[409] Fleury M., Grenier G., Bamvita J.M. (2015). Predictive typology of subjective quality of life among participants with severe mental disorders after a five-year follow-up: A longitudinal two-step cluster analysis. Health Qual. Life Outcomes.

[410] Friedman E., Ruini C., Foy C., Jaros L., Love G., Ryff C. (2019). Lighten UP! A Community-Based Group Intervention to Promote Eudaimonic Well-Being in Older Adults: A Multi-Site Replication with 6 Month Follow-Up. Clin. Gerontol..

[411] Fusar-Poli P., Rocchetti M., Sardella A., Avila A., Brandizzi M., Caverzasi E., Politi P., Ruhrmann S., McGuire P. (2015). Disorder, not just state of risk: Meta-analysis of functioning and quality of life in people at high risk of psychosis. Br. J. Psychiatry.

[412] Gardsjord E., Romm K.L., Friis S., Barder H., Evensen J., Haahr U., Hegelstad W., Joa I., Johannessen J.O., Langeveld J. (2016). Subjective quality of life in first-episode psychosis. A ten year follow-up study. Schizophr. Res..

[413] Gibson B., Rosser B.A., Schneider J., Forshaw M.J. (2023). The role of uncertainty intolerance in adjusting to long-term physical health conditions: A systematic review. PLoS ONE.

[414] Gondek D., Ning K., Ploubidis G., Nasim B., Goodman A. (2018). The impact of health on economic and social outcomes in the United Kingdom: A scoping literature review. PLoS ONE.

[415] Gurková E., Štureková L., Mandysova P., Šaňák D. (2023). Factors affecting the quality of life after ischemic stroke in young adults: A scoping review. Health Qual. Life Outcomes.

[416] Górna K., Jaracz K., Rybakowski F., Rybakowski J. (2008). Determinants of objective and subjective quality of life in first-time-admission schizophrenic patients in Poland: A longitudinal study. Qual. Life Res..

[417] He X., Migliorini C., Huang Z., Wang F., Zhou R., Chen Z., Xiao Y.N., Wang Q.W., Wang S., Harvey C. (2022). Quality of life in patients with schizophrenia: A 2-year cohort study in primary mental health care in rural China. Front. Public Health.

[418] Hoeksema L., Bruins J., Gangadin S., Meer L., Pijnenborg M., Visser E., Timmerman M., Castelein S. (2025). Association Between Premenstrual Disorders and Quality of Life: A Cross-Sectional Study of 8311 women in Sweden. Schizophr. Res..

[419] Kompier M., Ybema J., Janssen J., Taris T. (2009). Employment Contracts: Cross-sectional and Longitudinal Relations with Quality of Working Life, Health and Well-being. J. Occup. Health.

[420] Lagger N., Amering M., Sibitz I., Gmeiner A., Schrank B. (2018). Stability and mutual prospective relationships of stereotyped beliefs about mental illness, hope and depressive symptoms among people with schizophrenia spectrum disorders. Psychiatry Res..

[421] Lam L., Chang W., Grimmer K. (2023). Treatment effects of adjunct group music therapy in inpatients with chronic schizophrenia: A systematic review. Front. Psychiatry.

[422] Lambert M., Schimmelmann B., Schacht A., Karow A., Wagner T., Wehmeier P., Huber C., Hundemer H., Dittmann R., Naber D. (2008). Long-term patterns of subjective wellbeing in schizophrenia: Cluster, predictors of cluster affiliation, and their relation to recovery criteria in 2842 patients followed over 3 years. Schizophr. Res..

[423] Lara J., Godfrey A., Evans E., Heaven B., Brown L., Barron E., Rochester L., Meyer T., Mathers J. (2013). Towards measurement of the Healthy Ageing Phenotype in lifestyle-based intervention studies. Maturitas.

[424] Law H., Morrison A., Byrne R., Hodson E. (2012). Recovery from psychosis: A user informed review of self-report instruments for measuring recovery. J. Ment. Health.

[425] Law H., Shryane N., Bentall R., Morrison A. (2016). Longitudinal predictors of subjective recovery in psychosis. Br. J. Psychiatry.

[426] Linde J., Schmid M., Ruud T., Skar-Fröding R., Biringer E. (2022). Social Factors and Recovery: A Longitudinal Study of Patients with Psychosis in Mental Health Services. Community Ment. Health J..

[427] Luciano A., Bond G., Drake R. (2014). Does employment alter the course and outcome of schizophrenia and other severe mental illnesses? A systematic review of longitudinal research. Schizophr. Res..

[428] Magalhães E. (2024). Dual-factor Models of Mental Health: A Systematic Review of Empirical Evidence. Psychosoc. Interv..

[429] Mitsunaga-Ohmuro N., Ohmuro N. (2021). Longitudinal changes in personal recovery in individuals with psychotic disorders through hospitalisation in a psychiatric ward: Preliminary findings. BMC Psychiatry.

[430] Ng F., Ibrahim N., Franklin D., Jordan G., Lewandowski F., Fang F., Roe D., Rennick-Egglestone S., Newby C., Hare-Duke L. (2021). Post-traumatic growth in psychosis: A systematic review and narrative synthesis. BMC Psychiatry.

[431] Oles S.K., Fukui S., Rand K., Salyers M.P. (2015). The relationship between hope and patient activation in consumers with schizophrenia: Results from longitudinal analyses. Psychiatry Res..

[432] Petkari E., Pietschnig J. (2015). Associations of Quality of Life with Service Satisfaction in Psychotic Patients: A Meta-Analysis. PLoS ONE.

[433] Poon A.W.C., Harvey C., MacKinnon A., Joubert L. (2016). A longitudinal population-based study of carers of people with psychosis. Epidemiol. Psychiatr. Sci..

[434] Reeves D., Blickem C., Vassilev I., Brooks H., Kennedy A., Richardson G., Rogers A. (2014). The Contribution of Social Networks to the Health and Self-Management of Patients with Long-Term Conditions: A Longitudinal Study. PLoS ONE.

[435] Ritsner M., Arbitman M., Lisker A., Ponizovsky A. (2012). Ten-year quality of life outcomes among patients with schizophrenia and schizoaffective disorder II. Predictive value of psychosocial factors. Qual. Life Res..

[436] Rooijen G., Rooijen M., Maat A., Vermeulen J., Meijer C., Ruhé H., Haan L., Alizadeh B., Bartels-Velthuis A., Beveren N.V. (2019). Longitudinal evidence for a relation between depressive symptoms and quality of life in schizophrenia using structural equation modeling. Schizophr. Res..

[437] Rubinstein K. (2016). Treatment-resistant schizophrenia during life span: Epidemiology, outcomes and innovative M-Health treatments within M-RESIST Project. Eur. Psychiatry.

[438] Seib C., Whiteside E., Humphreys J., Lee K.A., Thomas P., Chopin L., Crisp G.J., O’Keeffe A.J., Kimlin M., Stacey A.T.E. (2014). A longitudinal study of the impact of chronic psychological stress on health-related quality of life and clinical biomarkers: Protocol for the Australian Healthy Aging of Women Study. BMC Public Health.

[439] Seow L.S.E., Ong C., Mahesh M., Sagayadevan V., Shafie S., Chong S., Subramaniam M. (2016). A systematic review on comorbid post-traumatic stress disorder in schizophrenia. Schizophr. Res..

[440] Slattery M., Attard H., Stewart V., Roennfeldt H., Wheeler A. (2020). Participation in creative workshops supports mental health consumers to share their stories of recovery: A one-year qualitative follow-up study. PLoS ONE.

[441] Smith M.J., Greenberg J. (2008). Factors contributing to the quality of sibling relationships for adults with schizophrenia. Psychiatr. Serv..

[442] Stuifbergen A., Seraphine A., Roberts G. (2000). An explanatory model of health promotion and quality of life in chronic disabling conditions. Nurs. Res..

[443] Tan X., Shahwan S., Satghare P., Chua B.Y., Verma S., Tang C., Chong S., Subramaniam M. (2019). Trends in Subjective Quality of Life Among Patients with First Episode Psychosis—A 1 Year Longitudinal Study. Front. Psychiatry.

[444] Thomas E.C., Despeaux K.E., Drapalski A.L., Bennett M. (2017). Person-Oriented Recovery of Individuals with Serious Mental Illnesses: A Review and Meta-Analysis of Longitudinal Findings. Psychiatr. Serv..

[445] Thomson L., Rees C. (2023). Long-term outcomes of the recovery approach in a high-security mental health setting: A 20 year follow-up study. Front. Psychiatry.

[446] Trockel M., Corcoran D., Minor L.B., Shanafelt T. (2020). Advancing Physician Well-Being: A Population Health Framework. Proc. Mayo Clin. Proc..

[447] Töbelmann L., Hahne I., Schulze T., Bergmann N., Fuchs L.M., Zierhut M., Hahn E., Böge K. (2023). Mechanisms of action and processes of yoga-based group intervention for inpatients with schizophrenia spectrum disorders–A longitudinal qualitative study. Front. Psychiatry.

[448] Usui K., Kirihara K., Tada M., Fujioka M., Koshiyama D., Tani M., Tsuchiya M., Morita S., Kawakami S., Kanehara A. (2022). The association between clinical symptoms and later subjective quality of life in individuals with ultra-high risk for psychosis and recent-onset psychotic disorder: A longitudinal investigation. Psychiatry Clin. Neurosci..

[449] Vilardaga R., Hayes S., Atkins D.C., Bresee C., Kambiz A. (2013). Comparing experiential acceptance and cognitive reappraisal as predictors of functional outcome in individuals with serious mental illness. Behav. Res. Ther..

[450] Westerhof G., Bohlmeijer E. (2014). Celebrating fifty years of research and applications in reminiscence and life review: State of the art and new directions. J. Aging Stud..

[451] Winter L., Jelsma A., Vermeulen J., Weeghel J., Hasson-Ohayon I., Mulder C.L., Boonstra N., Veling W., Haan L. (2024). Long-term Changes in Personal Recovery and Quality of Life Among Patients with Schizophrenia Spectrum Disorders and Different Durations of Illness: A Meta-analysis. Schizophr. Bull..

[452] Aanesen F., Berg R.C., Jørgensen I.L., Mohr B., Proper K.I., Lunde L.K. (2024). Employment and mental health in the working age population: A protocol for a systematic review of longitudinal studies. Syst. Rev..

[453] Anglin D.M., Corcoran C., Brown A.S., Chen H., Lighty Q., Brook J., Cohen P. (2012). Early Cannabis Use and Schizotypal Personality Disorder Symptoms from Adolescence to Middle Adulthood. Schizophr. Res..

[454] Barendse M., Lara G.A., Guyer A.E., Swartz J.R., Taylor S.L., Shirtcliff E., Tully L.M. (2023). Sex and pubertal influences on the neurodevelopmental underpinnings of schizophrenia: A case for longitudinal research on adolescents. Schizophr. Res..

[455] Bell J.S., Watson D.P., Griffin T., Martell S.C., Kay E., Hawk M., Ray B., Hudson M. (2025). Workforce outcomes among substance use peer supports: A scoping review of individual and organizational influences. Front. Public Health.

[456] Brand B., Willemse E., Hamers I., Sommer I.E. (2023). Evidence-Based Recommendations for the Pharmacological Treatment of Women with Schizophrenia Spectrum Disorders. Curr. Psychiatry Rep..

[457] Brouwers E. (2020). Social stigma is an underestimated contributing factor to unemployment in people with mental illness or mental health issues: Position paper and future directions. BMC Psychol..

[458] Burghart M., Backhaus S. (2024). The Long-Term Consequences of Family Violence Victimization: An Umbrella Review of Longitudinal Meta-Analyses on Child Maltreatment and Intimate Partner Violence. J. Fam. Violence.

[459] Cadenhead K., Minichino A., Kelsven S., Addington J., Bearden C., Cannon T., Cornblatt B., Mathalon D., McGlashan T., Perkins D. (2018). Metabolic abnormalities and low dietary omega 3 are associated with symptom severity and worse functioning prior to the onset of psychosis: Findings from the north American prodrome longitudinal studies consortium. Schizophr. Res..

[460] Carney C., Jones L., Woolson R. (2006). Medical comorbidity in women and men with schizophrenia. J. General. Intern. Med..

[461] Cates A.D., Catone G., Marwaha S., Bebbington P., Humpston C.S., Broome M. (2021). Self-harm, suicidal ideation, and the positive symptoms of psychosis: Cross-sectional and prospective data from a national household survey. Schizophr. Res..

[462] Degnan A., Berry K., Humphrey C., Bucci S. (2021). The relationship between stigma and subjective quality of life in psychosis: A systematic review and meta-analysis. Clin. Psychol. Rev..

[463] Drake R., Nordentoft M., Haddock G., Arango C., Fleischhacker W., Glenthøj B., Leboyer M., Leucht S., Leweke M., McGuire P. (2015). Modeling determinants of medication attitudes and poor adherence in early nonaffective psychosis: Implications for intervention. Schizophr. Bull..

[464] Dubreucq J., Plasse J., Franck N. (2021). Self-stigma in Serious Mental Illness: A Systematic Review of Frequency, Correlates, and Consequences. Schizophr. Bull..

[465] Dwyer D., Buciuman M., Ruef A., Kambeitz J., Dong M.S., Stinson C., Kambeitz-Ilankovic L., Degenhardt F., Sanfelici R., Antonucci L. (2022). Clinical, Brain, and Multilevel Clustering in Early Psychosis and Affective Stages. JAMA Psychiatry.

[466] Egan M., Tannahill C., Petticrew M., Thomas S. (2008). Psychosocial risk factors in home and community settings and their associations with population health and health inequalities: A systematic meta-review. BMC Public Health.

[467] Fervaha G., Foussias G., Agid O., Remington G. (2015). Motivational deficits in early schizophrenia: Prevalent, persistent, and key determinants of functional outcome. Schizophr. Res..

[468] Figueiredo I.C., Borgan F., Pasternak O., Turkheimer F., Howes O. (2022). White-matter free-water diffusion MRI in schizophrenia: A systematic review and meta-analysis. Neuropsychopharmacology.

[469] Fredrikson D., Boyda H., Tse L., Whitney Z., Pattison M.A., Ott F.J., Hansen L., Barr A. (2014). Improving Metabolic and Cardiovascular Health at an Early Psychosis Intervention Program in Vancouver, Canada. Front. Psychiatry.

[470] Garfin D.R., Thompson R.R., Holman E. (2018). Acute stress and subsequent health outcomes: A systematic review. J. Psychosom. Res..

[471] Giovannetti A., Solari A., Pakenham K. (2021). Effectiveness of a group resilience intervention for people with multiple sclerosis delivered via frontline services. Disabil. Rehabil..

[472] Glonti K., Gordeev V.S., Goryakin Y., Reeves A., Stuckler D., Mckee M., Roberts B. (2015). A Systematic Review on Health Resilience to Economic Crises. PLoS ONE.

[473] Goff D., Romero K., Paul J., Perez-Rodriguez M.M., Crandall D., Potkin S. (2016). Biomarkers for drug development in early psychosis: Current issues and promising directions. Eur. Neuropsychopharmacol..

[474] Guyer B., Ma S., Grason H., Frick K., Perry D.F., Sharkey A.B., Mcintosh J. (2009). Early childhood health promotion and its life course health consequences. Acad. Pediatr..

[475] Harder S., Davidsen K., MacBeth A., Lange T., Minnis H., Andersen M., Simonsen E., Lundy J.M., Nyström-Hansen M., Trier C. (2015). Wellbeing and resilience: Mechanisms of transmission of health and risk in parents with complex mental health problems and their offspring—The WARM Study. BMC Psychiatry.

[476] Harrow M., Jobe T., Tong L. (2021). Twenty-year effects of antipsychotics in schizophrenia and affective psychotic disorders. Psychol. Med..

[477] Heilbronner U., Malzahn D., Strohmaier J., Maier S., Frank J., Treutlein J., Mühleisen T.W., Forstner A., Witt S., Cichon S. (2015). A common risk variant in CACNA1C supports a sex-dependent effect on longitudinal functioning and functional recovery from episodes of schizophrenia-spectrum but not bipolar disorder. Eur. Neuropsychopharmacol..

[478] Hjorth P., Medici C., Juel A., Madsen N., Vandborg K., Munk-Jørgensen P. (2017). Improving quality of life and physical health in patients with schizophrenia: A 30-month program carried out in a real-life setting. Int. J. Soc. Psychiatry.

[479] Huang W.Y., Chen S.P., Pakpour A., Lin C.Y. (2018). The Mediation Role of Self-Esteem for Self-Stigma on Quality of Life for People with Schizophrenia: A Retrospectively Longitudinal Study. J. Pac. Rim Psychol..

[480] Jaarsma E., Smith B. (2017). Promoting physical activity for disabled people who are ready to become physically active: A systematic review. Psychol. Sport Exerc..

[481] Johnson S., Sathyaseelan M., Charles H., Jeyaseelan V., Jacob K. (2012). Insight, psychopathology, explanatory models and outcome of schizophrenia in India: A prospective 5-year cohort study. BMC Psychiatry.

[482] Jonas K.G., Lian W., Callahan J., Ruggero C., Clouston S., Reichenberg A., Carlson G., Bromet E., Kotov R. (2022). The Course of General Cognitive Ability in Individuals with Psychotic Disorders. JAMA Psychiatry.

[483] Katon W., Russo J., Heckbert S., Lin E., Ciechanowski P.S., Ludman E., Young B., Korff M.V. (2010). The relationship between changes in depression symptoms and changes in health risk behaviors in patients with diabetes. Int. J. Geriatr. Psychiatry.

[484] Kefford R., Trent R. (1989). The diagnostic applications of molecular biology. Med. J. Aust..

[485] Komatsu H., Ono T., Onoguchi G., Tomita H., Kakuto Y. (2021). Mediating effects of self-stigma and depression on the association between autistic symptoms and recovery in patients with schizophrenia-spectrum disorders: A cross-sectional study. BMC Psychiatry.

[486] Komatsu H., Onoguchi G., Silverstein S.M., Jerotić S., Sakuma A., Kanahara N., Kakuto Y., Ono T., Yabana T., Nakazawa T. (2023). Retina as a potential biomarker in schizophrenia spectrum disorders: A systematic review and meta-analysis of optical coherence tomography and electroretinography. Mol. Psychiatry.

[487] Koren D., Tzivoni Y., Schalit L., Adres M., Reznik N., Apter A., Parnas J. (2019). Basic self-disorders in adolescence predict schizophrenia spectrum disorders in young adulthood: A 7-year follow-up study among non-psychotic help-seeking adolescents. Schizophr. Res..

[488] Koster M., Mannsdörfer L., Pluijm M., Haan L., Ziermans T., Wingen G.V., Vermeulen J. (2024). The Association Between Chronic Tobacco Smoking and Brain Alterations in Schizophrenia: A Systematic Review of Magnetic Resonance Imaging Studies. Schizophr. Bull..

[489] Kotov R., Guey L., Bromet E., Schwartz J. (2010). Smoking in schizophrenia: Diagnostic specificity, symptom correlates, and illness severity. Schizophr. Bull..

[490] Kruse E.A., Dodell-Feder D. (2025). Schizophrenia spectrum stigma in healthcare: A systematic review. Front. Psychiatry.

[491] Kötter T., Tautphäus Y., Scherer M., Voltmer E. (2014). Health-promoting factors in medical students and students of science, technology, engineering, and mathematics: Design and baseline results of a comparative longitudinal study. BMC Med. Educ..

[492] Lee E.E., Liu J., Tu X., Palmer B., Eyler L., Jeste D. (2017). A Widening Longevity Gap between People with Schizophrenia and General Population: A Literature Review and Call for Action. Schizophr. Res..

[493] Lee E.E., Wu T.C., Ibrahim S., Dyne A.V., Tu X.M., Eyler L. (2025). Trajectories of Resilience-Related Traits and Their Impact on Health Outcomes in Schizophrenia: Results From a 4-Year Longitudinal Study. Schizophr. Bull..

[494] Lener M.S., Wong E., Tang C.Y., Byne W., Goldstein K., Blair N.J., Haznedar M.M., New A., Chemerinski E., Chu K. (2014). White matter abnormalities in schizophrenia and schizotypal personality disorder. Schizophr. Bull..

[495] Lester P., Liang L., Milburn N.G., Mogil C., Woodward K., Nash W., Aralis H., Sinclair M., Semaan A., Klosinski L. (2016). Evaluation of a Family-Centered Preventive Intervention for Military Families: Parent and Child Longitudinal Outcomes. J. Am. Acad. Child Adolesc. Psychiatry.

[496] Levy N., Selwyn D., Lobo D. (2020). Turning ’waiting lists’ for elective surgery into ’preparation lists. Br. J. Anaesth..

[497] Lien Y., Chang H.A., Kao Y., Tzeng N.S., Lu C.W., Loh C. (2016). Insight, self-stigma and psychosocial outcomes in Schizophrenia: A structural equation modelling approach. Epidemiol. Psychiatr. Sci..

[498] Lincoln T., Lüllmann E., Rief W. (2006). Correlates and long-term consequences of poor insight in patients with schizophrenia. A systematic review. Schizophr. Bull..

[499] Lindmark U., Ahlstrand I., Ekman A., Berg L., Hedén L., Källstrand J., Larsson M., Nunstedt H., Oxelmark L., Pennbrant S. (2020). Health-promoting factors in higher education for a sustainable working life—Protocol for a multicenter longitudinal study. BMC Public Health.

[500] Luo H., Li Y., Yang B., Chen J., Zhao P. (2022). Psychological interventions for personal stigma of patients with schizophrenia: A systematic review and network meta-analysis. J. Psychiatr. Res..

[501] Marcham L., Ellett L. (2024). Exposure to green spaces and schizophrenia: A systematic review. Psychol. Med..

[502] McFarlane A., Hooff M.V. (2009). Impact of childhood exposure to a natural disaster on adult mental health: 20-year longitudinal follow-up study. Br. J. Psychiatry.

[503] Mohamed S., Rosenheck R., McEvoy J., Swartz M., Stroup S., Lieberman J. (2009). Cross-sectional and longitudinal relationships between insight and attitudes toward medication and clinical outcomes in chronic schizophrenia. Schizophr. Bull..

[504] Mongan D., Perry B.I., Healy C., Susai S.R., Zammit S., Cannon M., Cotter D.R. (2024). Longitudinal Trajectories of Plasma Polyunsaturated Fatty Acids and Associations with Psychosis Spectrum Outcomes in Early Adulthood. Biol. Psychiatry.

[505] Muraleedharan M., Figueiredo D., Shrisunder R., Jadhav S. (2025). Approaches to health promotion in higher education: A scoping review (2014–2024). Health Promot. Perspect..

[506] Méndez-López F., Oliván-Blázquez B., Domínguez-García M., Bartolomé-Moreno C., Rabanaque I., Magallón-Botaya R. (2023). Protocol for an observational cohort study on psychological, addictive, lifestyle behavior and highly prevalent affective disorders in primary health care adults. Front. Psychiatry.

[507] Nair D. (2019). Measuring and Modifying Psychological Distress in CKD: New Insights and Next Steps. Kidney Med..

[508] Nair N., Xavier S.M., Rabouin D., Mohan G., Rangaswamy T., Ramachandran P., Joober R., Schmitz N., Malla A., Iyer S. (2024). Patient-reported outcome measures in early psychosis: A cross-cultural, longitudinal examination of the self-reported health and self-reported mental health measures in Chennai, India and Montreal, Canada. Schizophr. Res..

[509] Nettis M., Pergola G., Kolliakou A., O’Connor J., Bonaccorso S., David A., Gaughran F., Forti M., Murray R., Marques T. (2019). Metabolic-inflammatory status as predictor of clinical outcome at 1-year follow-up in patients with first episode psychosis. Psychoneuroendocrinology.

[510] Newcomb M.E. (2020). Romantic relationships and sexual minority health: A review and description of the Dyadic Health Model. Clin. Psychol. Rev..

[511] Nguyen T.T., Eyler L., Jeste D. (2018). Systemic Biomarkers of Accelerated Aging in Schizophrenia: A Critical Review and Future Directions. Schizophr. Bull..

[512] Orozco F.A., Cole D., Ibrahim S., Wanigaratne S. (2011). Health promotion outcomes associated with a community-based program to reduce pesticide-related risks among small farm households. Health Promot. Int..

[513] Osimo E., Perry B., Cardinal R., Lynall M., Lewis J.R., Kudchadkar A., Murray G., Perez J., Jones P.B., Khandaker G. (2020). Inflammatory and cardiometabolic markers at presentation with first episode psychosis and long-term clinical outcomes: A longitudinal study using electronic health records. Brain Behav. Immun..

[514] Palermo T. (2020). Pain prevention and management must begin in childhood: The key role of psychological interventions. Pain.

[515] Pellet J., Golay P., Nguyen A., Suter C., Ismailaj A., Bonsack C., Favrod J. (2019). The relationship between self-stigma and depression among people with schizophrenia-spectrum disorders: A longitudinal study. Psychiatry Res..

[516] Perry B., Stochl J., Upthegrove R., Zammit S., Wareham N., Langenberg C., Winpenny E.M., Dunger D., Jones P.B., Khandaker G. (2021). Longitudinal Trends in Childhood Insulin Levels and Body Mass Index and Associations with Risks of Psychosis and Depression in Young Adults. JAMA Psychiatry.

[517] Racioppi A., Sheinbaum T., Gross G.M., Ballespí S., Kwapil T., Barrantes-Vidal N. (2018). Prediction of prodromal symptoms and schizophrenia-spectrum personality disorder traits by positive and negative schizotypy: A 3-year prospective study. PLoS ONE.

[518] Rendina H.J., Gamarel K., Pachankis J., Ventuneac A., Grov C., Grov C., Parsons J. (2017). Extending the Minority Stress Model to Incorporate HIV-Positive Gay and Bisexual Men’s Experiences: A Longitudinal Examination of Mental Health and Sexual Risk Behavior. Ann. Behav. Med..

[519] Scott J.G., Matuschka L., Niemelä S., Miettunen J., Emmerson B., Mustonen A. (2018). Evidence of a Causal Relationship Between Smoking Tobacco and Schizophrenia Spectrum Disorders. Front. Psychiatry.

[520] Sims B.E., Nottelmann E., Koretz D., Pearson J. (2007). Prevention of depression in children and adolescents. Am. J. Prev. Med..

[521] Smakowski A., Hüsing P., Völcker S., Löwe B., Rosmalen J., Shedden-Mora M., Toussaint A. (2024). Psychological risk factors of somatic symptom disorder: A systematic review and meta-analysis of cross-sectional and longitudinal studies. J. Psychosom. Res..

[522] Smith T.E., Hull J., Huppert J., Silverstein S., Anthony D., McClough J.F. (2004). Insight and recovery from psychosis in chronic schizophrenia and schizoaffective disorder patients. J. Psychiatr. Res..

[523] Snijders C., Pries L., Sgammeglia N., Jowf G.I.A., Youssef N., Nijs L., Guloksuz S., Rutten B. (2018). Resilience Against Traumatic Stress: Current Developments and Future Directions. Front. Psychiatry.

[524] Spaniol L., Wewiorski N.J., Gagne C., Anthony W. (2002). The process of recovery from schizophrenia. Int. Rev. Psychiatry.

[525] Strathearn L., Giannotti M., Mills R., Kisely S., Najman J., Abajobir A. (2020). Long-term Cognitive, Psychological, and Health Outcomes Associated with Child Abuse and Neglect. Pediatrics.

[526] Struijs S., Jong P.D., Jeronimus B., Does W., Riese H., Spinhoven P. (2021). Psychological risk factors and the course of depression and anxiety disorders: A review of 15 years NESDA research. J. Affect. Disord..

[527] Sugawara N., Yasui-Furukori N., Sato Y., Saito M., Furukori H., Nakagami T., Ishioka M., Kaneko S. (2014). Dietary patterns are associated with obesity in Japanese patients with schizophrenia. BMC Psychiatry.

[528] Tessner K., Mittal V., Walker E. (2011). Longitudinal study of stressful life events and daily stressors among adolescents at high risk for psychotic disorders. Schizophr. Bull..

[529] Thabet A., Ghandi S., Barker E., Rutherford G., Malekinejad M. (2023). Interventions to enhance psychological resilience in forcibly displaced children: A systematic review. BMJ Glob. Health.

[530] Torgalsbøen A., Mohn C. (2018). T70. recovery in first–episode psychosis: Cognitive, clinical and resilience (personal resources) trajectories across 6 years. Schizophr. Bull..

[531] Veijola J., Guo J.Y., Moilanen J., Jääskeläinen E., Miettunen J., Kyllönen M., Haapea M., Huhtaniska S., Alaräisänen A., Mäki P. (2014). Longitudinal Changes in Total Brain Volume in Schizophrenia: Relation to Symptom Severity, Cognition and Antipsychotic Medication. PLoS ONE.

[532] Verdolini N., Vieta E. (2021). Resilience, prevention and positive psychiatry. Acta Psychiatr. Scand..

[533] Wartelsteiner F., Mizuno Y., Frajo-Apor B., Kemmler G., Pardeller S., Sondermann C., Welte A., Fleischhacker W., Uchida H., Hofer A. (2017). M128. Quality of Life in Patients with Schizophrenia: Its Associations with Resilience, Self-Esteem, Hopelessness, and Psychopathology. Schizophr. Bull..

[534] Waszczuk K., Rek-Owodziń K., Tyburski E., Mak M., Misiak B., Samochowiec J. (2021). Disturbances in White Matter Integrity in the Ultra-High-Risk Psychosis State—A Systematic Review. J. Clin. Med..

[535] Whiting D., Gulati G., Geddes J., Fazel S. (2021). Association of Schizophrenia Spectrum Disorders and Violence Perpetration in Adults and Adolescents from 15 Countries: A Systematic Review and Meta-Analysis. JAMA Psychiatry.

[536] Wilson P., Kemper K., Schubert C., Batra M., Staples B., Serwint J., McClafferty H.H., Mahan J. (2017). National Landscape of Interventions to Improve Pediatric Resident Wellness and Reduce Burnout. Acad. Pediatr..

[537] Xu Z., Lay B., Oexle N., Drack T., Bleiker M., Lengler S., Blank C., Müller M., Mayer B., Rössler W. (2018). Involuntary psychiatric hospitalisation, stigma stress and recovery: A 2-year study. Epidemiol. Psychiatr. Sci..

[538] Ziegelmann J., Lippke S. (2009). Introduction to the special section: Theory-based approaches to stress and coping—Emerging themes and contemporary research. Eur. Psychol..

